# Superseding β‐Diketiminato Ligands: An Amido Imidazoline‐2‐Imine Ligand Stabilizes the Exhaustive Series of B=X Boranes (X=O, S, Se, Te)

**DOI:** 10.1002/anie.202015553

**Published:** 2021-01-12

**Authors:** Hadi Dolati, Lars Denker, Bartosz Trzaskowski, René Frank

**Affiliations:** ^1^ Institute of Inorganic and Analytical Chemistry Technische Universität Braunschweig Hagenring 30 38106 Braunschweig Germany; ^2^ Centre of New Technologies University of Warsaw Banacha 2C 02-097 Warszawa Poland

**Keywords:** chalcogens, boron, Lewis acids, ligand design, structure elucidation

## Abstract

Boron reluctantly forms B=X (X=O, S, Se, Te) moieties, which has stimulated the quest for such species in the past few years. Based on the N,N′‐chelating β‐diketiminato ligand (HNacNac), a new amido imidazoline‐2‐imine ligand system (HAmIm) is presented, giving rise to the isolation of an exhaustive series of Lewis acid free, monomeric chalcogen B=X boranes with documented π‐bond character between boron and the chalcogen. The chalcogenoboranes are isoelectronic and isolobal to the respective ketones. The chemical behavior of the oxoborane (B=O) strongly resembles the classical carbonyl reactivity in C=O bonds. The improved stability provided by HAmIm arises from the formation of more‐stable five‐membered boron chelates versus the six‐membered NacNac analogues and from the imidazoline‐2‐imine moiety providing enhanced σ‐ and π‐donation. The HAmIm ligand class may supersede the widely employed NacNac system in certain applications.

## Graphical abstract

The new N,N′‐chelating amido imidazoline‐2‐imine ligand (HAmIm) was designed to supersede the widely employed β‐diketiminato ligand (HNacNac). The superior stability of the HAmIm system is exemplified for a series of chalcogenoboranes with confirmed B=X double bonds (X=O, S, Se, Te), which are isoelectronic and isolobal to ketones, including their heavier analogues.

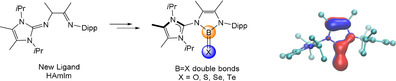

## Introduction

The C=O bond represents an archetypal functional group and is widely used in synthetic transformations. In an analogous fashion the heavier chalcogens have been exploited in the form of C=X derivatives (X=S, Se, Te), albeit their applications are less abundant compared to the C=O entity.^[1]^ In particular, the wide scope in reactivity associated with C=O bonds has stimulated attempts towards obtaining heavier elements of the tetrel family due to the enhanced reactivity expected for E=O moieties (E=Si, Ge, Sn, Pb). While C=O bonds in ketones can readily be obtained in monomeric fashion, the isoelectronic silanones R_2_Si=O^[2]^ and germanones R_2_Ge=O^[3]^ are stronger polarized and display a diminished π‐bond character, which renders them prone to spontaneous head‐to‐tail oligomerisation and thus require kinetic stabilization provided by bulky moieties.

**Scheme 1:**
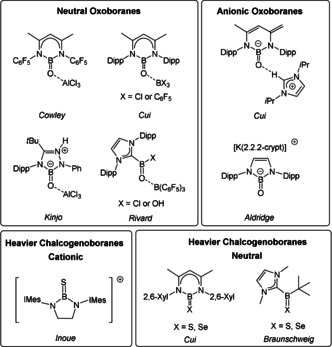
Top: Selected examples of neutral or anionic oxoboranes. Bottom: Selected examples of cationic or neutral heavier chalcogenoboranes. Dipp=2,6‐(*i*Pr)_2_C_6_H_3_. IMes=:C{N(2,4,6‐Me_3_C_6_H_2_)CH}_2_. 2,6‐Xyl=2,6‐Me_2_C_6_H_3_.

In contrast to the vast number of chalcogen C=X derivatives (X=O, S, Se, Te), carbon's neighbor element boron reluctantly forms chalcogenoboranes with the structural B=X double bond motif.^[4]^ In particular, oxoboranes R−B=O have captured early attention, but their monomeric species are highly elusive and have only been detected in the gas phase, in low‐temperature matrices, or by chemical trapping.^[5]^ In view of the electron deficient boron atom in oxoboranes R‐B=O, the isolation of such monomeric species should be feasible by the introduction of additional σ/π‐donating substituents in combination with steric congestion, which ultimately led to a handful of representative compounds prepared as bulk samples in the past decade, Scheme 1. The exploitation of *N*,*N′*‐chelating ligands at the boron center afforded the first examples, and the well‐established β‐diketiminato (NacNac) ligand was used by Cowley to afford a neutral oxoborane, which, however, required stabilization by AlCl_3_ acting as a strong Lewis acidic.^[6]^

Further Lewis acid‐stabilized neutral or anionic oxoboranes were later reported by Cui^[7]^ and Kinjo,^[8]^ and most noteworthy a Lewis acid free anionic oxoborane was only recently prepared by Aldridge.^[9]^ In alternative approaches the strong σ‐donation of NHCs was exploited by Rivard and afforded chloro or hydroxo functionalized oxoboranes.^[10]^ Braunschweig and Yamashita exploited the strategy of stabilizing entities with B−O multiple bonds within the coordination sphere of a transition metal.[^11^, ^12^] Examples of heavier B=X chalcogenoboranes (X=S, Se, Te) are even rarer than oxoboranes and strategies to stabilize them include (i) either *N*,*N′*‐chelation by bis imidazoline‐2‐imines by Inoue,^[13]^ NacNac type by Cui or Singh,^[14]^ or diamido ligands by Aldridge^[9]^ or (ii) NHC‐coordination by Braunschweig^[15]^ to afford neutral or cationic uncomplexed thio‐ or selenoboranes. In contrast to *N*,*N′*‐stabilized B=X boranes (X=S, Se), which are stable in the monomeric form, the NHC‐substituted species are prone to a rapid dimerization in solution with the formation of four‐membered boracycles. Telluroboranes with the structural B=Te double bond entity are extremely rare and only one example could be prepared by Braunschweig, and required stabilization with a Lewis acidic manganese core originating from the borylene complex it was formed from.^[15]^

**Scheme 2:**
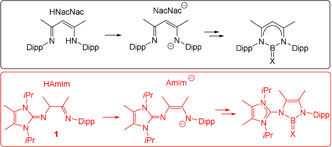
Comparison of HNacNac and HAmIm ligands. X=O, S, Se, Te. Dipp=2,6‐(*i*Pr)_2_C_6_H_3_.

Herein, we present a consistent contribution to the currently patchy area of ketone analogous boranes including heavier chalcogens with the special focus on the neutral species. So far there is no ligand system, which has afforded a complete series of chalcogenoboranes with the structural B=X entity (X=O, S, Se, Te), and the reported neutral oxoboranes and telluroboranes could only be isolated as complexes to (strong) Lewis acids. Therefore, we set out to design a novel ligand system, which affords the full scope of Lewis acid free, monomeric chalcogenoboranes. The obvious predominance of *N*,*N′*‐chelating NacNac ligands in attempts to stabilize the B=X entity can be traced back to the successful history of this ligand class in the production of low valent main group compounds and their applications in bond and substrate activation processes with prominent examples being NacNac stabilized Mg^I^, Al^I^ or Ga^I^ species.^[16]^ Based on the partial success achieved with NacNac type systems to isolate Lewis acid free monomeric B=S and B=Se species in the past, we envisaged that an improved *N*,*N′*‐donor ligand can lead to a better stability of the targeted series of B=X species, Scheme 2. The *N*,*N′*‐coordination in NacNac type ligands is obvious in their deprotonated anionic form NacNac^−^, which offers both σ/π‐donation via the amide and imine part. We present the new compound **1** (HAmIm), the formal backbone deprotonation of which yields the anionic amido imidazoline‐2‐imine ligand (AmIm^−^), and which can be viewed as an improved and more electron donating monoanionic ligand compared to the widely established NacNac ligand class. The advantages of the AmIm^−^ backbone over the NacNac^−^ system include (i) an increased σ/π‐donation of the imine entity due to the incorporation of the mesomerically active imidazoline‐2‐imine entity,^[17]^ a strategy which has first been recognized and widely exploited by Tamm in the stabilization of transition metal complexes^[18]^ and (ii) the formation of more stable five‐membered *N*,*N′*‐heterocycles instead of the six‐membered NacNac analogues, which also allows for a systematic variation of the bending angle at the central boron atom. The higher stability of five‐membered *N*,*N′*‐heterocycles over the six‐membered analogues is widely exemplified in the case of *N*‐heterocyclic Arduengo type carbenes, for which planar five‐membered structures are the most abundant and show the highest robustness.^[19]^ In view of the formation of five‐membered heterocycles the new monoanionic AmIm^−^ backbone also shows strong resemblance to dianionic diamido entities, for example, (RN‐CH=CH‐NR)^2−^, which are widely encountered in Arduengo type carbenes.

## Results and Discussion

**Scheme 3:**
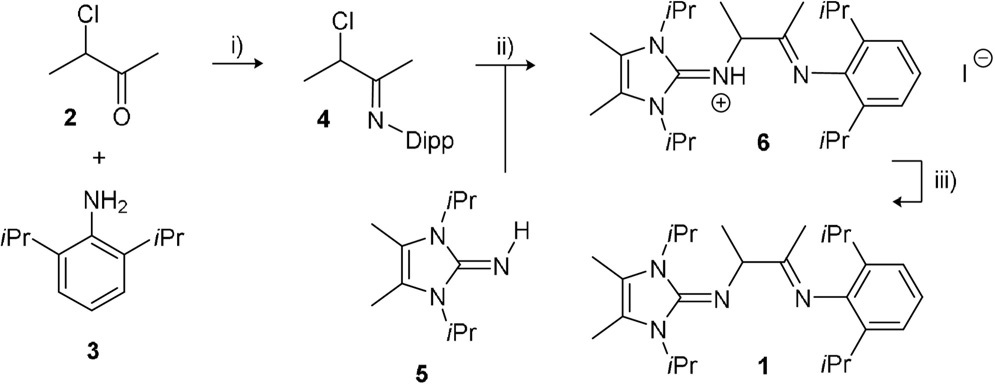
Key reagents and conditions. i) 0.6 equiv. TiCl_4_, CH_2_Cl_2_, 0 °C, then 4 equiv. NEt_3_, 0 °C, 1 h, then rt, 3 h. ii) 1 equiv. NaI, acetone, 48 h, rt. iii) two‐phase reaction hexanes/ MeOH + aqueous KOH (50 %). Dipp=2,6‐(*i*Pr)_2_C_6_H_3_.

The synthesis of HAmIm ligand **1** can be accomplished in three convenient steps involving facile organic transformations on gram scale starting from purchased or literature established compounds, Scheme 3. Thus, the reagents 3‐chloro‐2‐butanone **2** and 2,6‐diisopropyl amino benzene **3** were reacted to afford imine **4**. The reaction of the latter with imidazoline imine **5** at Finkelstein conditions led to the hydrogen iodide adduct HAmIm⋅HI **6**, the deprotonation of which afforded HAmIm **1**.

**Scheme 4:**
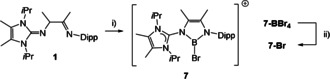
Key reagents and conditions. i) 6 equiv. BBr_3_, exc. Me_2_C=CMe_2_, hexanes, 15 min, rt. ii) 2 equiv. AgBF_4_, CH_2_Cl_2_, 5 min, rt, then Et_2_O, 3 h, rt. Dipp=2,6‐(*i*Pr)_2_C_6_H_3_.

The addition of **1** as a solution in hexanes to a solution of BBr_3_ in the same solvent afforded the ionic product **7‐BBr_4_** in high purity, Scheme 4. The reaction occurs with the extrusion of hydrogen bromide and the C=C double bond formation in the backbone.

**Figure 1:**
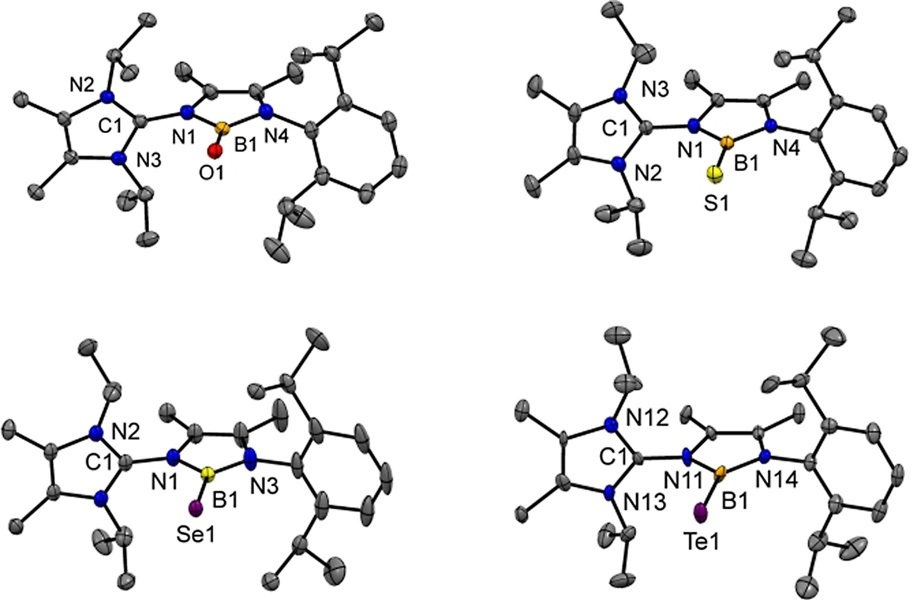
Molecular structures of compounds **10**–**13** from X‐ray crystallographic analysis.^[24]^ Hydrogen atoms and the lattice solvent molecules (benzene) for **11**–**13** are omitted for clarity. Thermal ellipsoids are presented at the 50 % level of probability. Bond distances and bond angles are reported in Å or degree (°), respectively. Compound **10**: O1‐B1 1.2867(16), N1‐B1 1.5208(17), N4‐B1 1.4896(17), O1‐B1‐N4 132.32(12), O1‐B1‐N1 128.67(11), N4‐B1‐N1 99.00(10). Compound **11**: S1‐B1 1.7546(12), N1‐B1 1.4796(14), N4‐B1 1.4493(14), N4‐B1‐N1 100.91(9), N4‐B1‐S1 131.29(8), N1‐B1‐S1 127.79(8). Compound **12**: The molecule is located on a crystallographic mirror plane: Se1‐B1 1.909(2), N1‐B1 1.469(3), N3‐B1 1.430(3), N3‐B1‐N1 100.92(17), N3‐B1‐Se1 132.04(16), N1‐B1‐Se1 127.04(15). Compound **13**: The asymmetric unit contains three crystallographically independent mole‐cules, one of which is depicted. Te1‐B1 2.151(6), N11‐B1 1.467(7), N14‐B1 1.436(8), N14‐B1‐N11 101.7(4), N14‐B1‐Te1 133.0(4), N11‐B1‐Te1 125.2(4).

**Scheme 5:**
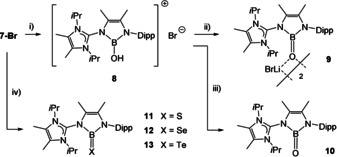
Key reagents and conditions. i) 1 equiv. H_2_O, 2 equiv. NEt_3_, CH_2_Cl_2_, 2 h, rt, then 2 equiv. CaH_2_, CH_2_Cl_2_, 1 h, rt. ii) 1 equiv. LiHMDS, THF, 30 min, rt. iii) 3 equiv KHMDS, 3 equiv. 2.2.2‐crypt, THF, rt. iv) for S, Se: 2 equiv. Li_2_X (X=S or Se), (CH_2_OMe)_2_, 50 °C overnight, then 1 equiv. 12‐crown‐4, benzene; for Te: 2 equiv. Li_2_Te, (CH_2_OMe)_2_, rt, 48 h. Dipp=2,6‐(*i*Pr)_2_‐C_6_H_3_.

The order of reagent addition as well as the presence of an electron‐rich alkene (i.e. 2,3‐dimethyl‐2‐butene) as a trapping reagent for hydrogen bromide was found to be crucial for this step. Since the presence of the anion BBr_4_^−^ would impede clean reactions of **7‐BBr_4_**, the latter was treated with AgBF_4_ to decompose the anion BBr_4_^−^, which afforded the more convenient bromide **7‐Br**. The reaction of **7‐BBr_4_** with AgBF_4_ was optimized with ^11^B{^1^H} NMR monitoring. The incremental addition of AgBF_4_ led to a decrease of the narrow signal at −24.2 ppm for BBr_4_^−^, and complete disappearance required ca. 2 equiv. of AgBF_4_. The ^11^B{^1^H} NMR spectra gave rise to the formation of several volatile boron halide species including BBr_3_ (38.0 ppm), BBr_2_F (29.6 ppm) and BBrF_2_ (19.4 ppm), which could be removed in vacuo. Compounds **7‐BBr_4_** and **7‐Br** were fully characterized including X‐ray crystallographic analysis, see section SI. In **7‐Br** the tri‐coordinate boron atom resonates at 21.2 ppm (ω_1/2_=170 Hz). Compound **7‐Br** was considered as an appropriate precursor to produce the B=X species supported by means of the AmIm^−^ ligand. Thus, in approaches towards the oxoborane species compound **7‐Br** was subsequently reacted with a stoichiometric amount of water in the presence of triethylamine, which afforded the ionic hydroxyborane **8**, Scheme 5. The deprotonation of the hydroxyl group in **8** was performed with LiHMDS, and afforded the neutral oxoborane **9** as a dimeric adduct with LiBr. Since the removal of LiBr to produce the uncomplexed oxoborane **10** was hampered due to the inherent oxophilic nature of the lithium cation, compound **8** was deprotonated with KHMDS in the presence of 2.2.2‐cryptand. The sequestration of the potassium salt formed as a side product allowed for the isolation of the first Lewis acid free neutral oxoborane species **10**. While the ^11^B{^1^H} chemical shifts of **8**–**10** are undiagnostic and display similar values [22.9 ppm (ω_1/2_=520 Hz) for **3**, 21.6 ppm (ω_1/2_=700 Hz) for **4**, 21.4 ppm (ω_1/2_=600 Hz) for **5**], the true nature of these products could unambiguously be authenticated by X‐ray crystallography, see section SI for **8** and **9**, Figure 1 for **10**.

For facile access to heavier B=X species (X=S, Se, Te) precursor **2‐Br** was reacted with the lithium chalcogenides Li_2_X in 1,2‐dimethoxyethane, Scheme 4. In the cases of thio‐ (**11**) and selenoborane (**12**) the crude product required treatment with the complexation reagent 12‐crown‐4 to remove the side product LiBr prior to the product extraction with benzene. The ^11^B{^1^H} chemical shifts of **11**–**13** respond with a slight downfield shift compared to **2‐Br** [35.2 ppm (ω_1/2_=320 Hz) for **11**, 35.8 ppm (ω_1/2_=330 Hz) for **12**, 30.2 ppm (ω_1/2_=680 Hz) for **13**]. The structures of compounds **11**–**13** were unequivocally confirmed by X‐ray crystallography, Figure 1.

**Figure 2:**
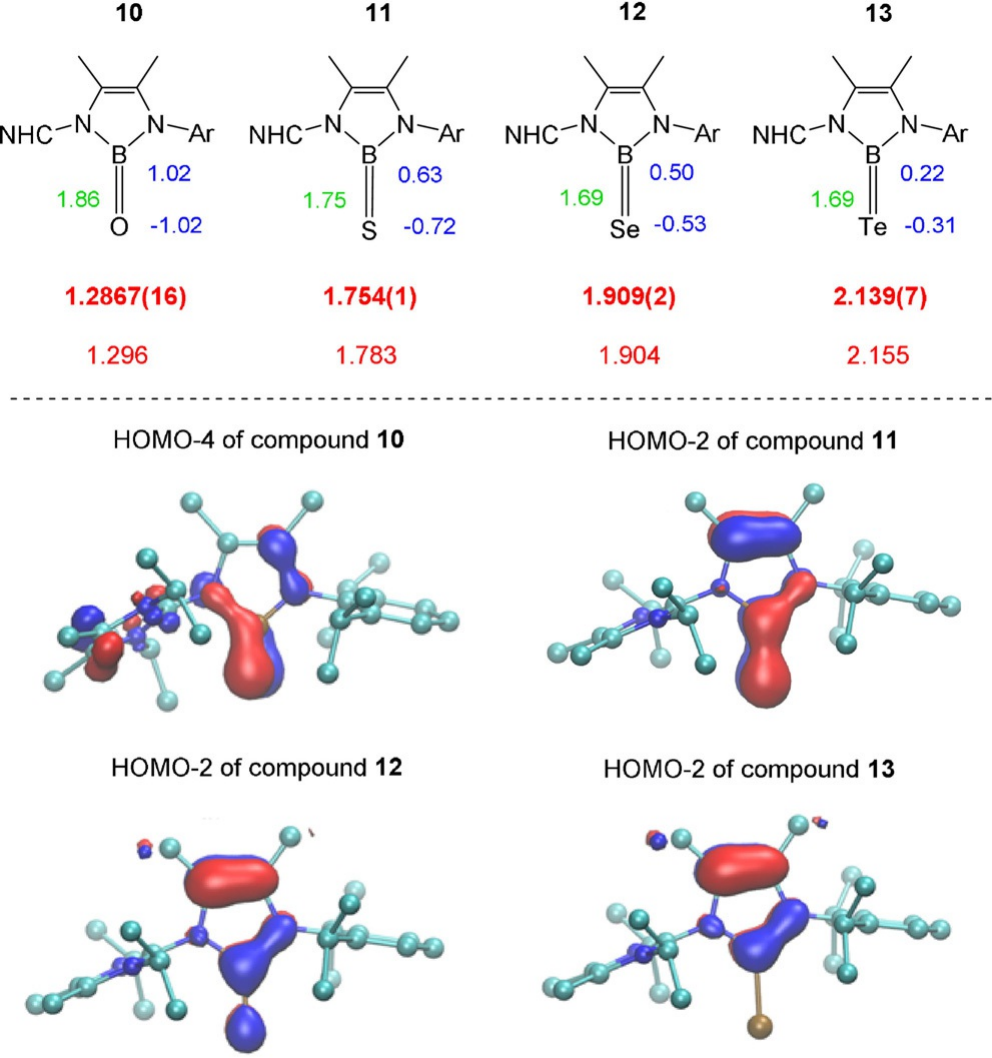
Top: Trend representation of compounds **10**–**13**. Crystallographic bond lengths in Å (red, bold), calculated bond lengths in Å (red, thin), Wiberg bond indices (WBI, green) and NPA charges (blue). Bottom: Representative canonical molecular orbitals of compounds **10** and **11**, which demonstrate the B=X multiple bond character, which diminishes with the heavier chalcogen. NHC=C{N(*i*Pr)CMe}_2_, Ar=2,6‐(*i*Pr)_2_C_6_H_3_.

With compounds **10**–**13** at hand an exhaustive structural series of Lewis acid free neutral chalcogenoboranes has been realized for the first time, Figure 2. In all compounds the boron center adopts the trigonal planar geometry. The B⋅⋅⋅O distance is significantly shortened upon deprotonation of the parent hydroxyborane **8** [1.353(9) Å] compared to the LiBr adduct **9** [1.3040(17) Å, 4 %] and the free oxoborane **10** [1.2867(16) Å, 5 %]. In particular, the latter B=O bond length is the shortest ever reported distance for neutral oxoboranes, which is most likely due to absence of the oxygen coordinated Lewis acid, present in all other previous examples. Only a marginally shorter B=O length is found in the anionic oxoborane species [1.273(8) Å] by Aldridge.^[9]^ The B=S [1.754(1) Å] and B=Se [1.909(2) Å] bond distances in **11** and **12** cover the region of other previously reported thio‐ and selenoboranes, albeit the B=Se distance is by far the longest ever reported. Compound **13** contains the first Lewis acid free B=Te double bond, which is marginally longer [2.139(7) Å] than in the only other reported Lewis acid stabilized B=Te structural motif [2.100(4) Å] by Braunschweig.^[15]^ This counterintuitive finding can be rationalized based on the presence of two π‐donating nitrogen substituents at the boron atom in **13** in contrast to the previously reported telluroborane, which retains a slightly longer bond B=Te double bond length. DFT calculations were performed at different levels of theory, and a highly accurate reproduction of the structural parameters, as obtained from the X‐ray crystallographic analysis, was found for the ωB97X‐D density functional with the triple‐zeta basis set. In particular, the measured B=X bond distances are in excellent agreement with experimental data and reflect the expected increase with the heavier chalcogen, Figure 2.

The Wiberg bond index (WBI) has frequently been employed to assess the multiple bond character of the B=X bonds (X=O, S, Se, Te). For a comparison of our compounds **10**–**13** with previously reported systems all compounds (including the published examples) were calculated on the same computational level [see section the Supporting Information (SI)]. The WBI is herein found to continuously decrease with the heavier chalcogen element from 1.86 in **10** to 1.69 in **13**, and the values >1.60 and justify the formulation as true B=X bonds with a significant double bond character for all four species, Figure 2. The decrease of the WBI values in this series indicates diminished π–π interactions between the boron atom and heavier chalcogen atoms. Compound **10** remarkably displays the highest value (WBI=1.86) ever reported for a B=O bond in oxoboranes, which can be rationalized by the absence of oxygen‐coordinated Lewis acids. Other previously reported neutral (Kinjo: WBI=1.44, Rivard: WBI=1.54)[^8^, ^10^] or anionic (Aldridge: WBI=1.80)^[9]^ oxoboranes feature lower values, which indicates the highest B=O double bond character in compound **10**. The WBI for the B=S bond in **11** (WBI=1.75) is marginally lower than the value reported for the cationic thioborane by Inoue (WBI=1.87)^[13]^ and neutral thioborane of Cui (WBI=1.91)^[14]^ (probably due increased π‐donation exerted by the HAmIm ligand) but is higher than for the anionic thioborane reported by Aldridge (WBI=1.69).^[9]^ The WBI of the B=Te bond in compound **8** (WBI=1.69) resembles the value of 1.82 reported for the telluroborane coordinated to a manganese core.^[15]^ The NPA charges at both boron and the chalcogen atom decrease with the heavier chalcogen, which indicates the strongest polarization for the B=O bond versus other members of triel family. The double bond character in **10**–**13** is also obvious from the visualization of the canonical molecular orbitals, which display π–π interactions between boron and the chalcogen atoms as exemplified for compounds **10** and **11**, Figure 2. Further orbital plots are displayed in the SI.

Since IR‐spectroscopy is a widely employed tool for the characterization of the stretch C=X vibrations (X=O, S, Se, Te), we also recorded IR‐spectra for our series of ketone analogous chalcogenoboranes **10**–**13** and compared them with values predicted from DFT‐calculations. For compound **5** the significant band at 1667 cm^−1^ for the stretch B=O vibration matches the DFT‐predicted value of 1651 cm^−1^, and as expected is higher than in reported Lewis acid‐coordinated neutral oxoboranes (Kinjo: $\tilde{\nu }$ =1636 cm^−1^, Rivard: $\tilde{\nu }$ =1636 cm^−1^).[^8^, ^10^, ^20^] In the case of compounds **11**–**13** the DFT‐computed wavenumbers were all found at ca. 1280 cm^−1^, which would be in accordance with the observed bands at ca. 1270 cm^−1^ for each compound. However, we hesitate to interpret these experimentally obtained bands as the respective B=X (X=S, Se, Te) stretch vibration due to their occurrence in the IR fingerprint region, in which with an intense overlap with numerous scaffold vibrations occurs, so that an unambiguous assignment cannot be given at this stage.

**Scheme 6:**
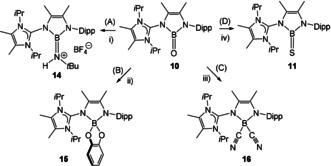
Carbonyl analogous reactivity of oxoborane **10**. Key reagents and conditions. i) 3 equiv. *t*BuNH_2_, MgSO_4_, (CH_2_OMe)_2_, 2 h, rt, then 1 equiv. HBF_4_⋅OEt_2_, benzene. ii) 1.2 equiv. catechol [1,2‐(HO)_2_C_6_H_4_], MgSO_4_, (CH_2_OMe)_2_, 6 h, rt. iii) 2.2 equiv. Me_3_SiCN, toluene, 6 h, rt. iv) ex. H_2_S, MgSO_4_, (CH_2_OMe)_2_, 6 h, rt. Dipp=2,6‐(*i*Pr)_2_‐C_6_H_3_.

Given the elaborated structural and bond theoretical analogy of the boranes **10**–**13** compared to ketones and their heavier analogues we set out to investigate their chemical behavior and concentrated our investigation on the reactivity of B=O vs. C=O bonds. We investigated four archetypal textbook examples of well documented carbonyl type reactivity in aldehydes or ketones and found the chemical behavior of oxoborane **10** to remarkably resemble the carbonyl chemistry, Scheme 6.

- Transformations of C=O bonds include reactions with primary amines to form imines in a condensation process, which is usually catalyzed by weak Lewis acids. We found that oxoborane **10** reacts with *t*BuNH_2_ as a representative amine to form the respective boraimine. The reaction necessitated the presence of MgSO_4_ to remove the stoichiometric amount of water formed. The boraimine was found to be sensitive and decomposed upon standing (ca. 6 hours). However, it could be trapped upon treatment with HBF_4_⋅OEt_2_ in the form of its protonated iminium salt **14**, which can be regarded as an imino compound formed from oxoborane **10** akin to organic imines produced from carbonyl compounds.
- Reactions of carbonyl compounds with chelating diols (at acidic conditions) are known to afford cyclic acetals or ketals with a change of the coordination number at carbon from three to four. Thus, the reactivity of oxoborane **10** towards catechol as a representative bifunctional 1,2‐hydroxy compound was studied in the presence of MgSO_4_ as a water trapping reagent. The ^11^B{^1^H} NMR chemical shift gives rise to the formation of compound **15** (12.0 ppm, ω_1/2_=47 Hz) diagnostic for a four‐coordinate boron center, and **15** can be viewed as a boraketal formed form the B=O entity in oxoborane **10**.
- The addition of HCN or Me_3_SiCN to carbonyl compounds is a facile transformation to afford cyanhydrines. The reaction of oxoborane **10** necessitated 2 equiv. of Me_3_SiCN for the complete conversion of the starting material and gave rise to the formation of the dicyanated four‐coordinated boron species **16** with an ^11^B{^1^H} NMR chemical shift of −8.5 ppm (ω_1/2_=54 Hz).^[21]^ In analogy to carbonyl compounds **16** is formed with cleavage of the double bond character of the B=O bond. However, the reaction did not halt at the cyanhydrine step as usually observed for ketones but proceeded to a dicyanated boron center. Although we did not perform any further investigation, we consider the silyl ether (Me_3_Si)_2_O as an obvious product in this reaction.
- The conversion of ketones to thioketones can be accom‐plished with H_2_S in the presence of catalytic amounts of HCl. The reaction of oxoborane **10** with H_2_S (excess) and in the presence of MgSO_4_ afforded thioborane **11** as indicated by a comparison of ^1^H and ^11^B{^1^H} NMR spectra of the material obtained with confirmed samples of **11**. The water trapping agent MgSO_4_ was found to be crucial since in its absence no formation of **11** was detected.

**Figure 3:**
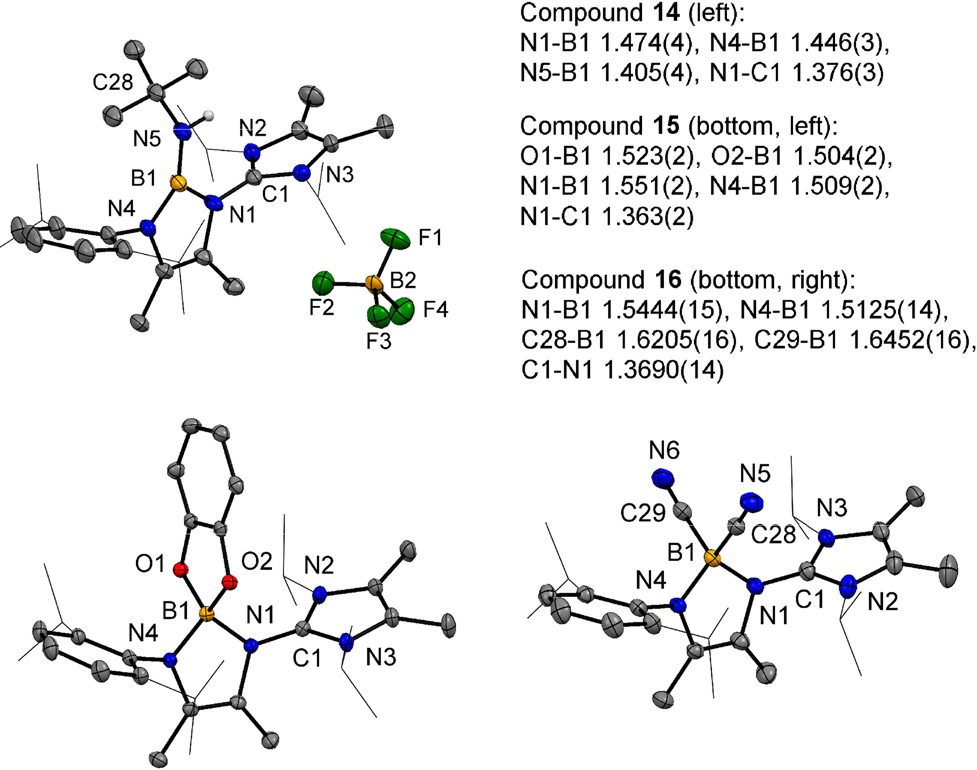
Molecular structures of compounds **14**–**16** from X‐ray crystallographic analysis. Carbon bound hydrogen atoms are omitted for clarity. Thermal ellipsoids are presented at the 50 % level of probability. Bond distances are stated in Å.

Besides the conclusive analytical data the nature of compounds **14**–**16** was unambiguously authenticated by X‐ray crystallo‐graphic analysis, Figure 3. In particular, the formulation of compound **14** as a protonated boraimine with a N=B double bond is based on the observation of the shortest distance N5−B1 of 1.405(4) Å compared to all other N−B bonds [N1−B1 1.474(4) Å, N4−B1 1.446(3) Å] in the cation. The molecular structures of compounds **15** and **16** retain the expected four‐coordinate geometry at boron. The reactivity of **10** is accompanied by a bond scission process of the B=O moiety. This is obvious for the formation of **11**, **14** and **16** but also for **15** assuming that both oxygen atoms originate from the starting material catechol. While B−O single bonds are considered to be thermodynamically extremely stable (B−OR bond dissociation energy ≈536 kJ mol^−1^),^[22]^ there are no reported values for the B=O double bond.^[23]^ The examples aforementioned demonstrate that the cleavage of the B=O entity in **10** can be induced with mild reagents when the oxygen containing side product is captured in a thermodynamically stable form, that is, water bound to a drying agent (MgSO_4_) or as silyl ether [(Me_3_Si)_2_O]. However, a comprehensive statement for the thermodynamic stability of the B=O bond cannot be given at this stage. To a certain extent the chemical behavior of **10** parallels the reactivity of Aldridge's anionic oxoborane.^[9]^ However, while the latter has predominantly been demonstrated to react as an oxide transfer agent for the O^2−^ anion (presumably due to the negative charge) of this oxoborane, we find our system **10** to display strong resemblance to carbonyl compounds as indicated by the routes (A)–(D), which also underpins the nature of **10** as ketone analogous borane from the standpoint of chemical reactivity.

## Conclusion

Herein, we have synthesized an exhaustive series of Lewis acid free, neutral B=X type boranes (X=O, S, Se, Te). Noteworthy, the respective neutral oxo‐ and telluroborane are now reported in their Lewis acid absent form for the first time. All chalcogenoboranes are isoelectronic and isolobal to ketones R_2_C=X and their heavier chalocogen analogues and were found to be stable in solution for at least one week without any signs of dimerization or decomposition. The analogy to ketones is proven by structural studies including X‐ray crystallographic analysis and the π‐multiple‐bond character for all B=X bonds is documented by theoretical calculations, in particular by the Wiberg bond index. In addition, the chemical behavior of the B=O bond in the oxoborane strongly resembles the classical carbonyl reactivity in C=O bonds as demonstrated by the formations of a boraimine, boraketal, dicyano borane and thioborane. The remarkable stability of the exhaustive series of B=X boranes can be traced back to the new HAmIm ligand, which we introduce as a superseding ligand to replace the widely employed β‐diketiminato ligand (HNacNac). Key advantages of the HAmIm over the HNacNac ligand include better σ/π‐donation and the formation of more stable five‐membered *N*,*N′*‐heterocycles, and we propose the HAmIm system as an improved ligand over the established HNacNac systems to a broader chemical community working in related fields.

## Conflict of interest

The authors declare no conflict of interest.

## Supporting information

As a service to our authors and readers, this journal provides supporting information supplied by the authors. Such materials are peer reviewed and may be re‐organized for online delivery, but are not copy‐edited or typeset. Technical support issues arising from supporting information (other than missing files) should be addressed to the authors.

Supplementary
