# Supplementary material for: Superseding β‐Diketiminato Ligands: An Amido Imidazoline‐2‐Imine Ligand Stabilizes the Exhaustive Series of B=X Boranes (X=O, S, Se, Te)
Source: Angew Chem Int Ed Engl. 2021 Jan 12;60(9):4633–9. doi: 10.1002/anie.202015553 (PMC7986232; doi:10.1002/anie.202015553)
Supplement: Supplementary file 1 — Supplementary [file ANIE-60-4633-s001.pdf]

Angewandte Chemie
Eine Zeitschrift der Gesellschaft Deutscher Chemiker

Supporting Information

Superseding β-Diketiminato Ligands: An Amido Imidazoline-2-Imine Ligand Stabilizes the Exhaustive Series of B=X Boranes (X = O, S, Se, Te)

Hadi Dolati, Lars Denker, Bartosz Trzaskowski, and René Frank**

anie_202015553_sm_miscellaneous_information.pdf

Abstract: While the C=X bond (X = O, S, Se, Te) in ketones and their heavier chalcogen analogues is an established structural entity, car-bon´s neighbor element boron reluctantly forms the respective B=X moieties, which has stimulated the quest for such species in the past few years. Based on the partial success achieved in this area by means of the *N,N´*-chelating *β*-diketiminato ligand (HNacNac), we present a new amido imidazoline-2-imine ligand system (HAmIm), which gives rise to the isolation of an exhaustive series of Lewis acid free, monomeric chalcogeno B=X boranes with documented π-bond character between boron and the chalcogen. The chalcogenobo-ranes are isoelectronic and isolobal the respective ketones. The chemical behavior of the oxoborane (B=O) strongly resembles the classical carbonyl reactivity in C=O bonds as demonstrated by four typical textbook examples including imine and ketal formation. The improved stability provided by the HAmIm ligand system arises (i) from the formation of more stable five-membered boron chelates vs. the six-membered NacNac analogues and (ii) from the incorporation of an imidazoline-2-imine moiety providing enhanced σ- and π-donation. We, therefore, propose that the new HAmIm ligand class may supersede the widely employed NacNac system in certain applications.

1. Synthetic and Analytical Procedures

1.1. General Information

All manipulations were performed under dry argon atmosphere using Schlenk techniques or in a glove box (M. Braun 200B model) unless stated otherwise. Solvents were purified and dried using a Solvent Purification System (M. Braun) and stored over molecular sieves (3−4 Å). All commercially available compounds (TCI, abcr, deutero, Sigma Aldrich) were used without further purification. Deuterated solvents were dried over sodium (C_6D_6, THF-d_8) or CaH_2 (CD_2Cl_2, $CDCl_3$), distilled under argon and stored over molecular sieves (3−4 Å). Compounds **5** [1] and Li_2X (X = Se, Te) [2] were prepared according to literature methods.

NMR spectra were recorded on Bruker Avance II-300, Avance III-HD, Avance III-400 and AVII-500 spectrometer. The chemical shifts (δ) are reported in parts per million (ppm).
The residual solvent peak (C_6HD_5, δ = 7.16 ppm, THF-HD_7, δ = 1.72, 3.58 ppm, $CHCl_3$, δ = 7.26 ppm, $CHDCl_2$, δ = 5.30 ppm) is used for the referencing of the ^{1}H-NMR spectra.
The ^{13}C spectra are internally calibrated by using the ^{13}C resonances of the solvent peaks (C_6D_6, δ = 128.06 ppm, THF-D_8, δ = 25.31, 67.21 ppm, $CDCl_3$, δ = 77.16 ppm, CD_2Cl_2, δ = 53.80 ppm).
For ^{11}B-NMR spectra an external calibration with $BF_3{\cdot}Et_2O$ was used.
Coupling constants are stated in Hertz (Hz), multiplicities are defined as br (broad), s (singlet), d (doublet), t (triplet), q (quartet), qu (quintet), sept (septet) or m (multiplet).
If necessary, 2D-NMR experiments (H,H-COSY, H,C-HSQC, H,C-HMBC) were used to aid the assignment of the signals.

IR spectra were recorded on a Bruker Vertex 70 with the KBr disc transmission technique.

Mass spectra were recorded on a Finnigan MAT 8400-MSS I instrument (for electro spray ionization, ESI) or on a Finnigan MAT 4515 instrument (electron impact mode, EI) and are reported as the m/z ratio (in Da).

Elemental analyses were accomplished by combustion and gas chromatographic analysis using a VarioMICRO Tube and HW detection. Values are reported in weight-%.

*1.2. Synthesis of HAmIm **1**.*

*1.2.1. Synthesis of Compound **4**.*

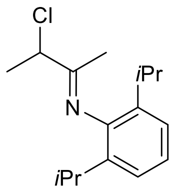

4

3-Chloro-2-butanone (**2**, 10.55 g, 99.0 mmol) was added to a solution of 2,6-diisopropylaniline (**3**, 18.43 g, 104.0 mmol, 1.05 eq.) in CH_2Cl_2 (150 mL), and the mixture was cooled to 0 °C. A solution of titanium tetrachloride (6.40 mL, 5.90 mmol, 0.60 eq.) in CH_2Cl_2 (30 mL) was added dropwise with immediate change of the color from yellowish to deep red. Triethylamine (55.20 mL, 396.0 mmol, 4.00 eq.) was added dropwise to the reaction mixture, and stirring was continued for 1 h at 0 °C and then 3 h at room temperature. The dropwise addition of water (30 mL) quenched the remaining titanium tetrachloride, and the mixture was filtered through a pad of celite. Water (200 mL) was added to the filtrate. The aqueous phase was extracted in a separator funnel with CH_2Cl_2 (3 × 100 mL). The combined organic phases were dried over magnesium sulfate, filtered, and concentrated. The crude product was purified by column chromatography (pentanes / ethyl acetate, 5:95, v/v) to afford a yellowish oil of **4** in analytically pure form (19.10 g, 73 %). R_f = 0.37.

^{1}H NMR (CD_2Cl_2, 400 MHz, 293 K): δ = 1.27 (3 H, d, $^3J_{HH}$ = 0.6 Hz, CH_3 in *i*Pr), 1.29 (3 H, d, $^3J_{HH}$ = 0.6 Hz, CH_3 in *i*Pr), 1.30 (3 H, d, $^3J_{HH}$ = 1.8 Hz, CH_3 in *i*Pr), 1.32 (3 H, d, $^3J_{HH}$ = 1.8 Hz, CH_3 in *i*Pr), 1.92 (3 H, d, $^3J_{HH}$ = 6.8 Hz, CH*CH₃*), 1.93 (3 H, s, NC*CH₃*), 2.85 (2 H, sept, $^3J_{HH}$ = 6.9 Hz, CH in *i*Pr), 4.88 (1 H, q, $^3J_{HH}$ = 6.7 Hz, C*H*CH₃), 7.19 (1 H, t, $^3J_{HH}$ = 7.6 Hz, aryl-CH), 7.26 (2 H, d, $^3J_{HH}$ = 7.6 Hz, aryl-CH).

^{13}C{^{1}H} NMR (CD_2Cl_2, 100 MHz, 293 K): δ = 16.7 (NC*CH₃*), 22.3 (CH*C*H₃), 23.2, 23.3, 23.6, 23.7 (all four CH_3 in *i*Pr), 28.5, 28.6 (both *C*H in *i*Pr), 61.4 (*C*HCl), 123.6, 123.6, 124.4 (all three aryl-CH), 136.3, 136.4, 145.7 (all three aryl-C), 169.1 (N*C*CH₃).

MS (EI): m/z = 265.2 (30 %) $[M]^+$, 202.2 (100 %) $[M-CH_3CHCl]^+$.

Elemental analysis. Calculated for $C_{16}H_{24}ClN$: C 72.29, H 9.10, N 5.27. Found: C 72.18, H 8.97, N 5.35.

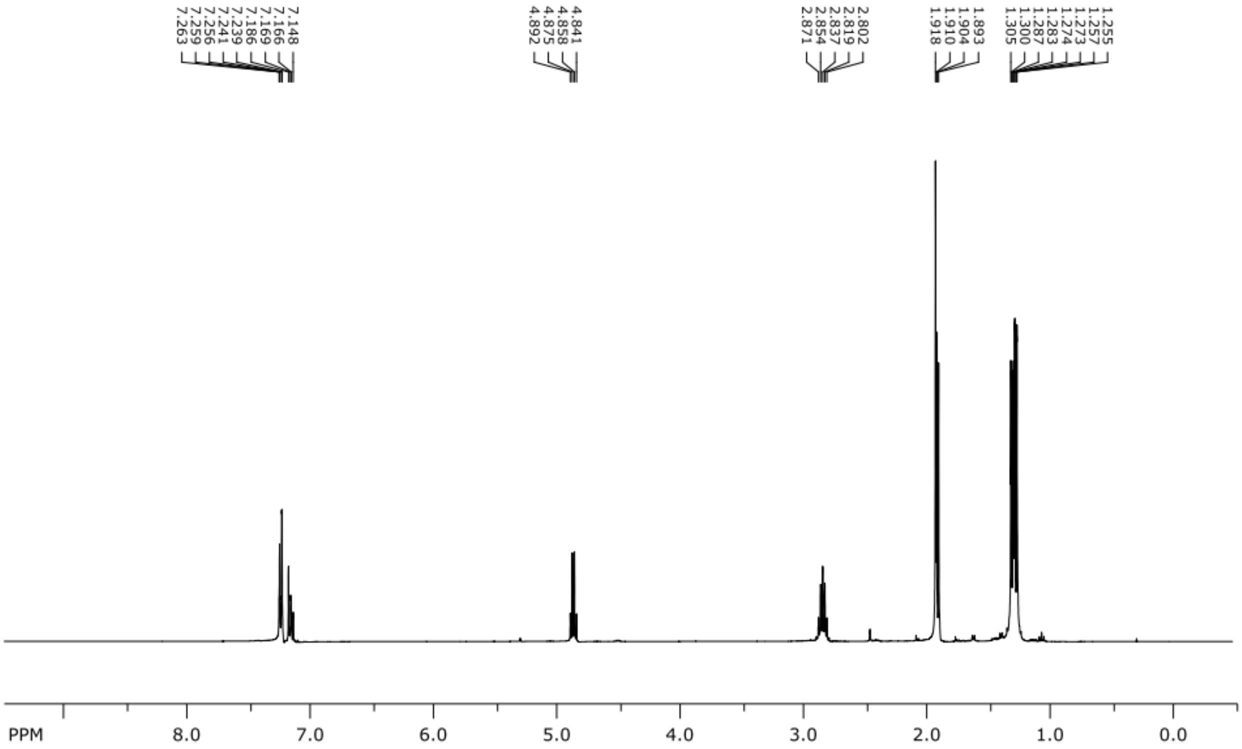

Figure S1. ^{1}H NMR (CD_2Cl_2, 400 MHz, 293 K) of compound **4**.

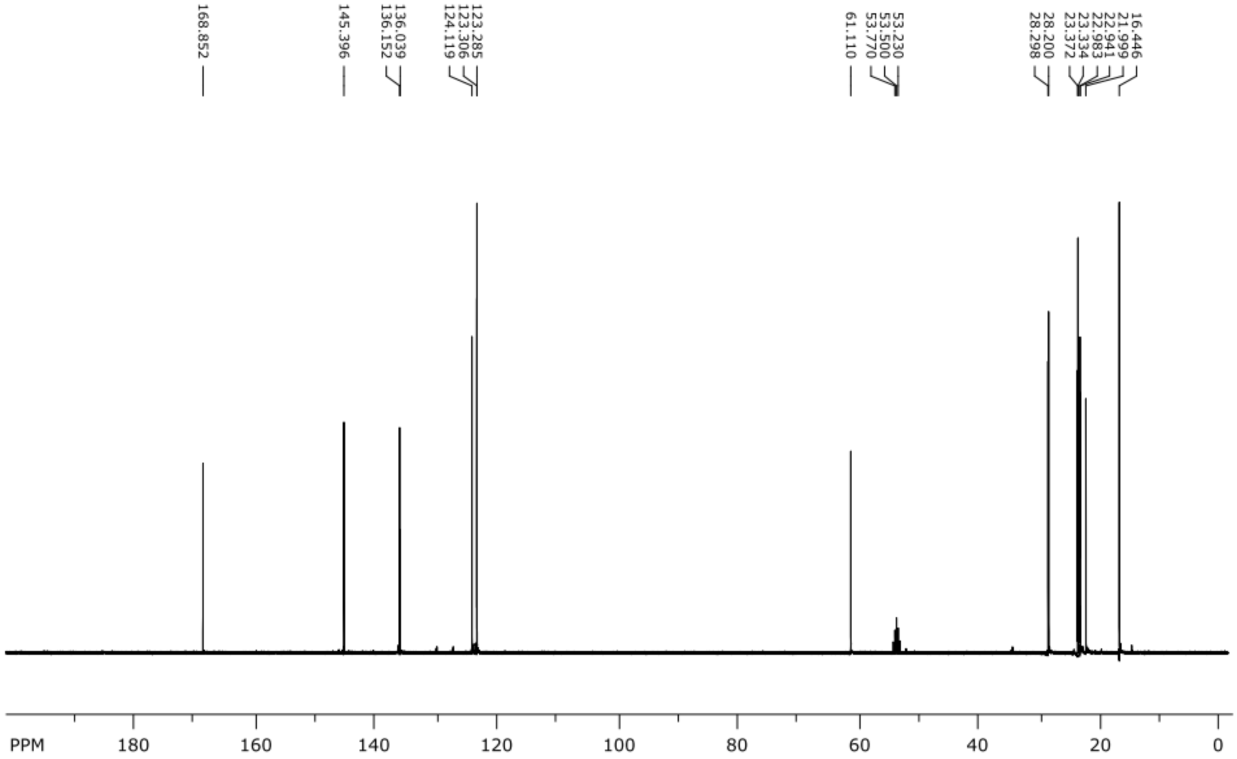

Figure S2. $^{13}C\{^{1}H\}$ NMR (CD_2Cl_2, 100 MHz, 293 K) of compound **4**.

*1.2.2. Synthesis of Compound **6**, HAmIm·HI.*

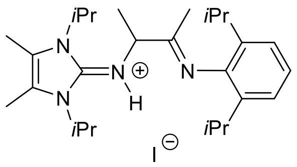

6

Imidazoline-2-imine (**5**, 1.00 g, 5.12 mmol, 1 eq.) was added to a solution of compound **4** (1.36 g, 5.12 mmol, 1 eq.) in anhydrous acetone (30 mL). Sodium iodide (767 mg, 5.12 mmol, 1 eq.) was added to the reaction mixture with rapid dissolution of the solid. The clear solution was stirred for 48 h at room temperature with continuous precipitation of colorless sodium chloride. The solvent was removed under vacuum and water (30 mL) was added to yellowish honey-like residue. After stirring for 1 h chloroform (30 mL) was added, and the mixture was transferred into a separatory funnel. The aqueous phase was extracted with chloroform (2 × 20 mL) and was dried over magnesium sulfate and filtered. The filtrate was dried under vacuum to yield **6** (2.26 g, 80 %) as a white powder. Crystals suitable for X-ray crystallography (Figure S3) and elemental analysis were obtained by diffusion of *n*-pentane into a solution of X in dichloromethane.

^{1}H NMR (CD_2Cl_2, 500 MHz, 293 K): δ = 1.00 (3 H, d, $^{3}J_{HH}$ = 6.9 Hz, CH_3 in *i*Pr), 1.08 (3 H, d, $^{3}J_{HH}$ = 6.9 Hz, CH_3 in *i*Pr), 1.11 (3 H, d, $^{3}J_{HH}$ = 6.9 Hz, CH_3 in *i*Pr), 1.14 (3 H, d, $^{3}J_{HH}$ = 6.9 Hz, CH_3 in *i*Pr), 1.54 (6 H, d, $^{3}J_{HH}$ = 7.1 Hz, CH_3 in *i*Pr), 1.56 (6 H, d, $^{3}J_{HH}$ = 7.1 Hz, CH_3 in *i*Pr), 1.65 (3 H, d, $^{3}J_{HH}$ = 6.8 Hz, CH$C\mathit{H_3}$), 1.81 (3 H, s, N=CCH_3), 2.28 (6 H, s, C$\mathit{H_3}$, imidazoline backbone), 2.28 (1 H, sept, $^{3}J_{HH}$ = 6.9 Hz, C*H*Me_2 in Dipp), 2.61 (1 H, sept, $^{3}J_{HH}$ = 6.9 Hz, C*H*Me_2 in Dipp), 4.50 (1 H, qu, $^{3}J_{HH}$ = 6.4 Hz, C*H*CH_3 in Dipp), 5.03 (2 H, sept, $^{3}J_{HH}$ = 7.0 Hz, C*H*Me_2 in imidazoline), 6.61 (1 H, d, $^{3}J_{HH}$ = 5.7 Hz, N*H*), 7.04−7.13 (3 H, m, aryl-CH).

^{13}C{^{1}H} NMR (CD_2Cl_2, 125 MHz, 293 K): δ = 10.5 (N=C$\mathit{C}H_3$), 18.4 (N^{+}HCH$\mathit{C}H_3$), 19.9 (C$\mathit{C}H_3$, imidazoline backbone), 21.4 (*C*H_3 in *i*Pr), 21.9 (*C*H_3 in *i*Pr), 23.0 (*C*H_3 in *i*Pr), 23.4 (*C*H_3 in *i*Pr), 23.5 (*C*H_3 in *i*Pr), 23.7 (*C*H_3 in *i*Pr), 28.1 (C*H*Me_2 in Dipp), 50.5 (*C*HMe_2 in imidazoline), 60.5 (*C*HCH_3), 123.4 (aryl-CH), 123.5 (*C*CH_3, imidazoline backbone), 123.5, 124.4 (both aryl-CH), 136.3, 136.5, 143.2 (all three aryl-C), 144.5 (N_3*C*), 171.2 (N=*CC*$\mathit{H_3}$).

MS (ESI+): m/z = 425.3 (100 %) $[M{-}I]^{+}$

Elemental analysis. Calculated for $C_{27}H_{45}IN_4$: C 58.69, H 8.21, N 10.14. Found: C 58.42, H 7.95, N 9.95.

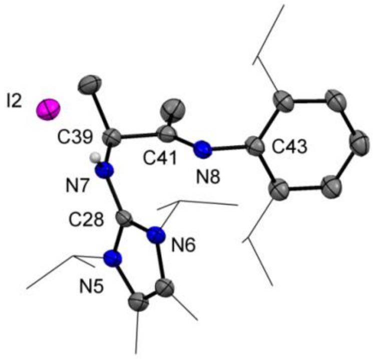

Figure S3. Molecular structure of compound **6**. Thermal ellipsoids are presented at the 50 % level of probability. Carbon bound hydrogen atoms are omitted for clarity. The asymmetric unit contains two crystallographically independent ion pairs, one of which is depicted. Selected bond distances and bond angles are reported in Å or degree (°), respectively. C28−N5 1.344(5), C28−N6 1.346(5), C28−N7 1.371(5), C39−N7 1.472(5), C39−C41 1.515(6), C41−N8 1.267(5), C43−N8 1.425(5), N8−C41−C39 119.1(4).

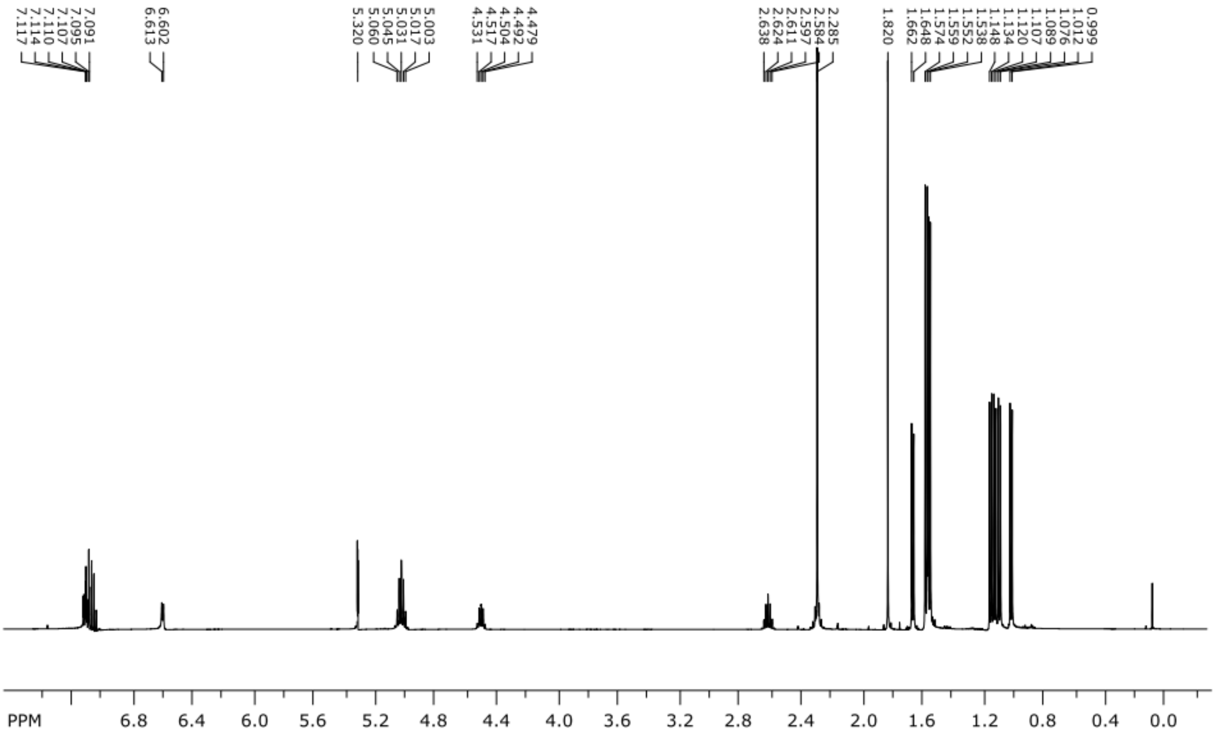

Figure S4. ^1H NMR (CD_2Cl_2, 500 MHz, 293 K) of compound **6**.

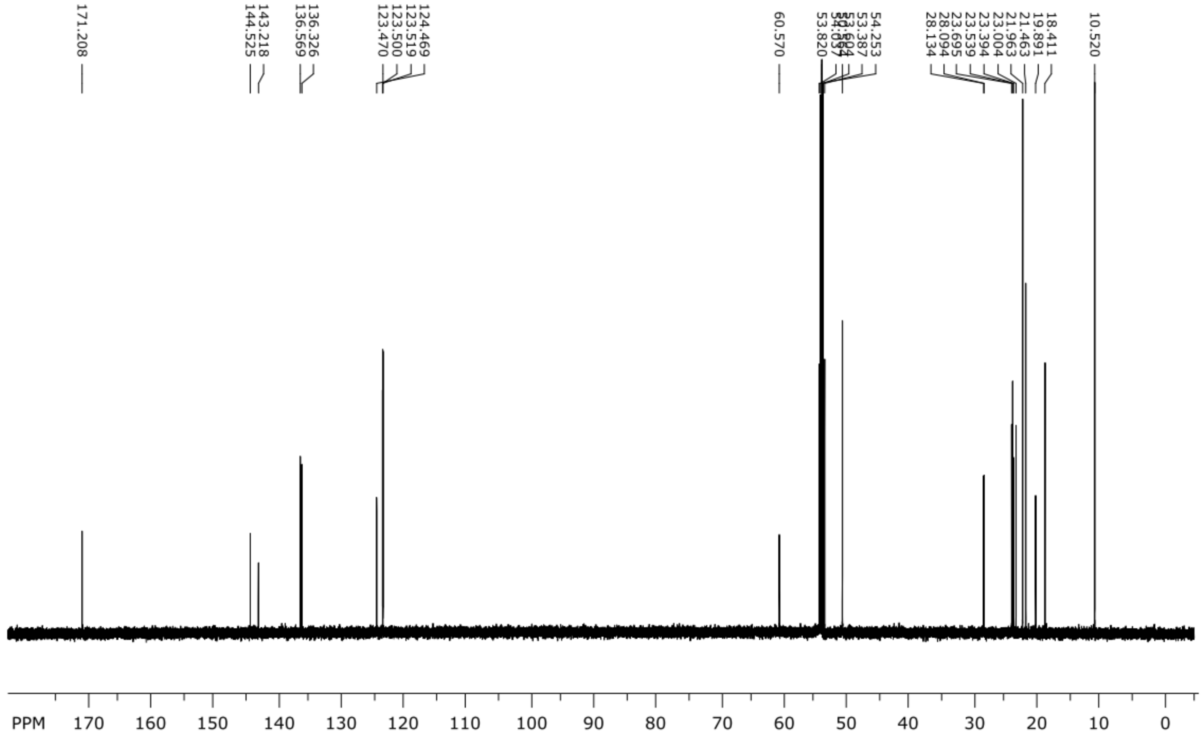

Figure S5. $^{13}C\{^1H\}$ NMR (CD_2Cl_2, 125 MHz, 293 K) of compound **6**.

*1.2.3. Synthesis of Compound **1**, HAmIm.*

Hydrogen iodide adduct HAmIm·HI (**6**, 3.00 g, 5.36 mmol) was dissolved in methanol (10 mL). A portion of hexanes (40 mL) was added followed by a solution of potassium hydroxide (7 mL, 50 % in water) with vigorous stirring. The two-phase mixture was stirred at room temperature for 20 min. The layers were allowed to settle for 5 min. The top phase (hexanes) was separated via cannula and dried over magnesium sulfate. The solvent was removed *in vacuo* (assisted by freeze-drying), which afforded HAmIm **1** (1.07 g, 75%) as an analytically pure, off-white powder.

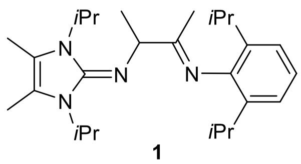

1

HAmIm

^{1}H NMR (CD_2Cl_2, 400 MHz, 293 K): δ = 1.10 (3 H, d, $^3J_{HH}$ = 6.9 Hz CH_3 in *i*Pr), 1.12 (3 H, d, $^3J_{HH}$ = 7.0 Hz CH_3 in *i*Pr), 1.16 (3 H, d, $^3J_{HH}$ = 7.0 Hz, CH_3 in *i*Pr), 1.17 (3 H, d, $^3J_{HH}$ = 6.9 Hz, CH_3 in *i*Pr), 1.38 (6 H, d, $^3J_{HH}$ = 7.1 Hz, CH_3 in *i*Pr), 1.39 (6 H, d, $^3J_{HH}$ = 7.1 Hz, CH_3 in *i*Pr), 1.42 (3 H, d, $^3J_{HH}$ = 6.5 Hz, CH*CH₃*), 1.74 (3 H, s, N=CCH_3), 2.03 (6 H, s, CH_3, imidazoline backbone), 2.76 (1 H, sept, $^3J_{HH}$ = 7.0 Hz, C*H*Me_2 in Dipp), 2.86 (1 H, sept, $^3J_{HH}$ = 6.8 Hz, C*H*Me_2 in Dipp), 4.50 (2 H, sept, $^3J_{HH}$ = 6.8 Hz, C*H*Me_2 in imidazoline), 4.56 (1 H, q, $^3J_{HH}$ = 6.0 Hz, *CH*CH_3), 6.99−7.19 (3 H, m, aryl-CH).

^{13}C{^{1}H} NMR (CD_2Cl_2, 100 MHz, 293 K): δ = 11.1 (N=C*CH₃*), 16.1 (=NC*CH₃*), 21.1 (CCH_3, imidazoline backbone), 21.4 (*C*H_3 in *i*Pr), 21.6 (*C*H_3 in *i*Pr), 23.3 (*C*H_3 in *i*Pr), 23.5 (*C*H_3 in *i*Pr), 23.8 (*C*H_3 in *i*Pr), 28.1 (*C*HMe_2 in Dipp), 28.2 (*C*HMe_2 in Dipp), 47.5(*C*HMe_2 in imidazoline), 62.6 (*C*HCH_3), 116.7 (*C*CH_3, imidazoline backbone), 123.2, 123.3, (both three aryl-CH), 136.5, 136.7, 147.1 (all three aryl-C), 150.9 (N_3*C*), 177.6 (N=*C*C*H₃*).

MS (EI): m/z = 424.3 (10 %) $[M]^+$.

Elemental analysis. Calculated for $C_{27}H_{44}N_4$: C 76.36, H 10.44, N 13.19. Found: C 76.41, H 10.41, N 12.60.

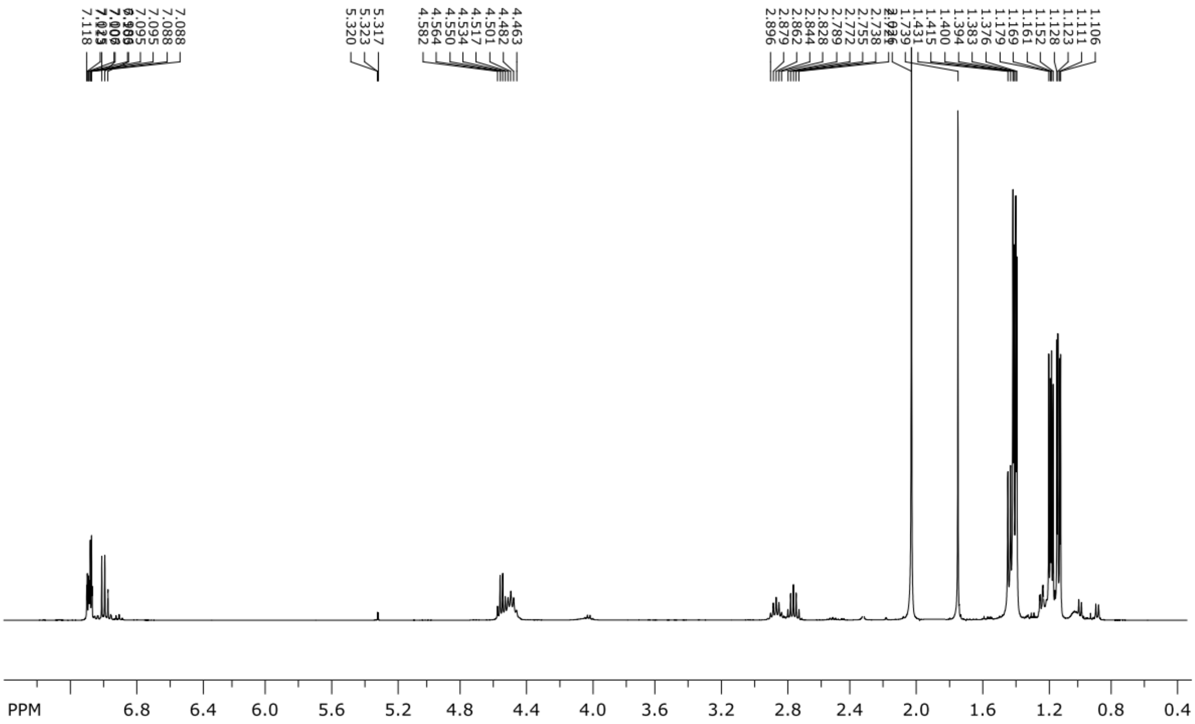

Figure S6. ^{1}H NMR (CD_2Cl_2, 400 MHz, 293 K) of compound **1**.

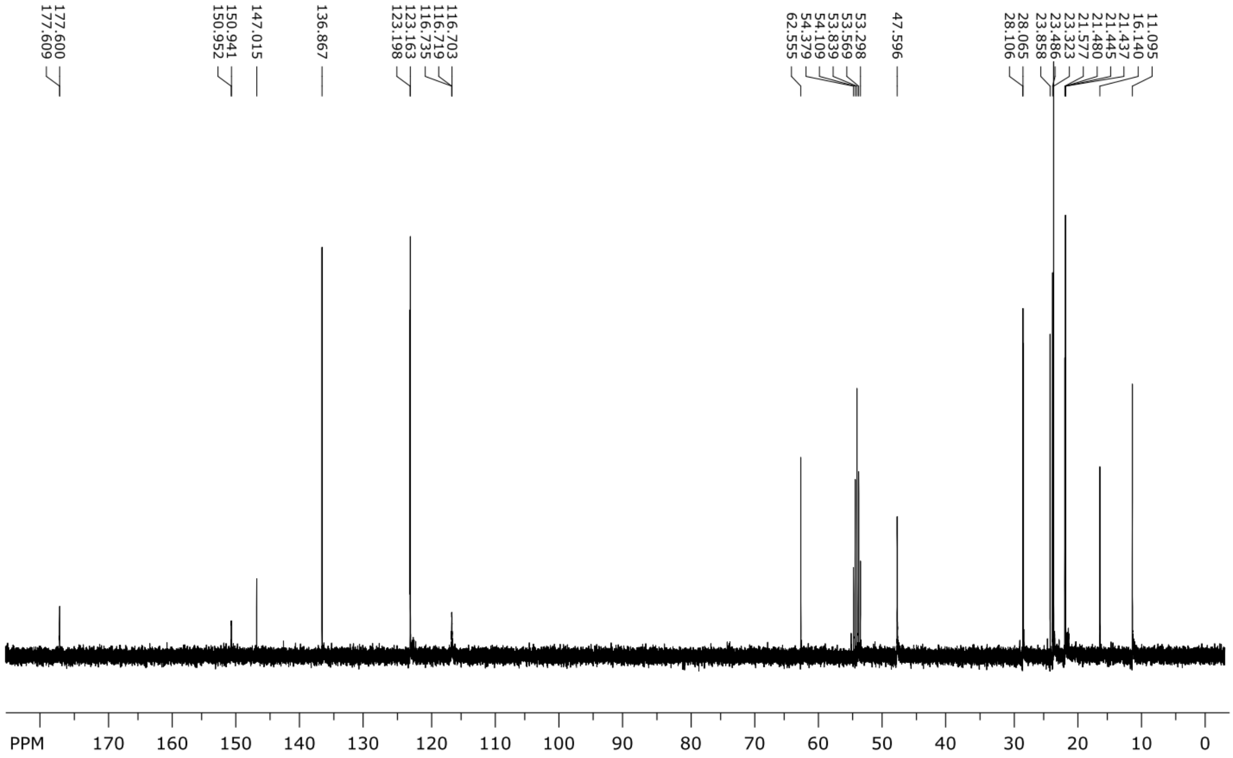

Figure S7. $^{13}C\{^1H\}$ NMR (CD_2Cl_2, 100 MHz, 293 K) of compound **1**.

*1.3. Synthesis of Compounds **7-BBr4** and **7-Br**.*

*1.3.1. Synthesis of Compound **7-BBr4**.*

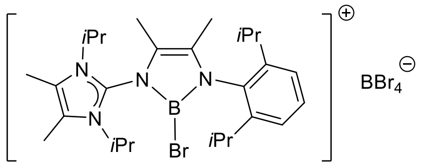

7-BBr4

A side arm flask (the neck should be large in diameter) was charged with hexanes (100 mL), 2,3-dimethyl-2-butene ($Me_2C{=}CMe_2$, dried over CaH_2 and distilled, 6 mL) and boron tribromide (BBr_3, 2.67 mL, 28.26 mmol, 6 eq.). HAmIm **1** (2.00 g, 4.71 mmol, 1 eq.) was dissolved in hexanes (50 mL) and added dropwise to the above mixture. An intense stream of nitrogen was applied during the addition to remove as much as possible hydrogen bromide formed in the reaction. A white precipitate was formed immediately upon addition. After 15 min the mixture was cannula-filtrated. Diethyl ether (50 mL) was added to the solid part. The suspension was stirred for 1 h and then again cannula-filtrated, which gave compound **7-BBr4** (3.50 g, 4.19 mmol, 89 %) sufficiently pure for further reactions. Crystals suitable for X-ray crystallography (Figure S8) and elemental analysis were obtained by layering pentane with a solution of **7-BBr4** in dichloromethane.

^{1}H NMR (CD_2Cl_2, 400 MHz, 293 K): δ = 1.16 (6 H, d, $^{3}J_{HH}$ = 6.9 Hz, CH_3 in *i*Pr), 1.22 (6 H, d, $^{3}J_{HH}$ = 6.9 Hz, CH_3 in *i*Pr), 1.56 (6 H, d, $^{3}J_{HH}$ = 7.0 Hz, CH_3 in *i*Pr), 1.66 (6 H, d, $^{3}J_{HH}$ = 7.0 Hz, CH_3 in *i*Pr), 1.73 (3 H, q, $^{5}J_{HH}$ = 0.9 Hz, C*C*H_3), 1.96 (3 H, q, $^{5}J_{HH}$ = 1.0 Hz, C*C*H_3), 2.47 (6 H, s, CH_3, imidazoline backbone), 2.68 (2 H, sept, $^{3}J_{HH}$ = 6.9 Hz, C*H*Me_2 in Dipp), (2 H, sept, $^{3}J_{HH}$ = 7.1 Hz, C*H*Me_2 in imidazoline), 7.28 (2 H, d, $^{3}J_{HH}$ = 7.8 Hz, aryl-CH), 7.28 (1 H, t, $^{3}J_{HH}$ = 7.8 Hz, aryl-CH).

^{13}C{^{1}H} NMR (CD_2Cl_2, 100 MHz, 293 K): δ = 10.7 (C*C*H_3, imidazoline backbone), 10.8 (C*C*H_3), 11.4 (C*C*H_3), 21.4 (*C*H_3 in *i*Pr), 21.7 (*C*H_3 in *i*Pr), 23.5 (*C*H_3 in *i*Pr), 24.5 (*C*H_3 in *i*Pr), 29.1 (*C*HMe_2 in Dipp), 52.1 (*C*HMe_2 in imidazoline), 115.5 (aryl-C), 119.5 (aryl-C), 124.4 (aryl-CH), 127.1 (aryl-C), 129.4 (aryl-CH), 133.0 (aryl-C), 135.2 (aryl-C), 146.3 (N_3*C*).

^{11}B{^{1}H} NMR (CD_2Cl_2, 128 MHz, 293 K): δ_B = −24.2 (BBr_4^-, $\omega_{1/2}$ = 3 Hz), 21.4 (N_2BBr, $\omega_{1/2}$ = 152 Hz).

Elemental analysis. Calculated for $C_{27}H_{43}BBr_5N_4$: C 38.39, H 5.13, N 6.63. Found: C 37.98, H 4.97, N 6.59.

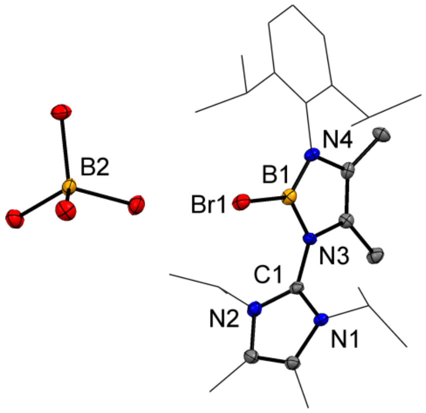

Figure S8. Molecular structure of compound **7-BBr4**. Thermal ellipsoids are presented at the 50 % level of probability. Hydrogen atoms are omitted for clarity. The asymmetric unit in the space group $P2_1/c$ contains one ion pair of **7-BBr4**. Bond distances and bond angles are reported in Å or degree (°), respectively. Br1−B1 1.913(6), N3−C1 1.379(6), N3−B1 1.429(7), N4−B1 1.399(7), N4−B1−N3 106.0(4), N4−B1−Br1 130.0(4), N3−B1−Br1 123.9(4).

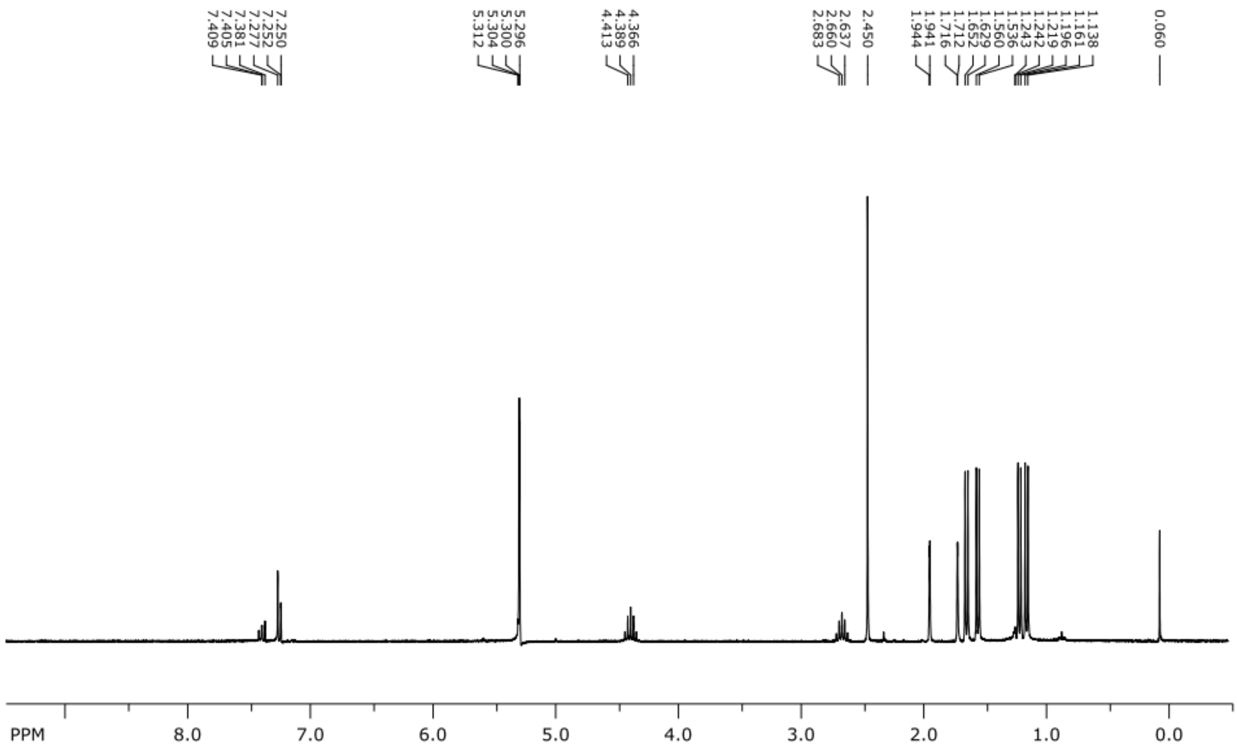

Figure S9. ^{1}H NMR (CD_2Cl_2, 400 MHz, 293 K) of compound **7-BBr4**.

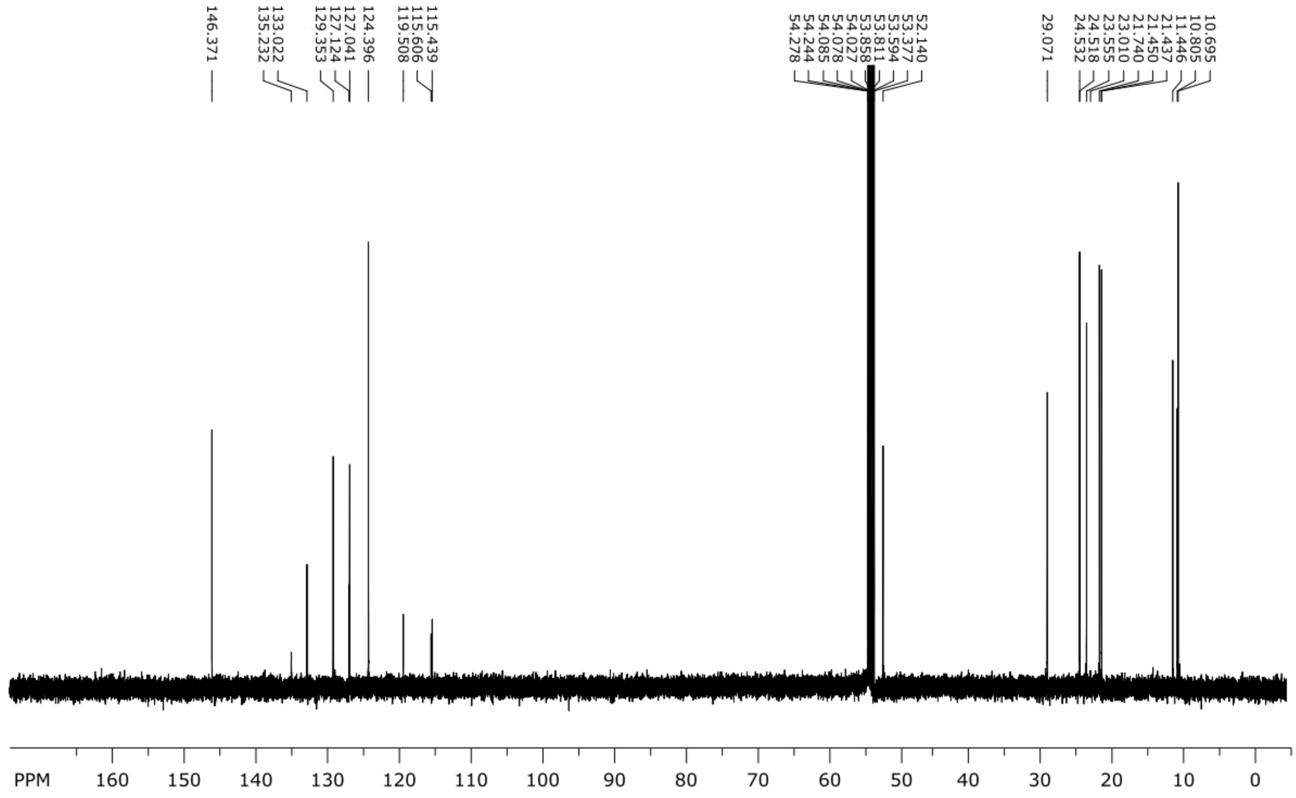

Figure S10. $^{13}C\{^{1}H\}$ NMR (CD_2Cl_2, 100 MHz, 293 K) of compound **7-BBr4**.

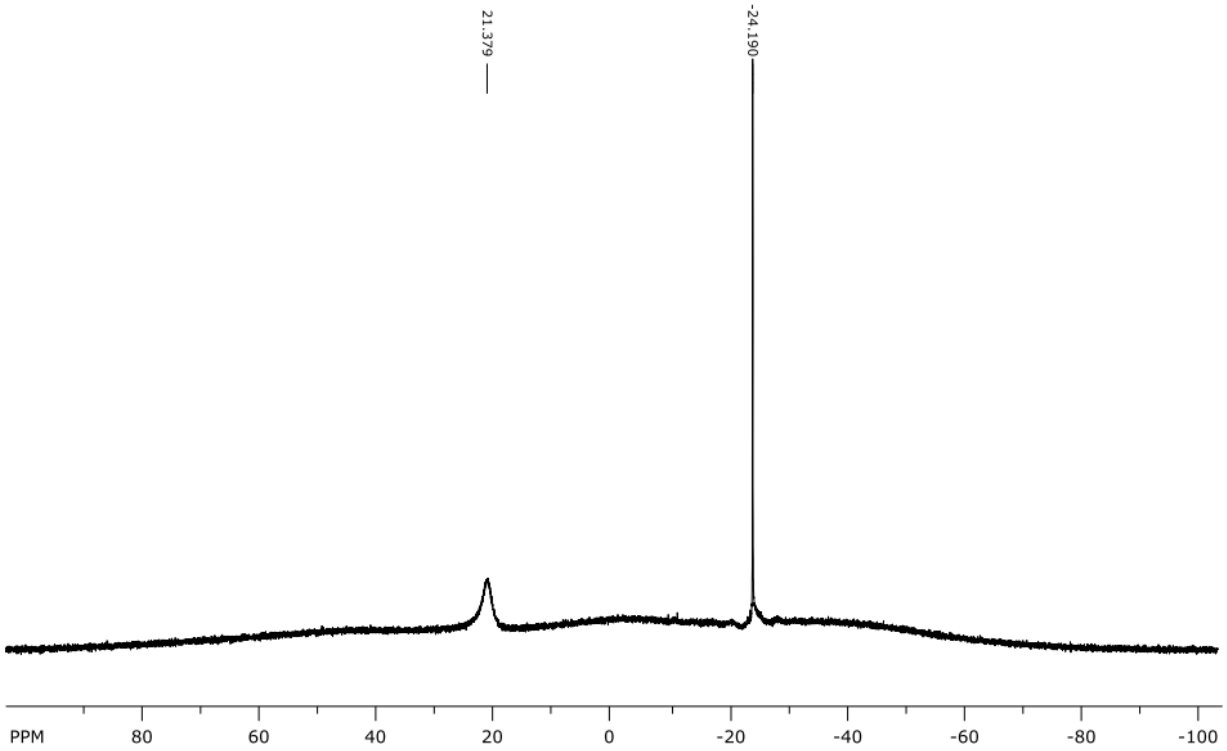

Figure S11. $^{11}B\{^1H\}$ NMR (CD_2Cl_2, 128 MHz, 293 K) of compound **7-BBr$_4$**.

*1.3.2. Synthesis of Compound **7-Br**.*

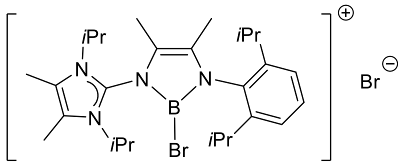

7-Br

A side arm flask (the neck should be large in diameter) was charged with dichloromethane (100 mL) and **7-BBr₄** (3.00 g, 4.14 mmol, 1 eq.). Silver tetrafluoroborate ($AgBF_4$, 1.60 g, 8.28 mmol, 2 eq.) was added in one portion with the application of an intense stream of nitrogen. After 5 min the mixture was cannula-filtrated. The solvent of the filtrate was removed *in vacuo*. Diethyl ether (50 mL) was added to the hone-like residue and stirred for 3 h. The solution was decanted. The solid was dried *in vacuo*, which gave compound **7-Br** (1.90 g, 3.28 mmol, 80 %) sufficiently pure for further reactions. Crystals suitable for X-ray crystallography (Figure S12) and elemental analysis were obtained by layering diethyl ether with a solution of **7-BBr** in dichloromethane and were found to have the composition (**7-Br**)$_2$ · Et_2O.

^{1}H NMR (CD_2Cl_2, 400 MHz, 293 K): δ = 1.13 (6 H, d, $^3J_{HH}$ = 6.9 Hz, CH_3 in *i*Pr), 1.20 (6 H, d, $^3J_{HH}$= 6.9 Hz, CH_3 in *i*Pr), 1.54 (6 H, d, $^3J_{HH}$ = 7.0 Hz, CH_3 in *i*Pr), 1.67 (6 H, d, $^3J_{HH}$ = 7.0 Hz, CH_3 in *i*Pr), 1.73 (3 H, q, $^5J_{HH}$ = 1.0 Hz, C*C*H$_3$), 1.95 (3 H, q, $^5J_{HH}$ = 1.0 Hz, C*C*H$_3$), 2.49 (6 H, s, CH_3, imidazoline backbone), 2.67 (2 H, sept, $^3J_{HH}$ = 7.0 Hz, C*H*Me$_2$ in Dipp), 4.43 (2 H, sept, $^3J_{HH}$ = 7.1 Hz, C*H*Me$_2$ in imidazoline), 7.26 (2 H, d, $^3J_{HH}$ = 7.7 Hz, aryl-CH), 7.40 (1 H, t, $^3J_{HH}$= 7.7 Hz, aryl-CH).

^{13}C{^{1}H} NMR (CD_2Cl_2, 100 MHz, 293 K): δ = 10.6 (C*C*H$_3$, imidazoline backbone), 10.7 (C*C*H$_3$), 11.3 (C*C*H$_3$), 21.3 (*C*H$_3$ in *i*Pr), 21.7 (*C*H$_3$ in *i*Pr), 23.4 (*C*H$_3$ in *i*Pr), 24.4 (*C*H$_3$ in *i*Pr), 29.0 (*C*HMe$_2$ in Dipp), 52.1 (*C*HMe$_2$ in imidazoline), 119.5, (*C*CH$_3$, imidazoline backbone), 124.3 (aryl-CH), 126.9 (*C*CH$_3$), 127.0 (*C*CH$_3$), 129.2 (aryl-CH), 133.0 (aryl-C), 135.0 (aryl-C), 146.3 (N$_3$*C*).

^{11}B{^{1}H} NMR (CD_2Cl_2, 128 MHz, 293 K): δ_B = 21.2 ($\omega_{1/2}$ = 170 Hz).

Elemental analysis was performed with samples obtained from the crystallization procedure.
Calculated for $C_{58}H_{96}B_2Br_4N_8O$ ≡ (**7-Br**)$_2$ · Et_2O: C 55.17, H 7.66, N 8.87. Found: C 55.03, H 7.45, N 9.02.

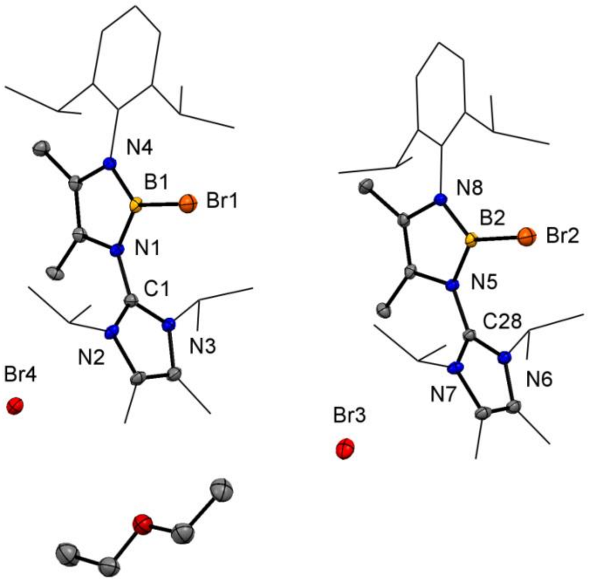

Figure S12. Molecular structures of compound **7-Br**. Thermal ellipsoids are presented at the 50 % level of probability. Hydrogen atoms are omitted for clarity. The full asymmetric unit in the space group *P*32 is displayed and contains two crystallographically independent ion pairs of **7-Br** and one molecule of diethyl ether. Bond distances and bond angles are reported in Å or degree (°), respectively. First ion pair: Br1−B1 1.913(3), N1−C1 1.384(4), N1−B1 1.429(4), N4−B1 1.402(4), N4−B1−N1 106.0(2), N4−B1−Br1 128.7(2), N1−B1−Br1 125.4(2). Second ion pair: Br2−B2 1.905(3), N5−C28 1.388(4), N5−B2 1.428(4), N8−B2 1.408(4), N8−B2−N5, 105.7(2), N8−B2−Br2 128.3(2), N5−B2−Br2 126.0(2).

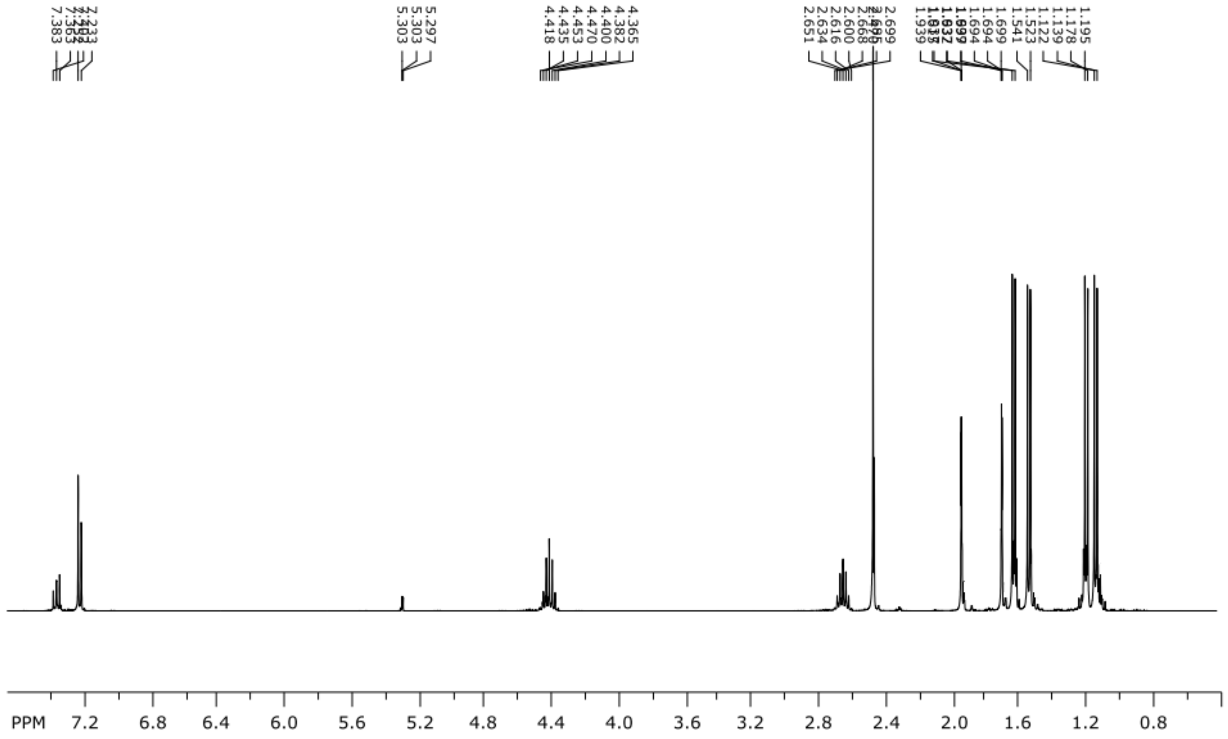

Figure S13. ^{1}H NMR (CD_2Cl_2, 400 MHz, 293 K) of compound **7-Br**.

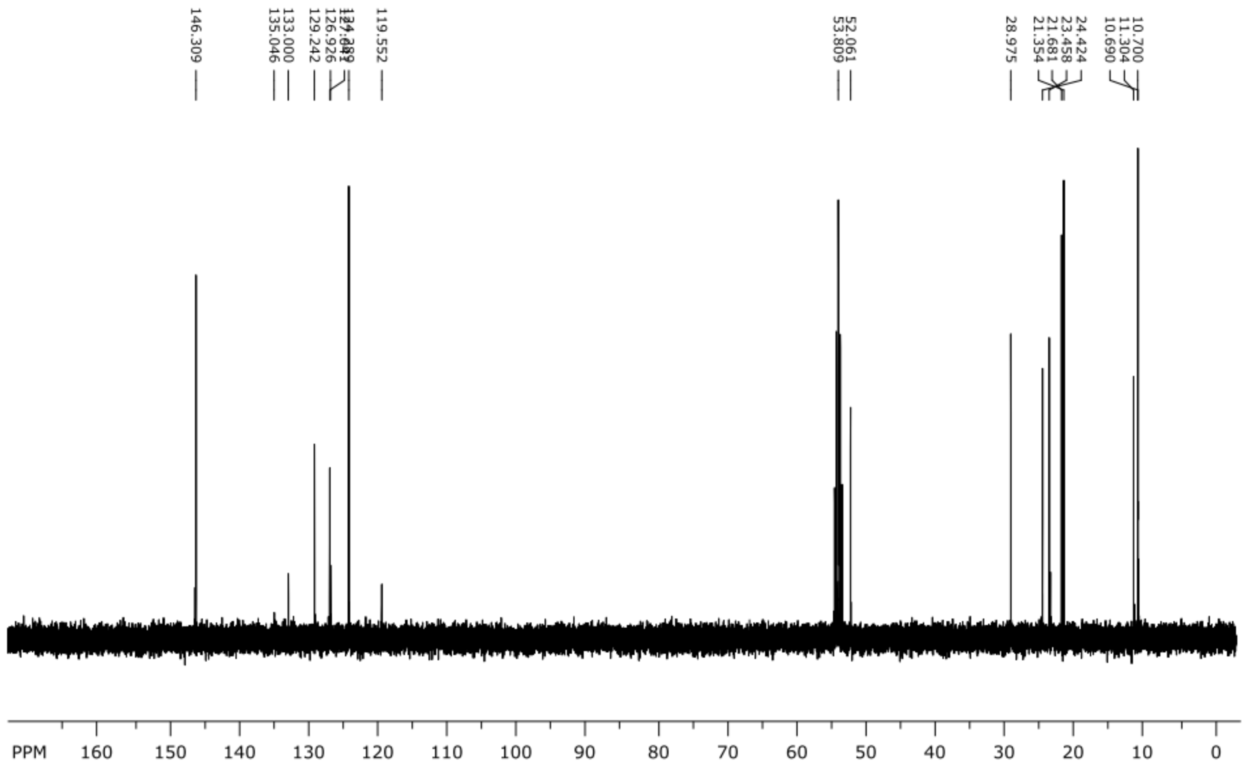

Figure S14. $^{13}C\{^{1}H\}$ NMR (CD_2Cl_2, 100 MHz, 293 K) of compound **7-Br**.

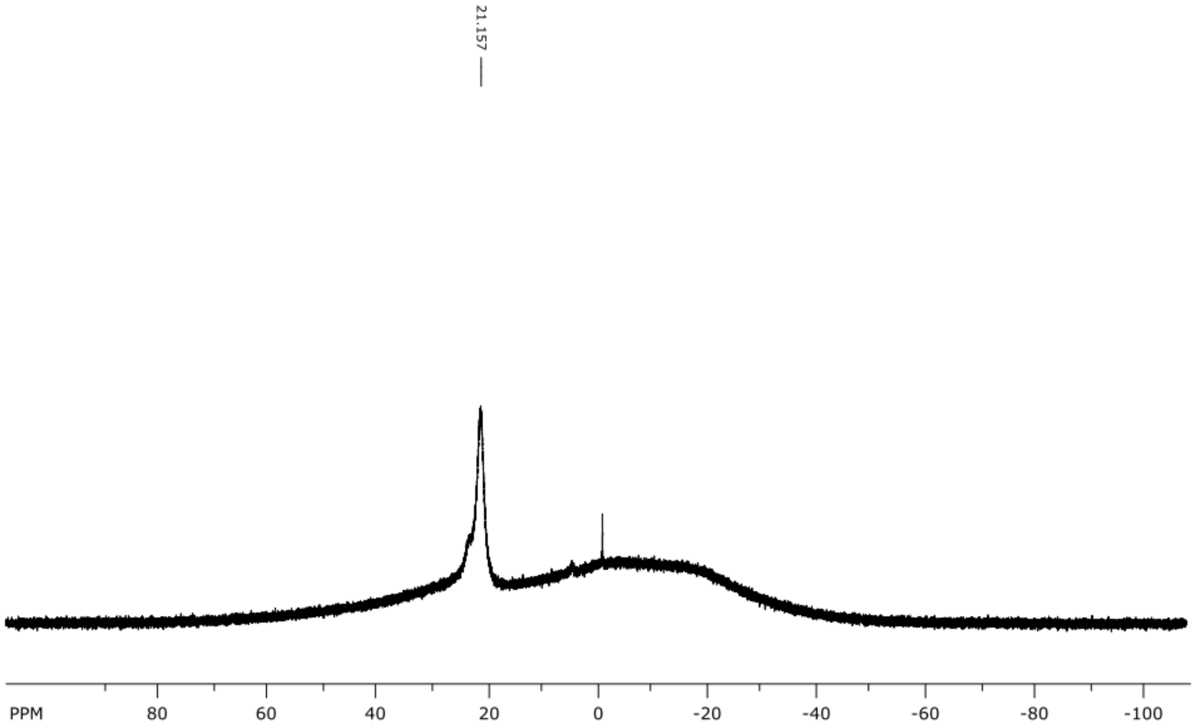

Figure S15. $^{11}B\{^{1}H\}$ NMR (CD_2Cl_2, 128 MHz, 293 K) of compound **7-Br**.
.

*1.4. Synthesis of Compounds **8** and **9**.*

*1.4.1. Synthesis of Compound **8**.*

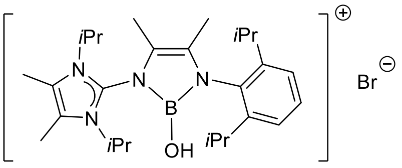

8

Compound **7-Br** (1.00 g, 1.68 mmol, 1 eq.) dissolved in dichloromethane (60 mL). A mixture of H_2O (29 mg, 1.65 mmol, 1 eq.) and NEt_3 (330 mg, 3.30 mmol, 2 eq.) in dichloromethane (40 mL) was added dropwise under nitrogen flow. The reaction stirred for 2 h. Calcium hydride (CaH_2, 140 mg, 2 eq.) was added and the suspension was stirred for 1 h. Canula filtration and removal of the solvent *in vacuo* gave compound **8** (623 mg, 1.176 mmol, 70 %), sufficiently pure for further manipulation. Analytically pure samples for elemental analysis and crystals suitable for X-ray crystallography (Figure S16) were obtained by layering diethyl ether with a solution of compound **3** in dichloromethane.

^{1}H NMR ($CDCl_3$, 400 MHz, 293 K): δ = 1.04 (6 H, d, $^3J_{HH}$ = 6.9 Hz, CH_3 in *i*Pr), 1.10 (6 H, d, $^3J_{HH}$ = 7.0 Hz, CH_3 in *i*Pr), 1.47 (3 H, q, $^5J_{HH}$ = 1.0 Hz, C*C*H_3), 1.49 (12 H, d, $^3J_{HH}$ = 7.1 Hz, CH_3 in *i*Pr), 1.67 (3 H, q, $^5J_{HH}$ = 1.0 Hz, C*C*H_3), 2.20 (6 H, s, CH_3, imidazoline backbone), 2.78 (2 H, sept, $^3J_{HH}$ = 7.0 Hz, C*H*Me_2 in Dipp), 4.34 (2 H, sept, $^3J_{HH}$ = 7.1 Hz, C*H*Me_2 in imidazoline), 7.05−7.21 (3 H, m, Aryl-CH), 7.37 (1 H, s, OH).

^{13}C{^{1}H} NMR ($CDCl_3$, 100 MHz, 293 K): δ = 10.1 (C*C*H_3, imidazoline backbone), 10.4 (C*C*H_3), 10.6 (C*C*H_3), 21.4 (*C*H_3 in *i*Pr), 21.5 (*C*H_3 in *i*Pr), 22.9 (*C*H_3 in *i*Pr), 24.6 (*C*H_3 in *i*Pr), 28.5 (*C*HMe_2 in Dipp), 50.7 (*C*HMe_2 in imidazoline), 112.3 (*C*CH_3), 122.4 (*C*CH_3), 123.1 (aryl-CH), 125.4 (*C*CH_3, imidazoline backbone), 127.5 (aryl-CH), 128.3 (aryl-C), 134.2 (aryl-C), 136.4 (aryl-C), 146.9 (N_3*C*).

^{11}B{^{1}H} NMR ($CDCl_3$, 128 MHz, 293 K): δ = 22.9 ($\omega_{1/2}$ = 520 Hz).

Elemental analysis was performed with samples obtained from the crystallization procedure.
Calculated for $C_{27}H_{44}BBrN_4O$: C 61.03, H 8.35, N 10.54. Found: C 61.25, H 8.16, N 10.59.

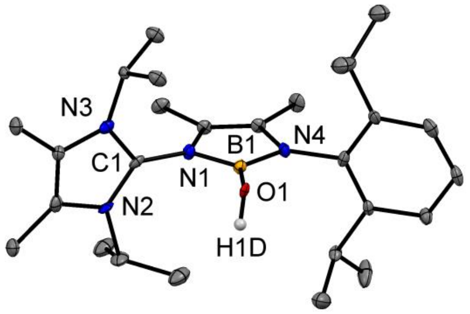

Figure S16. Molecular structure of compound **8**. Thermal ellipsoids are presented at the 50 % level of probability. Carbon bound hydrogen atoms and the anion (Br^-) are omitted for clarity. The asymmetric unit contains two crystallographically independent molecules, only one of which is depicted. Bond distances and bond angles are reported in Å or degree (°), respectively. O1−B1 1.353(9), N1−B(1) 1.422(10), N4−B1 1.421(9), N1−C1 1.356(9), O1−B1−N4 127.1(7), O1−B1−N1 125.8(7), N4−B1−N1 107.1(6).

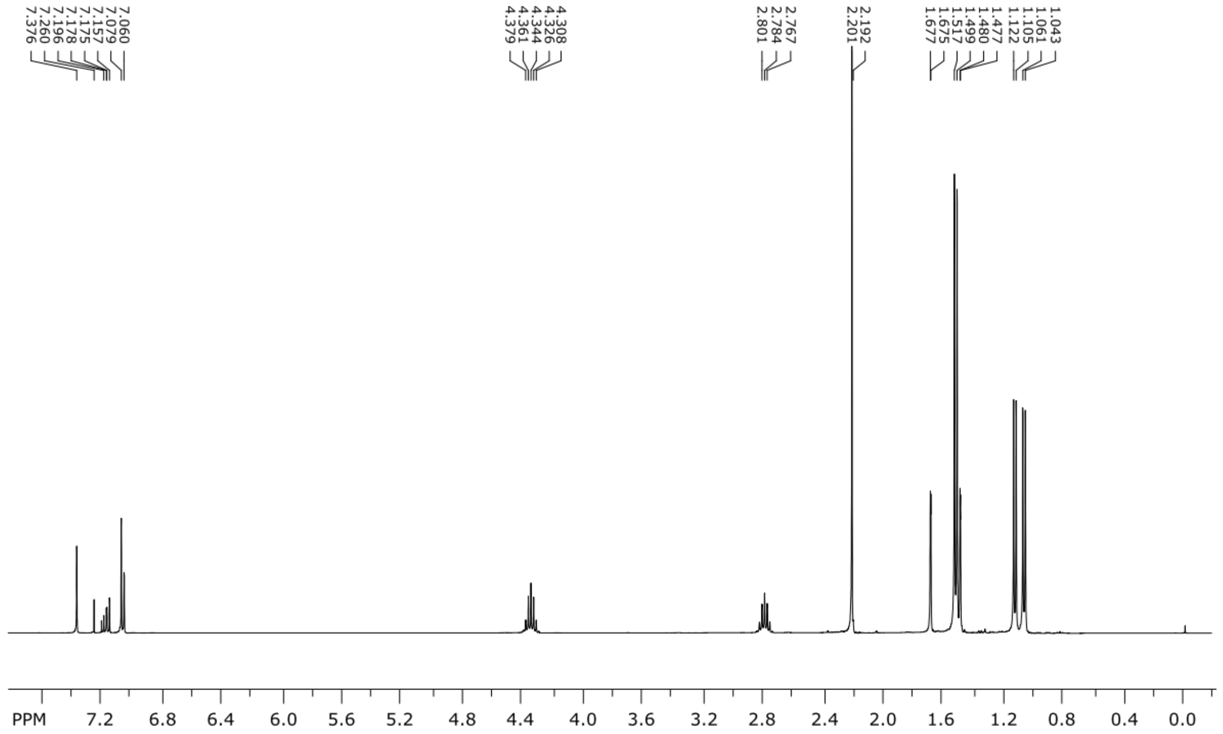

Figure S17. ^{1}H NMR ($CDCl_3$, 400 MHz, 293 K) of compound **8**.

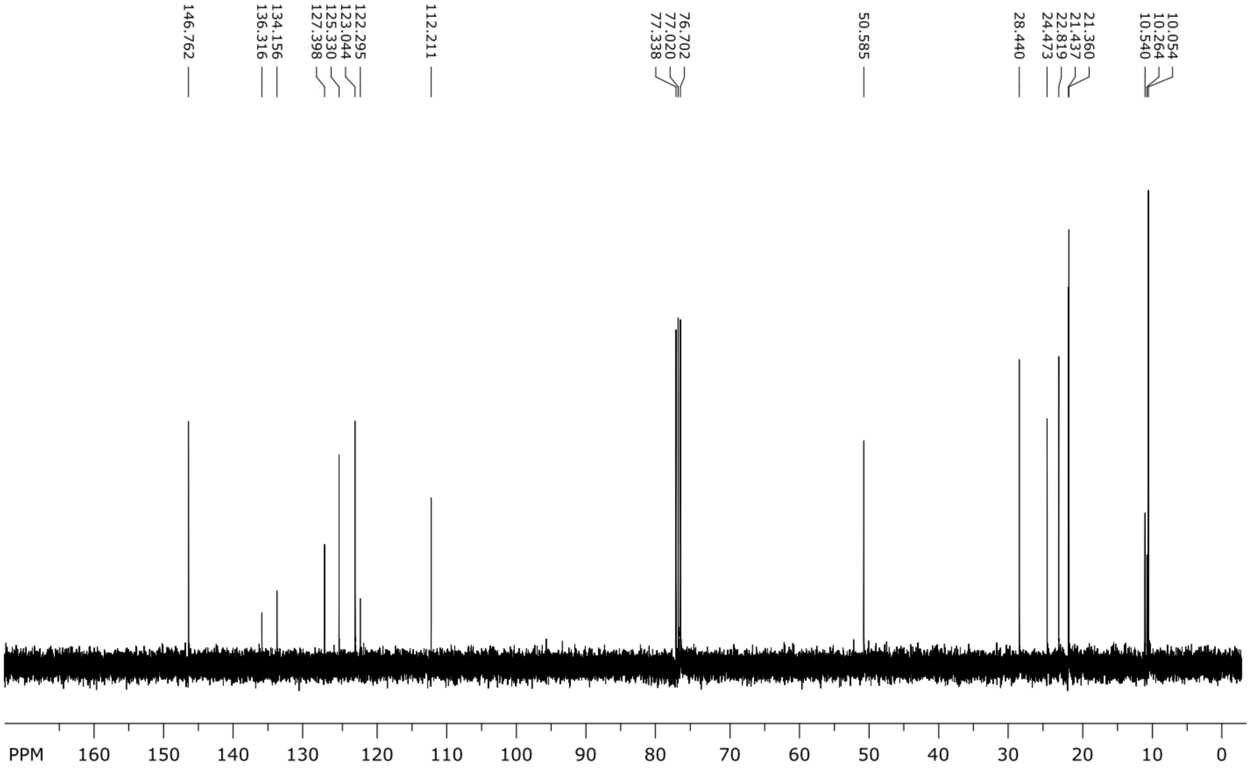

Figure S18. $^{13}C\{^{1}H\}$ NMR ($CDCl_3$, 100 MHz, 293 K) of compound **8**.

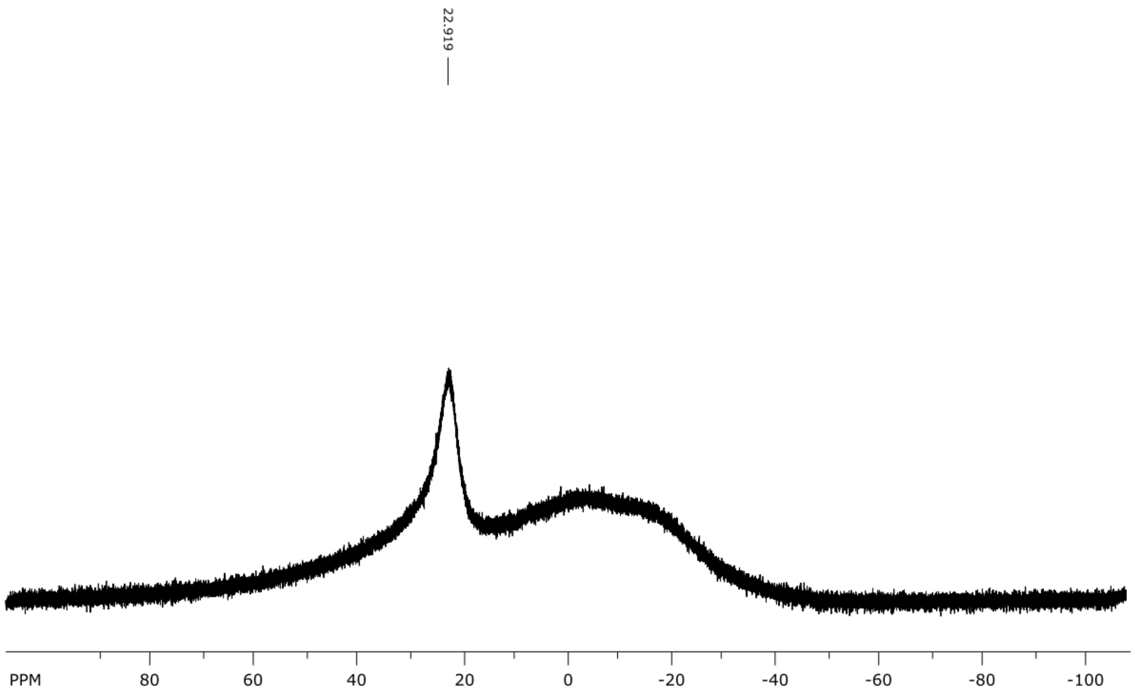

Figure S19. $^{11}B\{^{1}H\}$ NMR ($CDCl_3$, 128 MHz, 293 K) of compound **8**.

*1.4.2. Synthesis of Compound **9**.*

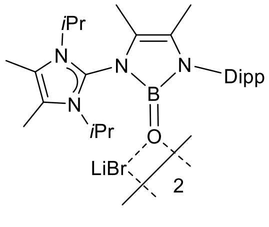

9

Compound **3** (200 mg, 0.37 mmol, 1 eq.) was dissolved in THF (10 mL) and a solution of lithium hexamethyl disilylamide (LiHMDS, 472 µL, 1 M in THF, 2 eq.) was added with stirring. After 1 h a small amount of a precipitate was formed, which was removed by cannula-filtration. Layering of *n*-pentane over the THF crude solution afforded crystals suitable for X-ray crystallography, which showed the intercalation of an undefined amount of THF in the void volume of the lattice, and was thus treated with the SQUEEZE-tool in OLEX^2, see Figure S20.

The filtrate was further reduced to dryness *in vacuo*. The final compound **9** can only hardly be redissolved in THF, but readily dissolves in dichloromethane. Samples for NMR characterization and elemental analysis were obtained by dissolution of the material in dichloromethane (5 mL) and precipitation of product **9** upon addition of *n*-pentane (10 mL), which afforded **9** as a white powder (120 mg, 0.22 mmol, 60 %).

^{1}H NMR (CD_2Cl_2, 400 MHz, 293 K): δ = 0.87 (6 H, d, $^3J_{HH}$ = 6.9 Hz, CH_3 in *i*Pr), 1.10 (6 H, d, $^3J_{HH}$ = 7.0 Hz, CH_3 in *i*Pr), 1.34 (6 H, d, $^3J_{HH}$ = 7.0 Hz, CH_3 in *i*Pr), 1.44 (3 H, s, CCH_3), 1.45 (6 H, d, $^3J_{HH}$ = 7.0 Hz, CH_3 in *i*Pr), 1.65 (3 H, s, CCH_3), 2.24 (6 H, s, CH_3, imidazoline backbone), 2.95 (2 H, sept, $^3J_{HH}$ = 7.0 Hz, C*H*Me$_2$ in Dipp), 4.53 (2 H, sept, $^3J_{HH}$ = 7.3 Hz, C*H*Me$_2$ in imidazoline), 7.08 (2 H, d, $^3J_{HH}$ = 7.7 Hz, aryl-CH), 7.26 (1 H, t, $^3J_{HH}$ = 7.6 Hz, aryl-CH).

^{13}C{^{1}H} NMR (CD_2Cl_2, 100 MHz, 293 K): δ = 10.4 (C*C*H$_3$, imidazoline backbone), 10.8 (C*C*H$_3$), 11.0 (C*C*H$_3$), 21.5 (*C*H$_3$ in *i*Pr), 21.9 (*C*H$_3$ in *i*Pr), 23.3 (*C*H$_3$ in *i*Pr), 25.2 (*C*H$_3$ in *i*Pr), 28.3(*C*HMe$_2$ in Dipp), 50.2 (*C*HMe$_2$ in imidazoline), 111.4 (*C*CH$_3$), 122.3 (*C*CH$_3$), 123.9 (aryl-CH), 124.6 (*C*CH$_3$, imidazoline backbone), 127.1 (aryl-CH), 137.0 (aryl-C), 139.8 (aryl-C), 147.8 (N$_3$*C*).

^{11}B{^{1}H} NMR (CD_2Cl_2, 128 MHz, 293 K): δ = 21.6 ($\omega_{1/2}$ = 700 Hz).

Elemental analysis. Calculated for $C_{27}H_{43}BBrLiN_4O$: C 60.35, H 8.07, N 10.43. Found: C 60.56, H 8.24, N 10.27.

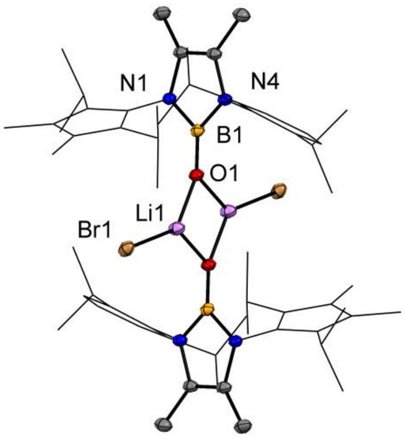

Figure S20. Molecular structure of compound **9**. The dimer is located on a center of inversion. Thermal ellipsoids are presented at the 50 % level of probability. Hydrogen atoms are omitted for clarity. Bond distances and bond angles are reported in Å or degree (°), respectively. O1−B1 1.3040(17), N1−B1 1.489(2) N4−B1 1.4523(18), Br1−Li1 2.402(3), O1−Li 1.804(3), O1−B1-N4 131.45(13), O1−B1−N1 126.06(13), N4−B1−N1 102.49(11).

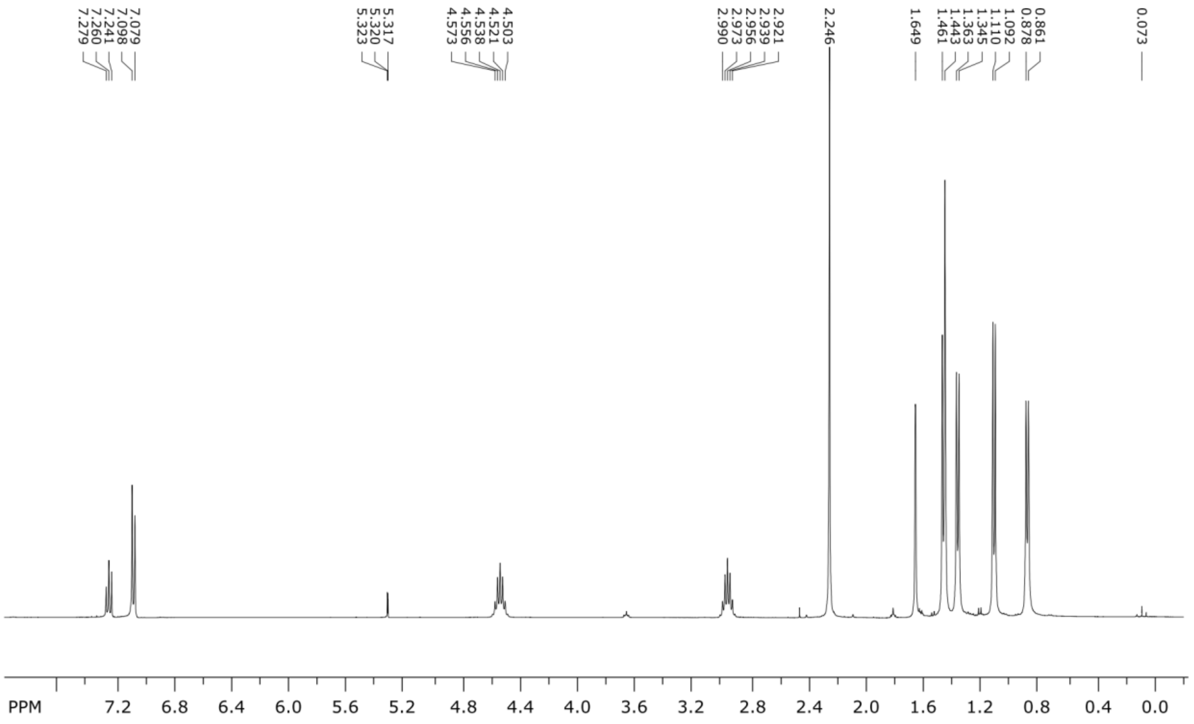

Figure S21. ^{1}H NMR (CD_2Cl_2, 400 MHz, 293 K) of compound **9**.

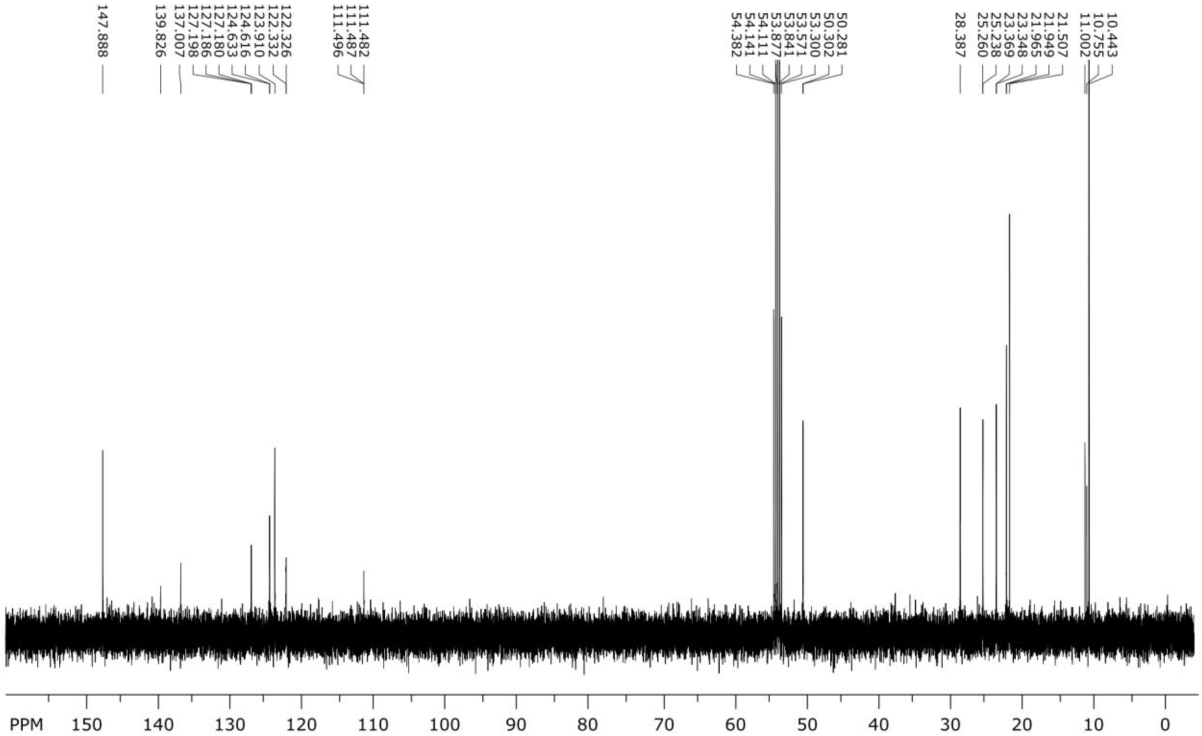

Figure S22. $^{13}C\{^{1}H\}$ NMR (CD_2Cl_2, 100 MHz, 293 K) of compound **9**.

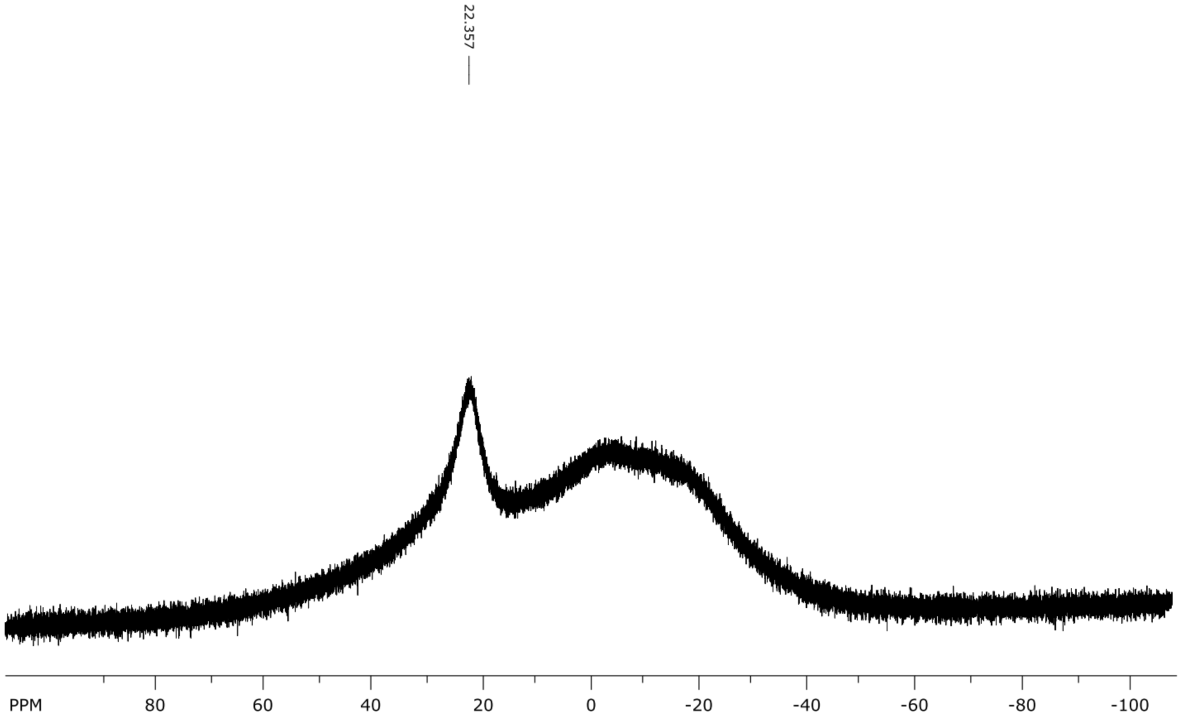

Figure S23. $^{11}B\{^{1}H\}$ NMR (CD_2Cl_2, 128 MHz, 293 K) of compound **9**.

*1.5. Synthesis of Compound **10**.*

Compound **8** (350 mg, 0.66 mmol, 1 eq.) was dissolved in THF (10 mL).

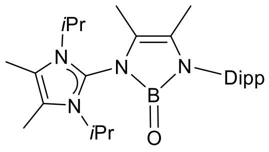

10

Potassium hexamethyl disilylamide (KHMDS, 131 mg, 1 eq.) and 2.2.2-cryptand ($C_{18}N_2H_{36}O_6$, 248 mg, 1 eq.) were dissolved in THF (5 mL) at room temperature. This solution was added to the solution aforementioned at ambient temperature and stirring was continued for 30 min with formation of a white precipitate, which was removed by cannula-filtration. The solvent was removed to dryness.

The solid residue was treated following the aforementioned procedure with two additional cycles. The final solid was then extracted with Et_2O (15 mL). The extract was concentrated to 2-3 mL and subjected to layering with pentane, which afforded colorless crystals of compound **10** (163 mg, 0.36 mmol, 55 %).

^{1}H NMR (THF-d_8, 400 MHz, 293 K): δ = 1.16 (6 H, d, $^3J_{HH}$ = 7.0 Hz, CH_3 in *i*Pr), 1.22 (6 H, d, $^3J_{HH}$ = 6.8 Hz, CH_3 in *i*Pr), 1.46 (3 H, s, CCH_3), 1.52 (6 H, d, $^3J_{HH}$ = 7.2 Hz, CH_3 in *i*Pr), 1.57 (6 H, d, $^3J_{HH}$ = 7.0 Hz, CH_3 in *i*Pr), 1.75 (3 H, s, CCH_3), 2.28 (6 H, s, CH_3, imidazoline backbone), 3.24 (2 H, sept, $^3J_{HH}$ = 6.9 Hz, C*H*Me_2 in Dipp), 4.71 (2 H, sept, $^3J_{HH}$= 7.1 Hz, C*H*Me_2 in imidazoline), 7.03−7.10 (3 H, m, aryl-CH), 7.29 (6 H, s, 1 × C_6H_6).

^{13}C{^{1}H} NMR (THF-d_8, 100 MHz, 293 K): δ = 10.4 (CCH_3, imidazoline backbone), 10.7 (CCH_3), 10.9 (CCH_3), 21.3 (CH_3 in *i*Pr), 21.8 (CH_3 in *i*Pr), 23.1 (CH_3 in *i*Pr), 25.2 (CH_3 in *i*Pr), 29.0 ($CHMe_2$ in Dipp), 51.2 ($CHMe_2$ in imidazoline), 111.9, 121.6 (aryl-CH), 123.0, 123.1, 124.8, 126.7 (aryl-CH), 140.6, 148.0 (N_3C).

^{11}B{^{1}H} NMR (CD_2Cl_2, 128 MHz, 293 K): δ = 21.4 ($\omega_{1/2}$ = 600 Hz).

IR: $\bar{\nu}$ = 1667 cm^{-1} (B=O), see Figure S27.

Elemental analysis. Calculated for $C_{27}H_{43}BN_4O$: C 71.99, H 9.62, N 12.44. Found: C 72.05, H 9.85, N 12.21.

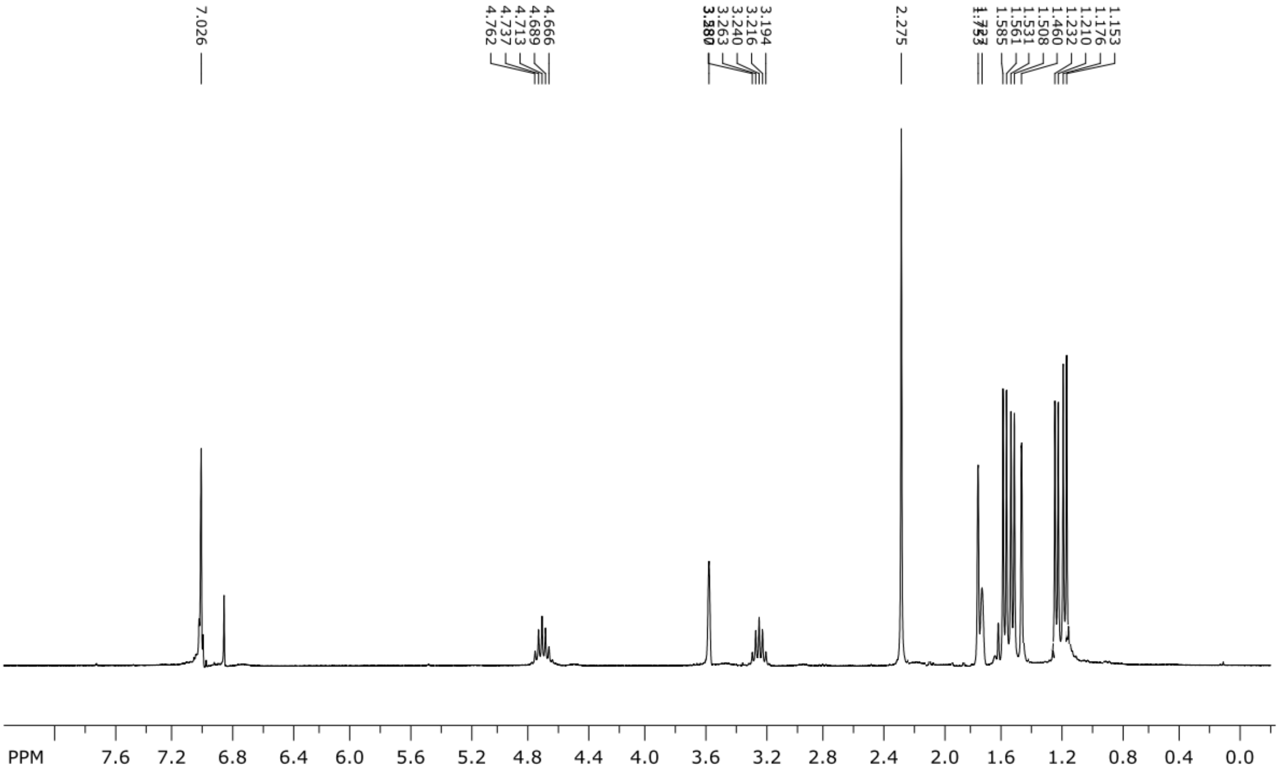

Figure S24. ^{1}H NMR (CD_2Cl_2, 400 MHz, 293 K) of compound **10**.

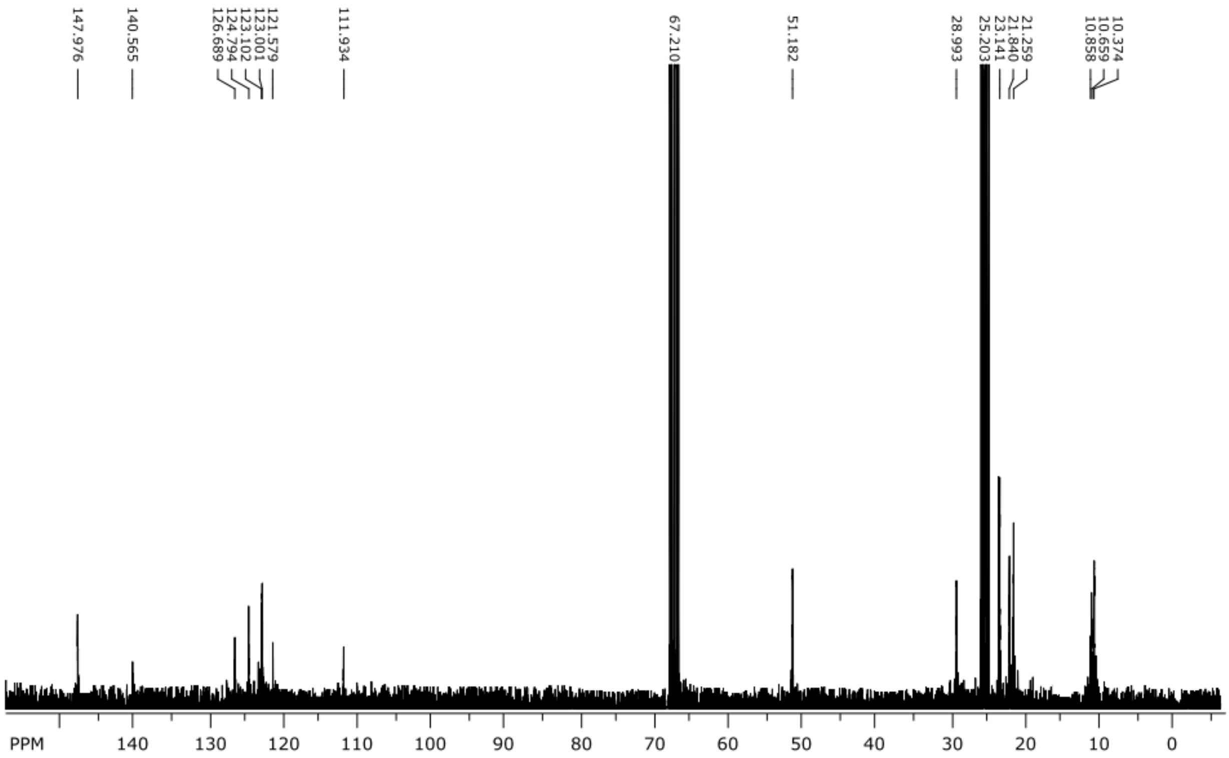

Figure S25. $^{13}C\{^{1}H\}$ NMR (CD_2Cl_2, 100 MHz, 293 K) of compound **10**.

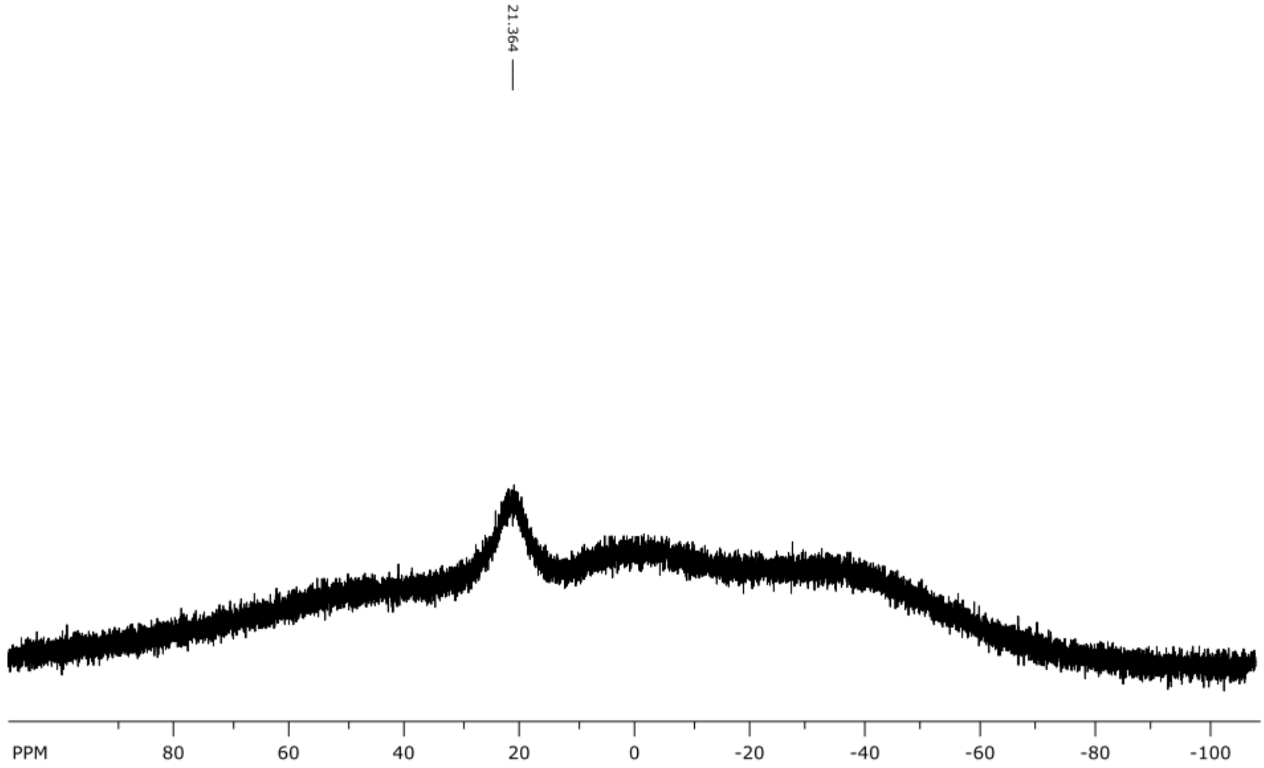

Figure S26. $^{11}B\{^{1}H\}$ NMR (CD_2Cl_2, 128 MHz, 293 K) of compound **10**.

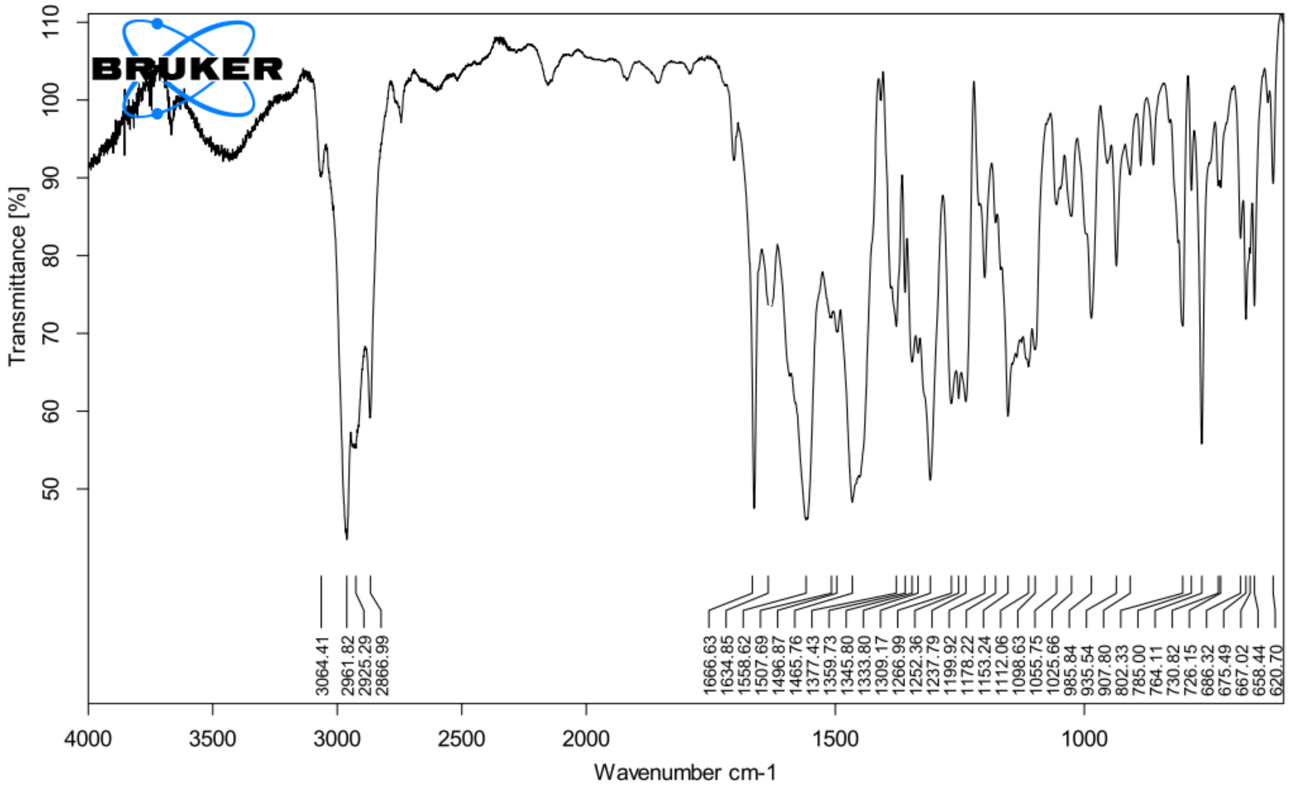

Figure S27. FT-IR spectrum (KBr disc) of compound **10**.

*1.6. Synthesis of Compound **11**.*

Compound **7-Br** (200 mg, 0.33 mmol, 1 eq.) was suspended in 1,2-dimethoxyethane (10 mL) and Li_2S (30 mg, 1.34 mmol, 2 eq.) was added. The suspension was heated to 50 °C overnight. Volatile components were removed *in vacuo*, and the product **11** was extracted with benzene (15 mL) in the form of its adduct with lithium bromide. The addition of 12-crown-4 ($C_8H_{16}O_4$, 5.8 mg, 0.33 mmol, 1 eq.) to the benzene solution and stirring for 30 min led to a precipitate of lithium bromide crown ether complex, which was removed by cannula-filtration. The filtrate was condensed to a volume of ca. 5 mL under reduced pressure. Slow solvent evaporation gave colorless crystals (also suitable for X-ray crystallography) of the composition **6** · C_6H_6 (107 mg, 0.196 mmol, 60 %).

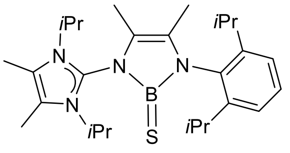

11

^{1}H NMR (THF-d$_8$, 400 MHz, 293 K): δ = 1.12 (6 H, d, $^3J_{HH}$ = 7.0 Hz, CH_3 in *i*Pr), 1.28 (6 H, d, $^3J_{HH}$ = 6.8 Hz, CH_3 in *i*Pr), 1.51 (3 H, d, $^3J_{HH}$ = 1.0 Hz, CCH_3), 1.55 (6H, d, $^3J_{HH}$ = 7.2 Hz, CH_3 in *i*Pr), 1.67 (6 H, d, $^3J_{HH}$ = 7.0 Hz, CH_3 in *i*Pr),1.79 (3 H, d, $^3J_{HH}$= 1.0 Hz, CCH_3), 2.36 (6 H, s, CH_3,imidazoline backbone), 3.18 (2 H, sept, $^3J_{HH}$ = 6.9 Hz, C*H*Me_2 in Dipp), 4.67 (2 H, sept, $^3J_{HH}$= 7.1 Hz, C*H*Me_2 in imidazoline), 7.03−7.11(3 H, m, aryl-CH), 7.29 (6 H, s, 1 × C_6H_6).

^{13}C{^{1}H} NMR (THF-d$_8$, 100 MHz, 293 K): δ = 10.0 (C*C*H_3, imidazoline backbone), 11.3 (C*C*H_3), 11.4 (C*C*H_3), 21.5 (*C*H_3 in *i*Pr), 21.7 (*C*H_3 in *i*Pr), 23.6 (*C*H_3 in *i*Pr), 25.5 (*C*H_3 in *i*Pr), 29.3 (*C*HMe_2 in Dipp), 51.3 (*C*HMe_2 in imidazoline), 113.4, 123.0 (aryl-CH), 124.3 (2 sorts of carbon atoms, overlap), 126.5 (aryl-CH), 129.0 (*C*$_6H_6$), 140.4 (aryl-C), 148.1 (N_3*C*).

^{11}B{^{1}H} NMR (THF-d$_8$, 128 MHz, 293 K): δ = 35.2 ($\omega_{1/2}$ = 320 Hz).

IR: see Figure S31.

Elemental analysis was performed with samples obtained from the crystallization procedure.
Calculated for $C_{33}H_{49}BN_4S$: ≡ **6** · C_6H_6: C 72.77, H 9.07, N 10.29. Found: C 72.34, H 8.89, N 10.11.

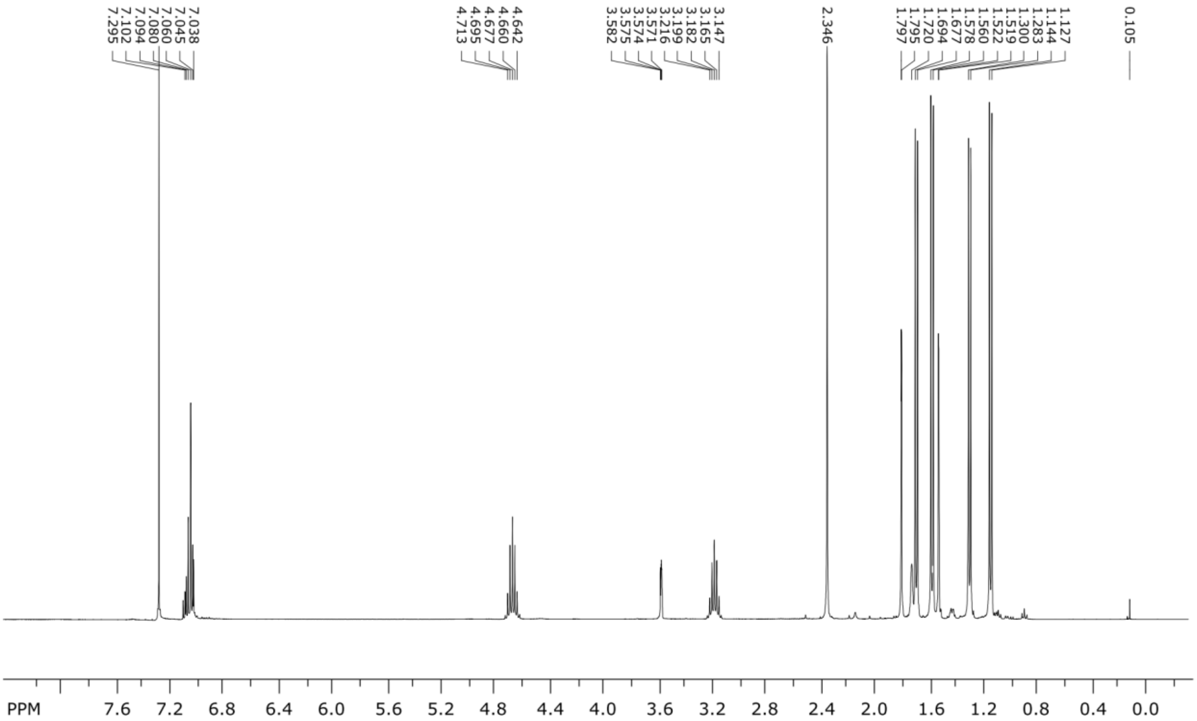

Figure S28. ^{1}H NMR (THF-d$_8$, 400 MHz, 293 K) of compound **11** · C_6H_6.

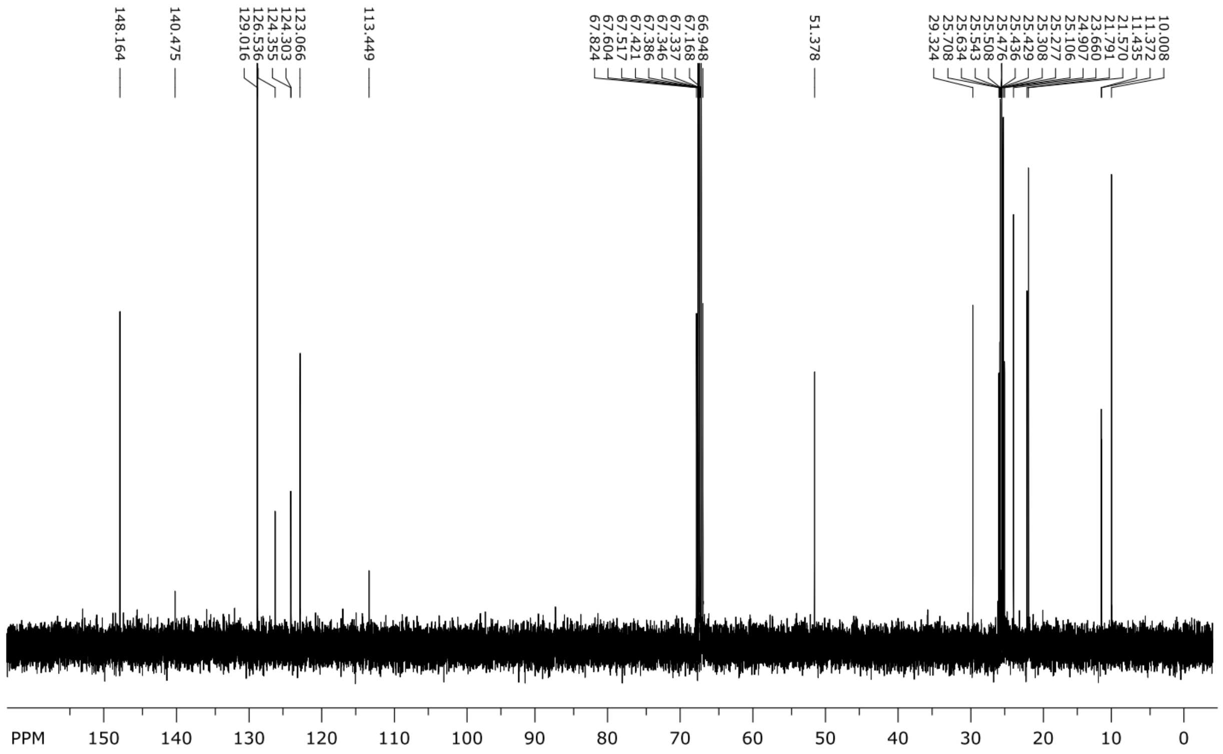

Figure S29. $^{13}C\{^{1}H\}$ NMR (THF-d$_8$, 100 MHz, 293 K) of compound **11** · C_6H_6.

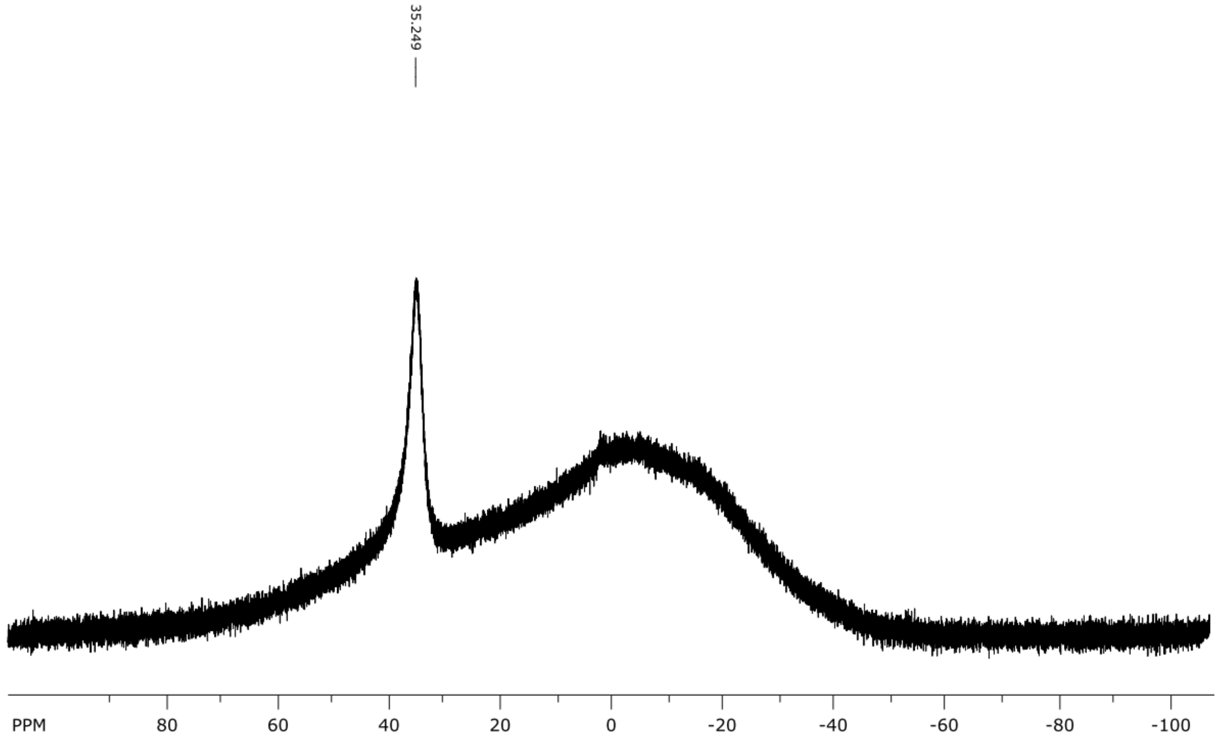

Figure S30. $^{11}B\{^{1}H\}$ NMR (THF-d$_8$, 128 MHz, 293 K) of compound **11** · C_6H_6.

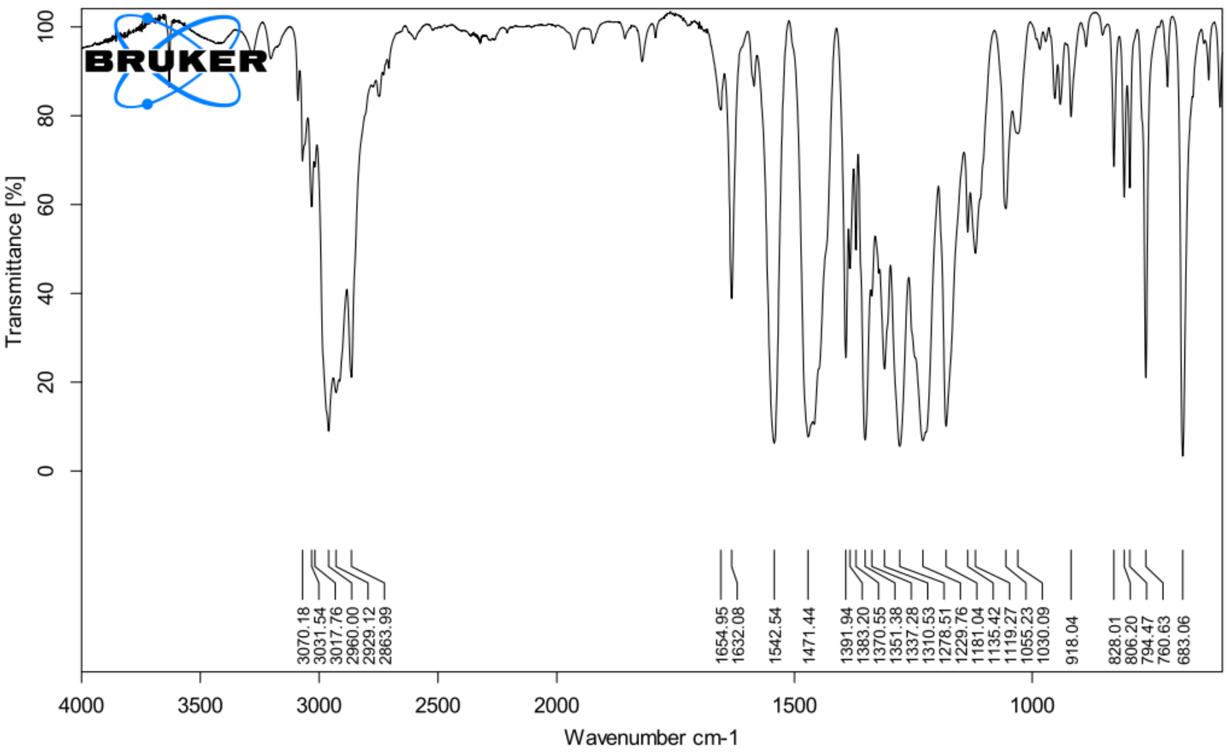

Figure S31. FT-IR spectrum (KBr disc) of compound **11** · C_6H_6.

1.7. Synthesis of Compound ***12****.*

Compound **7-Br** (200 mg, 0.33 mmol, 1 eq.) was suspended in 1,2-dimethoxyethane (10 mL) and Li_2Se (61 mg, 0.66 mmol, 2 eq.) was added. The suspension was heated to 50 °C overnight. Volatile components were removed *in vacuo*, and the product **12** was extracted with benzene (15 mL) in the form of its adduct with lithium bromide. The addition of 12-crown-4 ($C_8H_{16}O_4$, 5.8 mg, 0.33 mmol, 1 eq.) to the benzene solution and stirring for 30 min led to a precipitate of lithium bromide crown ether complex, which was removed by cannula-filtration. The filtrate was condensed to a volume of ca. 5 mL under reduced pressure. Slow solvent evaporation gave colorless crystals (also suitable for X-ray crystallography) of the composition **12** · 3 C_6H_6 (135 mg, 0.181 mmol, 55 %).

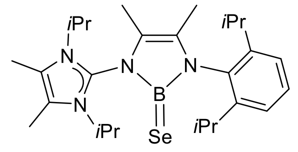

12

For the measurement of NMR and FT-IR spectra samples of compound **12** · 3 C_6H_6 were dried *in vacuo*, which led to the complete loss of the co-crystallized solvent C_6H_6.

^{1}H NMR (THF-d$_8$, 400 MHz, 293 K): δ = 1.10 (6 H, d, $^3J_{HH}$ = 7.0 Hz, CH_3 in *i*Pr), 1.27 (6 H, d, $^3J_{HH}$ = 6.8 Hz, CH_3 in *i*Pr), 1.51 (3 H, d, $^3J_{HH}$ = 1.0 Hz, CCH_3), 1.53 (6H, d, $^3J_{HH}$ = 7.2 Hz, CH_3 in *i*Pr), 1.69 (6 H, d, $^3J_{HH}$ = 7.0 Hz, CH_3 in *i*Pr),1.78 (3 H, d, $^3J_{HH}$= 1.0 Hz, CCH_3), 2.33 (6 H, s, CH_3,imidazoline backbone), 3.13 (2 H, sept, $^3J_{HH}$ = 6.9 Hz, C*H*Me_2 in Dipp), 4.64 (2 H, sept, $^3J_{HH}$= 7.1 Hz, C*H*Me_2 in imidazoline), 7.01−7.11(3 H, m, aryl-CH).

^{13}C{^{1}H} NMR (THF-d$_8$, 100 MHz, 293 K): δ = 10.1 (C*C*H_3, imidazoline backbone), 11.5 (C*C*H_3), 11.5 (C*C*H_3), 21.6 (*C*H_3 in *i*Pr), 21.9 (*C*H_3 in *i*Pr), 23.8 (*C*H_3 in *i*Pr), 26.3 (*C*H_3 in *i*Pr), 29.4 (*C*HMe_2 in Dipp), 51.6 (*C*HMe_2 in imidazoline), 115.0, 123.2 (aryl-CH), 123.5, 124.5, 125.0, 126.7 (aryl-CH), 140.3, 148.0 (N_3*C*).

^{11}B{^{1}H} NMR (THF-d$_8$, 128 MHz, 293 K): δ = 35.8 ($\omega_{1/2}$ = 330 Hz).

IR: see Figure S35.

Elemental analysis was performed with samples of **12** · 3 C_6H_6 as obtained from the crystallization procedure. Calculated for $C_{45}H_{61}BN_4Se$: ≡ **12** · 3 C_6H_6: C 72.28, H 8.22, N 7.49. Found: C 72.59, H 8.79, N 8.05.

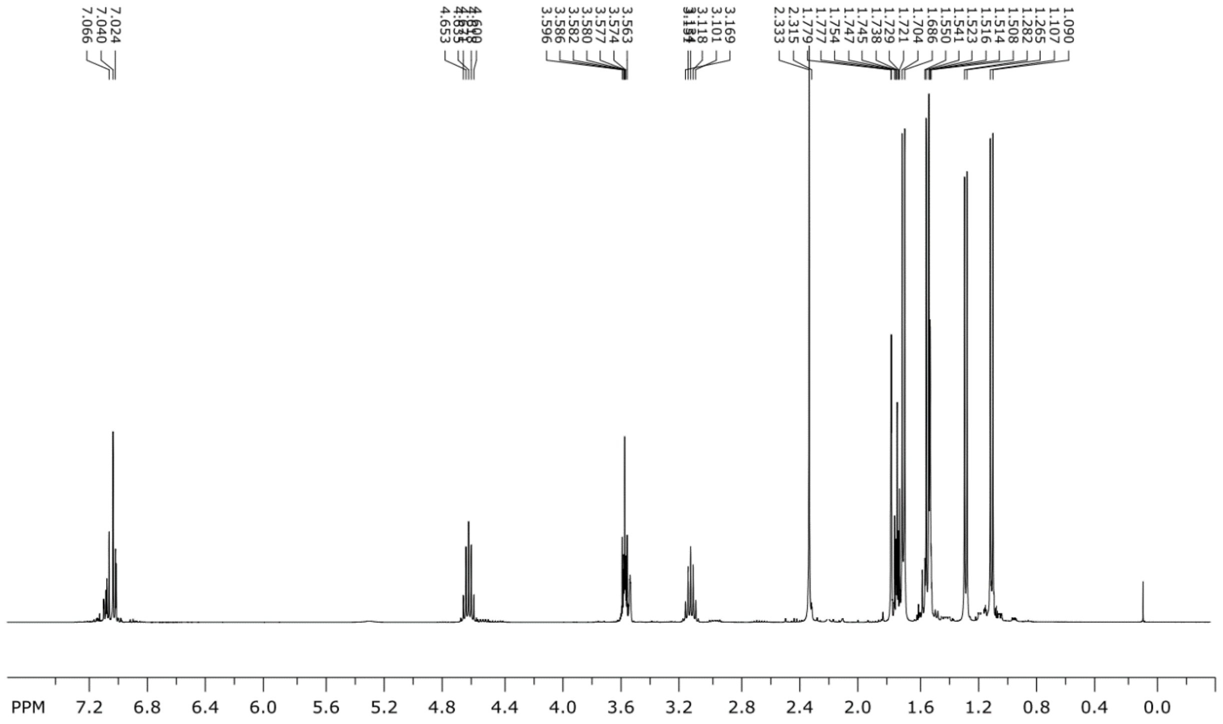

Figure S32. ^{1}H NMR (THF-d$_8$, 400 MHz, 293 K) of compound **12**.

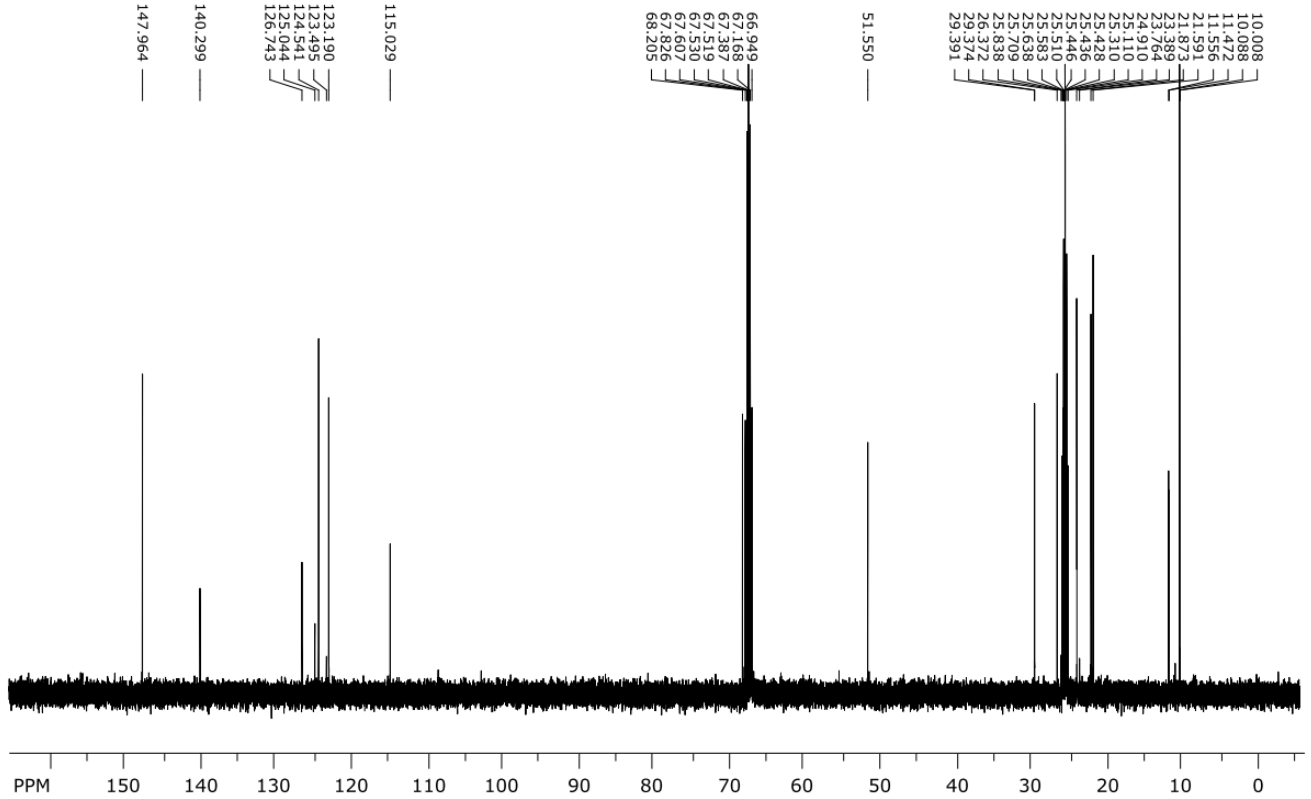

Figure S33. $^{13}C\{^{1}H\}$ NMR (THF-d_8, 100 MHz, 293 K) of compound **12**.

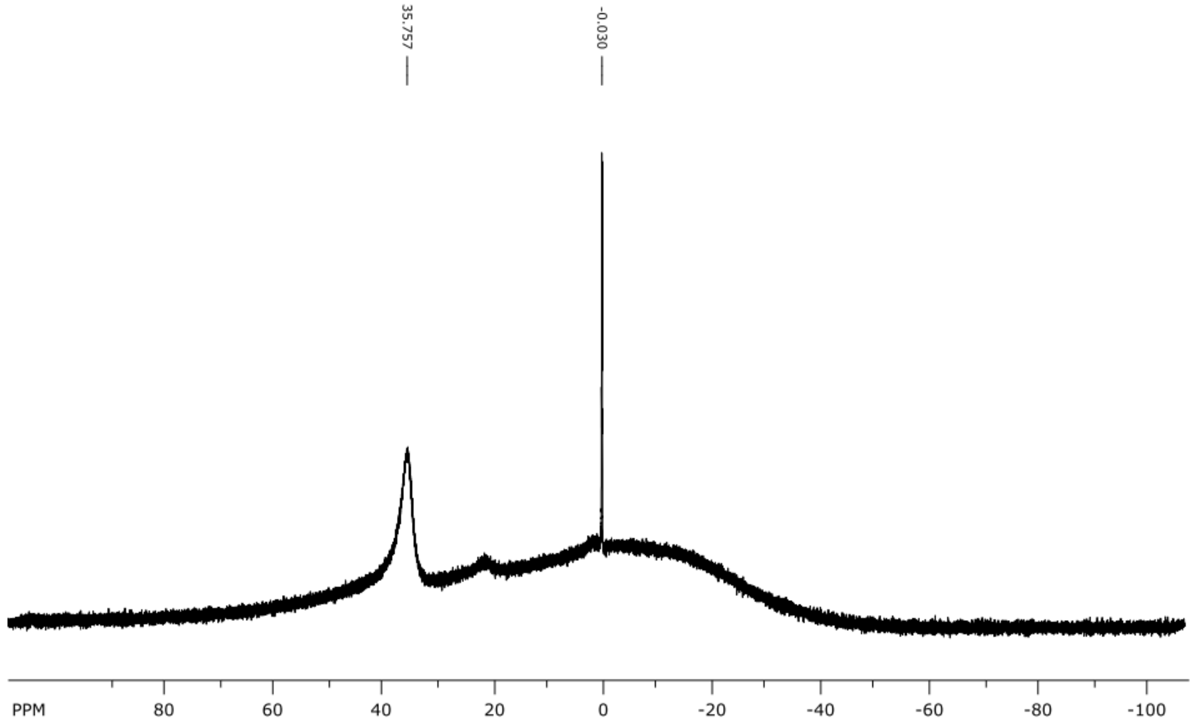

Figure S34. $^{11}B\{^{1}H\}$ NMR (THF-d_8, 128 MHz, 293 K) of compound **12**. The spectrum additionally shows the external reference $Et_2O \cdot BF_3$ with a referenced chemical shift of δ_B = 0.000 ppm.

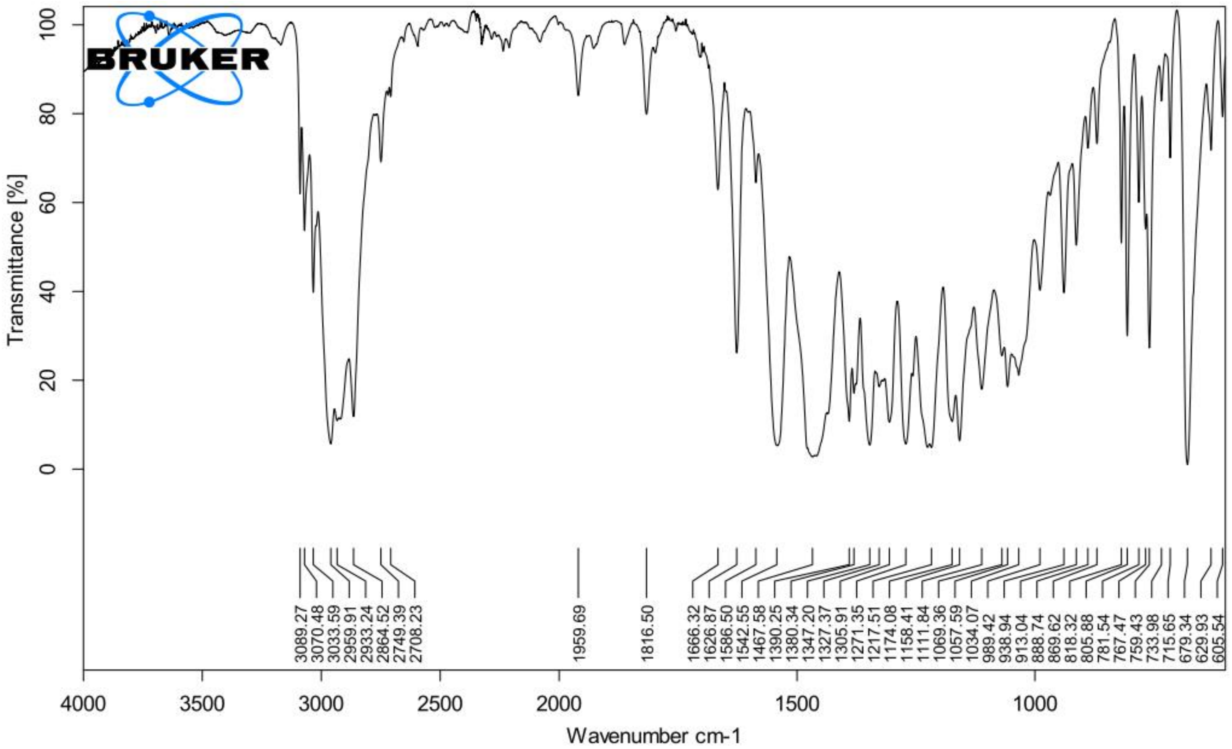

Figure S35. FT-IR spectrum (KBr disc) of compound **12**.

*1.8. Synthesis of Compound **13**.*

Compound **7-Br** (200 mg, 0.33 mmol, 1 eq.) was suspended in 1,2-dimethoxy-ethane (10 mL) and Li_2Te (93 mg, 0.66 mmol, 2 eq.) was added. The suspension was stirred ambient temperature for 48 h. Volatile components were removed *in vacuo*, and the product **13** was extracted with benzene (15 mL) by cannula-filtration. The filtrate was condensed to a volume of ca. 5 mL under reduced pressure. Slow solvent evaporation gave colorless crystals (also suitable for X-ray crystallography) of the composition **13** · 3 C_6H_6 (58 mg, 0.075 mmol, 23 %).

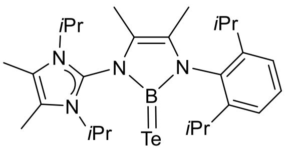

13

In contrast to compounds **11** and **12** the NMR spectra were measured in C_6D_6, in which compound **13** shows limited solubility and prevented the record of meaningful $^{13}C\{^1H\}$ NMR spectra. More polar solvents (THF-d_8 and CD_2Cl_2) lead to decomposition of **13**.

^1H NMR (C_6D_6, 300 MHz, 293 K): δ = 0.89 (6 H, d, $^3J_{HH}$ = 7.2 Hz, CH_3 in *i*Pr), 1.30 (6 H, d, $^3J_{HH}$ = 7.0 Hz, CH_3 in *i*Pr), 1.42 (6 H, s, CH_3, imidazoline backbone), 1.56 (3 H, s, CCH_3), 1.66 (6 H, d, $^3J_{HH}$ = 7.0 Hz, CH_3 in *i*Pr), 1.72 (3 H, s, CCH_3), 1.76 (6 H, d, $^3J_{HH}$ = 6.9 Hz, CH_3 in *i*Pr), 3.54 (2 H, sept, $^3J_{HH}$ = 6.9 Hz, C*H*Me_2 in Dipp), 4.63 (2 H, sept, $^3J_{HH}$ = 7.2 Hz, C*H*Me_2 in imidazoline), 7.29−7.34 (3 H, m, aryl-CH).

$^{11}B\{^1H\}$ NMR (C_6D_6, 96 MHz, 293 K): δ = 30.2 ($\omega_{1/2}$ = 680 Hz).

IR: see Figure S38.

Elemental analysis was performed with samples obtained from the crystallization procedure.
Calculated for $C_{45}H_{61}BN_4Te$: ≡ **13** · 3 C_6H_6: C 67.87, H 7.72, N 7.04. Found: C 68.33, H 8.26, N 6.54.

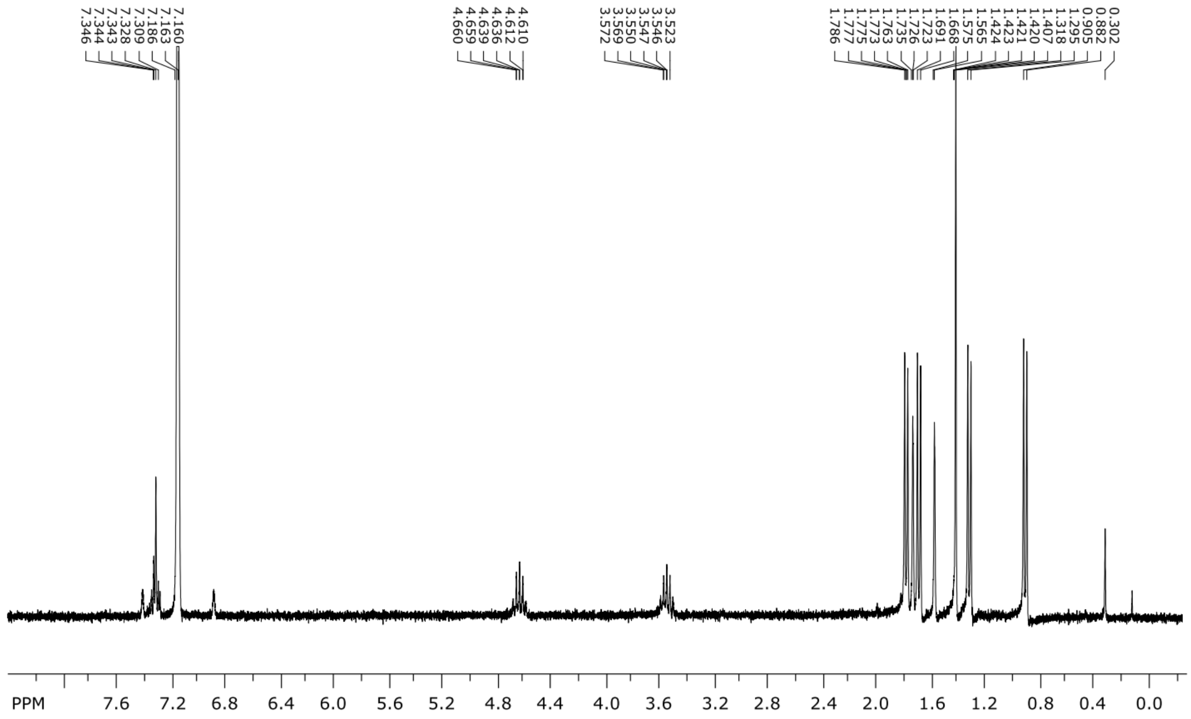

Figure S36. ^1H NMR (C_6D_6, 300 MHz, 293 K) of compound **13** · 3 C_6H_6.

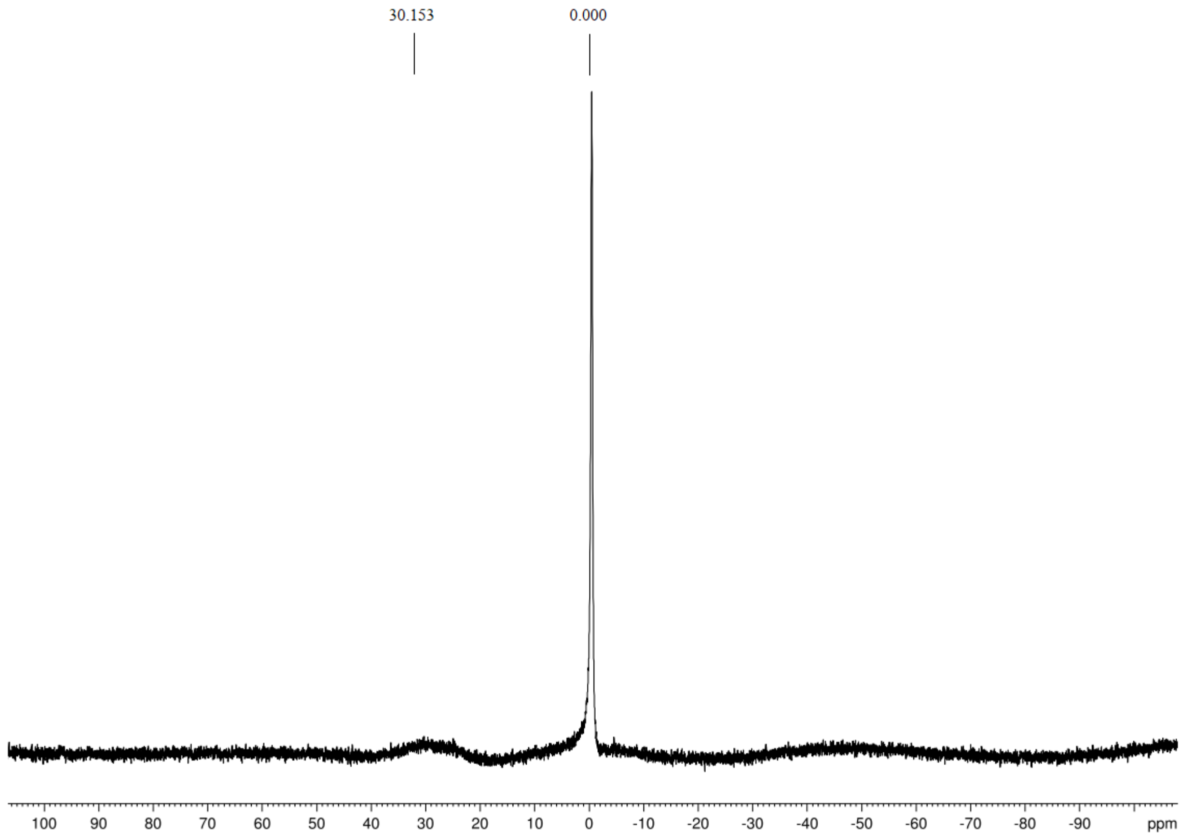

Figure S37. $^{11}B\{^{1}H\}$ NMR (C_6D_6, 96 MHz, 293 K) of compound **13** · 3 C_6H_6. The spectrum additionally shows the external reference $Et_2O \cdot BF_3$ with a referenced chemical shift of δ_B = 0.000 ppm.

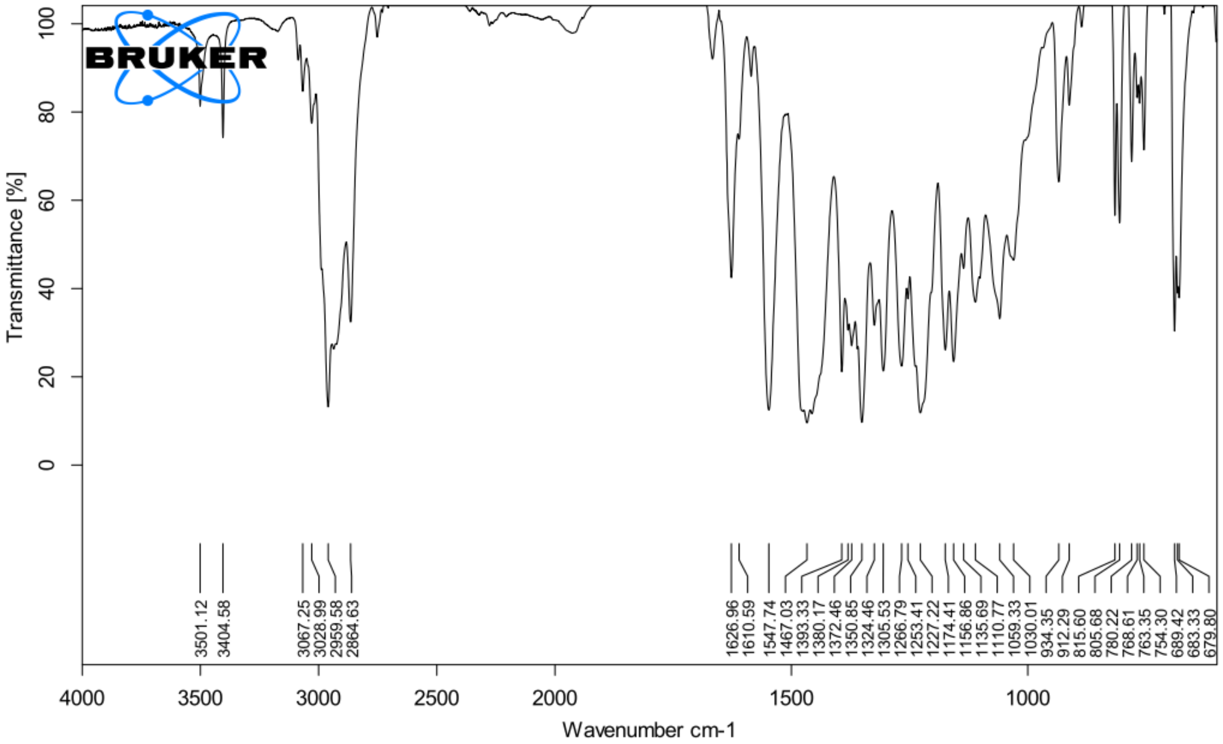

Figure S38. FT-IR spectrum (KBr disc) of compound **13** · 3 C_6H_6.

*1.9. Synthesis of Compound **14**.*

Compound **10** (250 mg, 0.55 mmol, 1 eq.) was dissolved in 1,2-dimethoxyethane (10 mL). A portion of *tert*-butylamine (*t*BuNH$_2$, $C_4H_{11}N$, 120 mg, 0.66 mmol, 3 eq.) and the water trapping reagent $MgSO_4$ (300 mg) were added. The suspension was stirred for 2 h and all volatile components were removed *in vacuo*. The solid was extracted with benzene (15 mL) and $HBF_4{\cdot}Et_2O$ (90 mg, 1 eq.) was added. Slow solvent evaporation gave colorless crystals of compound **14** (212 mg, 65 %), which were also suitable for X-ray crystallography.

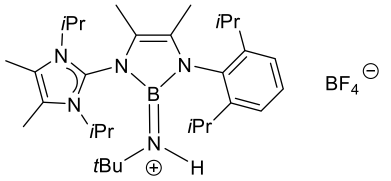

14

^{1}H NMR ($CDCl_3$, 400 MHz, 293 K): δ = 0.83 [9 H, s, C(CH_3)$_3$], 1.16 (6 H, d, $^3J_{HH}$ = 7.2 Hz, CH_3 in *i*Pr), 1.20 (6 H, d, $^3J_{HH}$ = 7.0 Hz, CH_3 in *i*Pr), 1.53 (3 H, s, CCH_3), 1.62 (6 H, d, $^3J_{HH}$ = 7.0 Hz, CH_3 in *i*Pr), 1.63 (6 H, d, $^3J_{HH}$= 6.9 Hz, CH_3 in *i*Pr), 1.73 (3 H, s, CCH_3), 2.14 (1 H, s, NH), 2.48 (6 H, s, CH_3, imidazoline backbone), 2.91 (2 H, sept, $^3J_{HH}$= 6.9 Hz, C*H*Me$_2$ in Dipp), 4.57 (2 H, sept, $^3J_{HH}$= 7.2 Hz, C*H*Me$_2$ in imidazoline), 7.20 (2 H, d, $^3J_{HH}$ = 9.0 Hz, aryl-CH), 7.32 (1 H, t, $^3J_{HH}$ = 9.0 Hz, aryl-CH).

^{13}C{^{1}H} NMR ($CDCl_3$, 100 MHz, 293 K): δ = 10.5 (C*C*H$_3$, imidazoline backbone), 10.8 (C*C*H$_3$), 11.1 (C*C*H$_3$), 21.2 (*C*H$_3$ in *i*Pr), 21.6 (*C*H$_3$ in *i*Pr), 23.9 (*C*H$_3$ in *i*Pr), 24.3 (*C*H$_3$ in *i*Pr), 28.2 (*C*HMe$_2$ in Dipp), 32.7 [C(*C*H$_3$)$_3$], 49.5 (*C*HMe$_2$ in imidazoline), 50.7 [*C*(CH$_3$)$_3$], 114.7, 124.0 (aryl-CH), 124.5, 126.2, 128.2 (aryl-CH), 135.1, 137.9, 147.0 (N_3*C*).

^{11}B{^{1}H} NMR ($CDCl_3$, 128 MHz, 293 K): δ_B = 22.9 ($\omega_{1/2}$ = 650 Hz, B=N), −1.0 ($\omega_{1/2}$ = 3 Hz, BF_4^-).

Elemental analysis was performed with crystalline samples dried *in vacuo* for 24 h.
Calculated for $C_{31}H_{53}B_2F_4N_5$: C 62.75, H 9.00, N 11.80. Found: C 62.48, H 8.86, N 11.66.

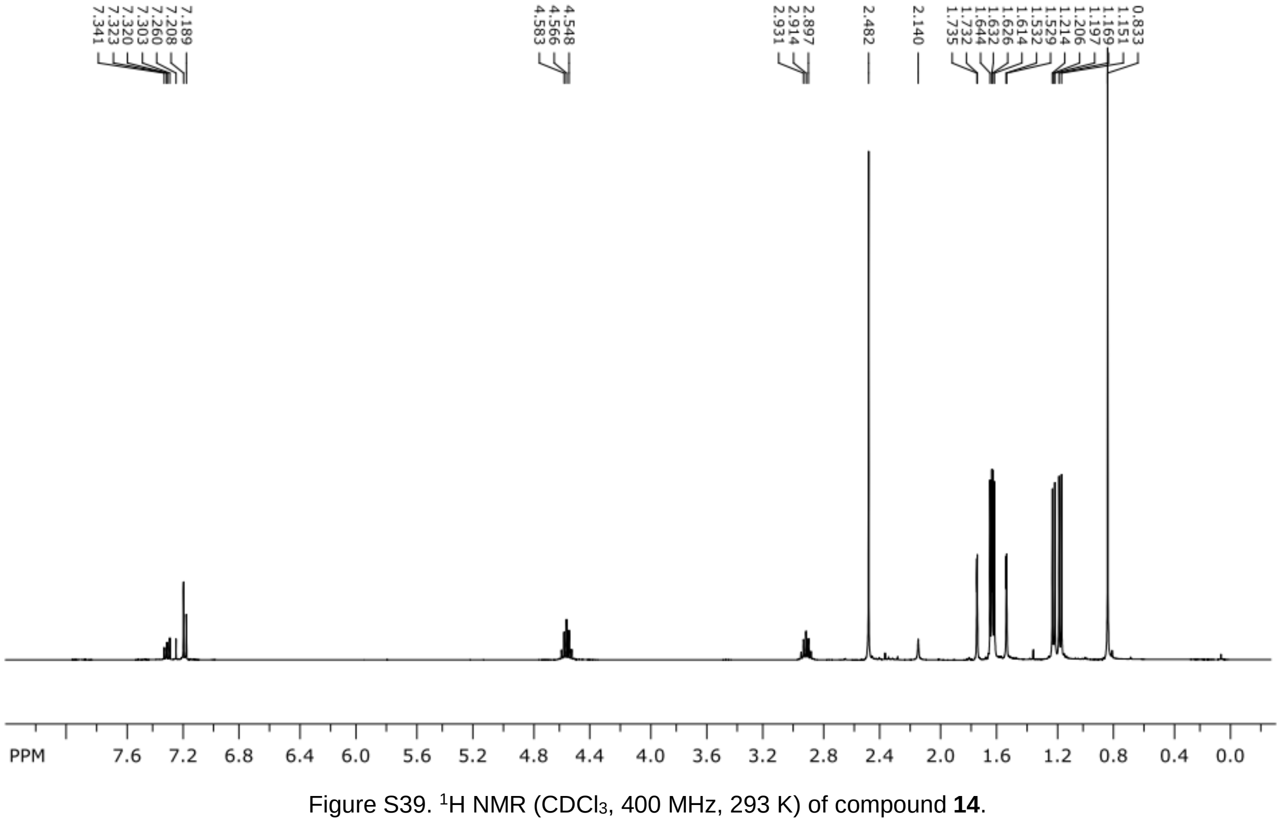

Figure S39. ^{1}H NMR ($CDCl_3$, 400 MHz, 293 K) of compound **14**.

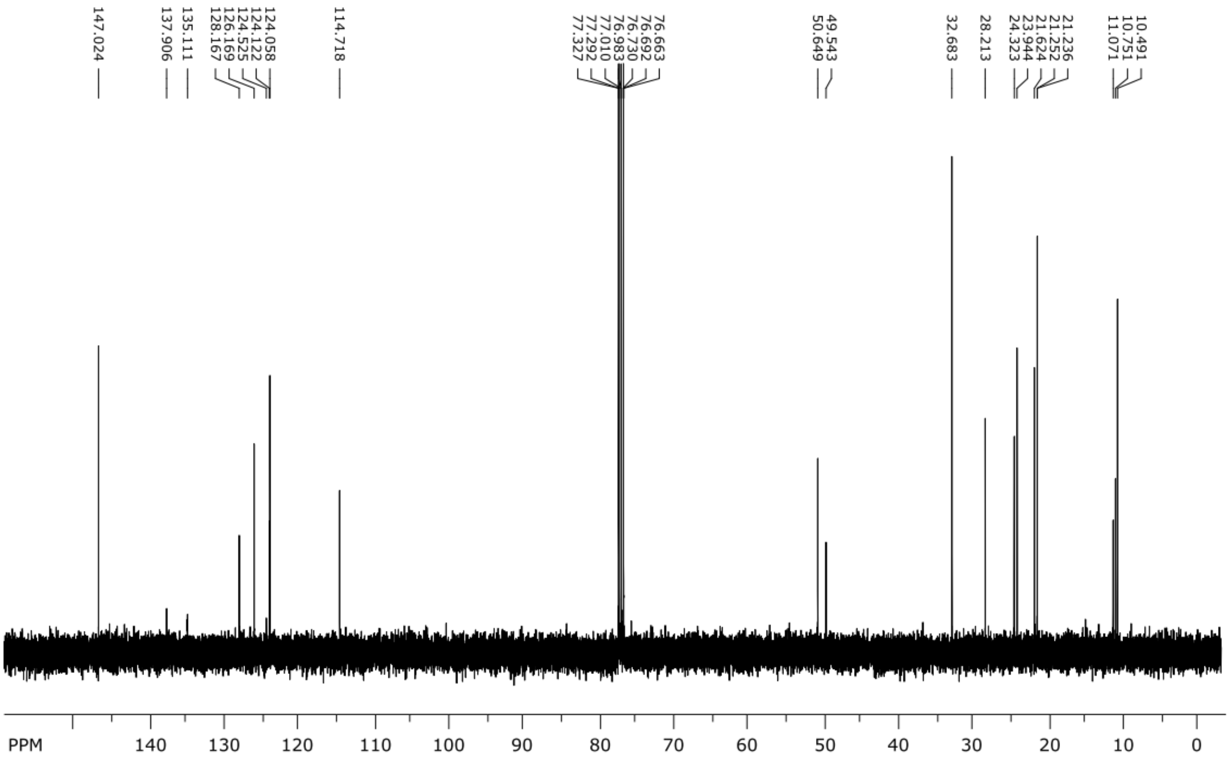

Figure S40. $^{13}C\{^1H\}$ NMR ($CDCl_3$, 100 MHz, 293 K) of compound **14**.

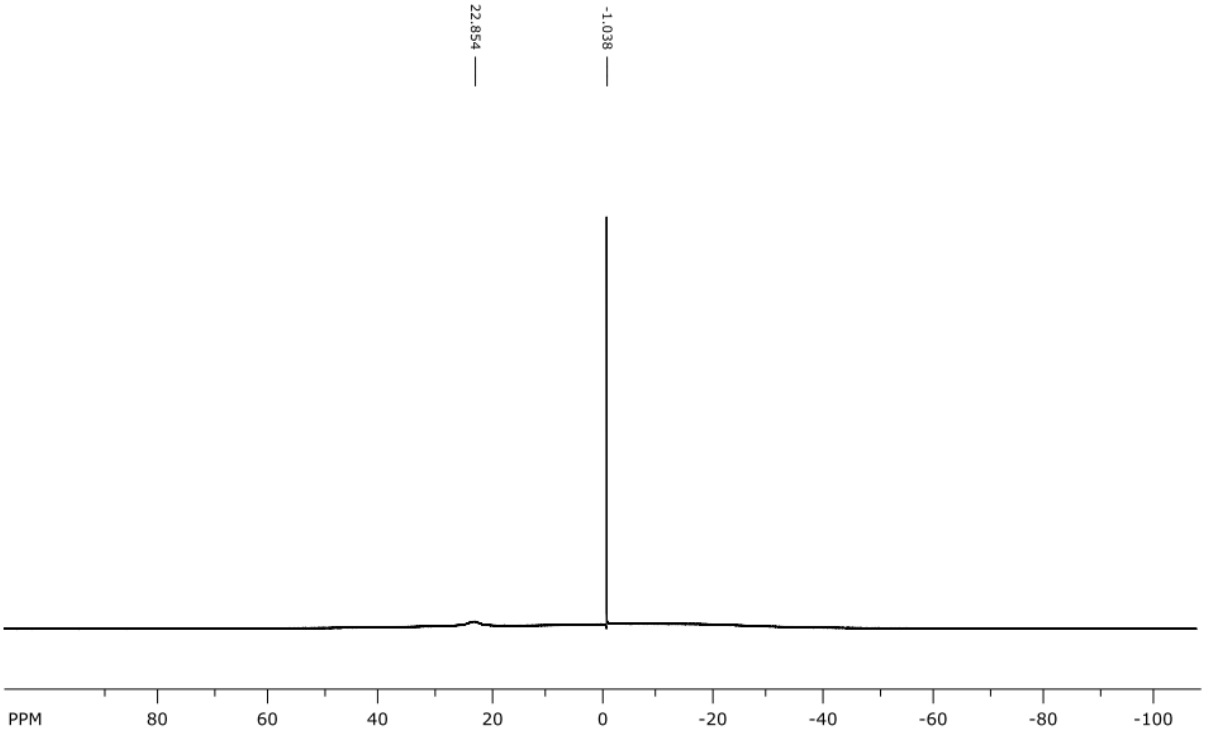

Figure S41. $^{11}B\{^1H\}$ NMR ($CDCl_3$, 128 MHz, 293 K) of compound **14**.

*1.10. Synthesis of Compound **15**.*

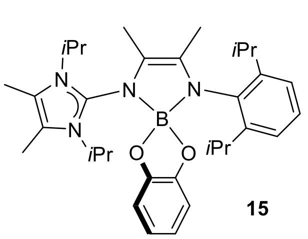

Compound **10** (250 mg, 0.55 mmol, 1 eq.) was dissolved in 1,2-dimethoxyethane (10 mL). Catechol [(1,2-$(HO)_2C_6H_4$, 73 mg, 0.66 mmol, 1.2 eq.] and the water trapping reagent $MgSO_4$ (300 mg) were added. The suspension was vigorously stirred at ambient temperature for 6 h. The solvent was removed *in vacuo* and the solid extracted with $CHCl_3$ (20 mL) and filtered over Celite. The solution was reduced *in vacuo* (to ca. 0.5 mL) and layered with *n*-pentane (20 mL). Slow solvent diffusion gave colorless crystals, which were also found suitable for X-ray crystallographic analysis. The colorless crystals were found to have the composition **15**·$CHCl_3$ (278 mg, 75 %). For NMR measurements and elemental analysis the crystalline samples were finely ground to powder with mortar and pestle and dried in high vacuum for 24 h to remove $CHCl_3$ trapped in in the crystal lattice.

^{1}H NMR (C_6D_6, 400 MHz, 293 K): δ = 0.92 (6 H, d, $^3J_{HH}$ = 7.2 Hz, CH_3 in *i*Pr), 1.25 (6 H, s, CH_3, imidazoline backbone), 1.26 (6 H, d, $^3J_{HH}$ = 7.0 Hz, CH_3 in *i*Pr), 1.37 (6 H, d, $^3J_{HH}$ = 7.0 Hz, CH_3 in *i*Pr), 1.59 (6 H, d, $^3J_{HH}$ = 6.9 Hz, CH_3 in *i*Pr), 1.72 (3 H, s, CCH_3), 1.75 (3 H, s, CCH_3), 4.20 (2 H, sept, $^3J_{HH}$ = 6.9 Hz, C*H*Me$_2$ in Dipp), 5.41 (2 H, sept, $^3J_{HH}$ = 7.1 Hz, C*H*Me$_2$ in imidazoline), 6.57−6.60 (2 H, m, aryl-CH in catechol), 6.68−6.71 (2 H, m, aryl-CH in catechol), 7.29−7.34 (3 H, m, aryl-CH).

^{13}C{^{1}H} NMR (C_6D_6, 100 MHz, 293 K): δ = 9.7 (C*C*H$_3$, imidazoline backbone), 12.3 (C*C*H$_3$), 12.6 (C*C*H$_3$), 21.4 (*C*H$_3$ in *i*Pr), 22.0 (*C*H$_3$ in *i*Pr), 25.5 (*C*H$_3$ in *i*Pr), 26.2 (*C*H$_3$ in *i*Pr), 28.6 (*C*HMe$_2$ in Dipp), 49.3 (*C*HMe$_2$ in imidazoline), 106.5, 108.3 (aryl-CH), 118.0 (aryl-CH), 122.0, 123.7 (aryl-CH), 126.5 (aryl-CH), 139.9, 146.0 (N$_3$*C*), 150.6, 153.8.

^{11}B{^{1}H} NMR (C_6D_6, 128 MHz, 293 K): δ_B = 12.0 ($\omega_{1/2}$ = 47 Hz).

Elemental analysis. Calculated for $C_{33}H_{47}BN_4O_2$: C 73.05, H 8.73, N 10.33. Found: C 73.23, H 8.66, N 10.35.

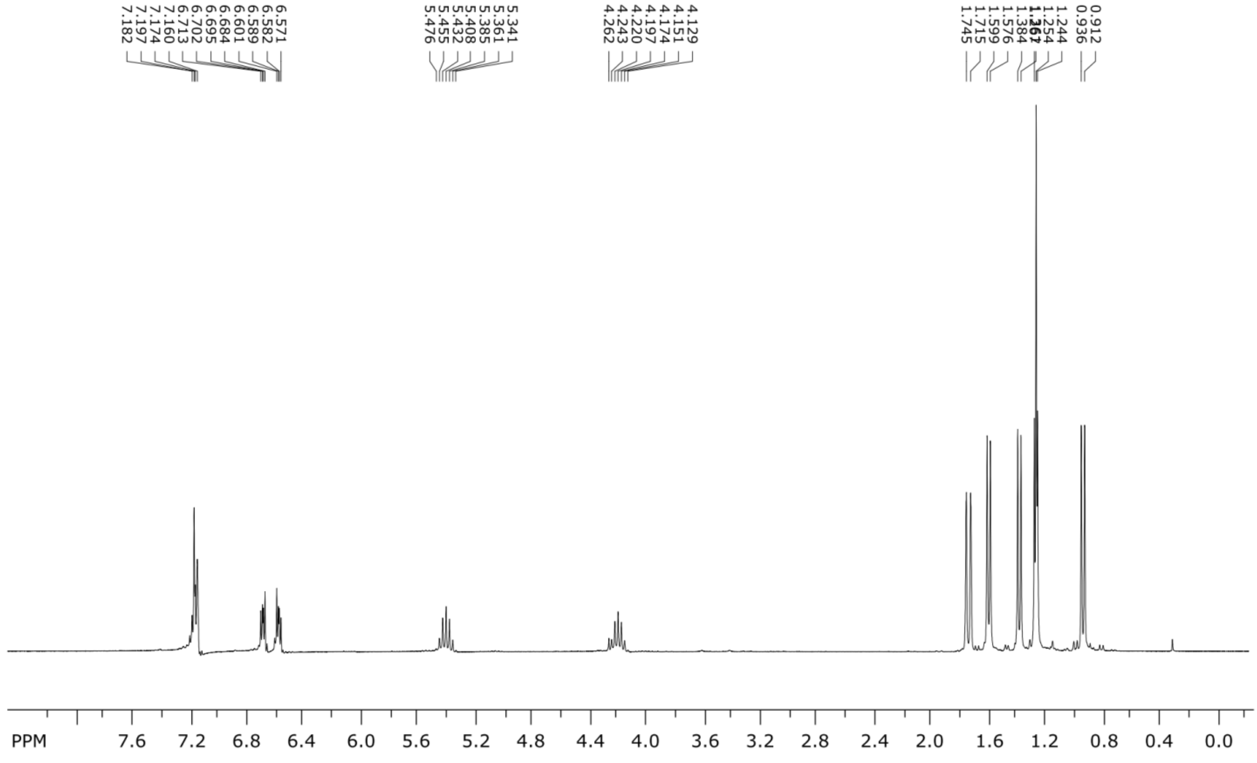

Figure S42. ^{1}H NMR (C_6D_6, 400 MHz, 293 K) of compound **15**.

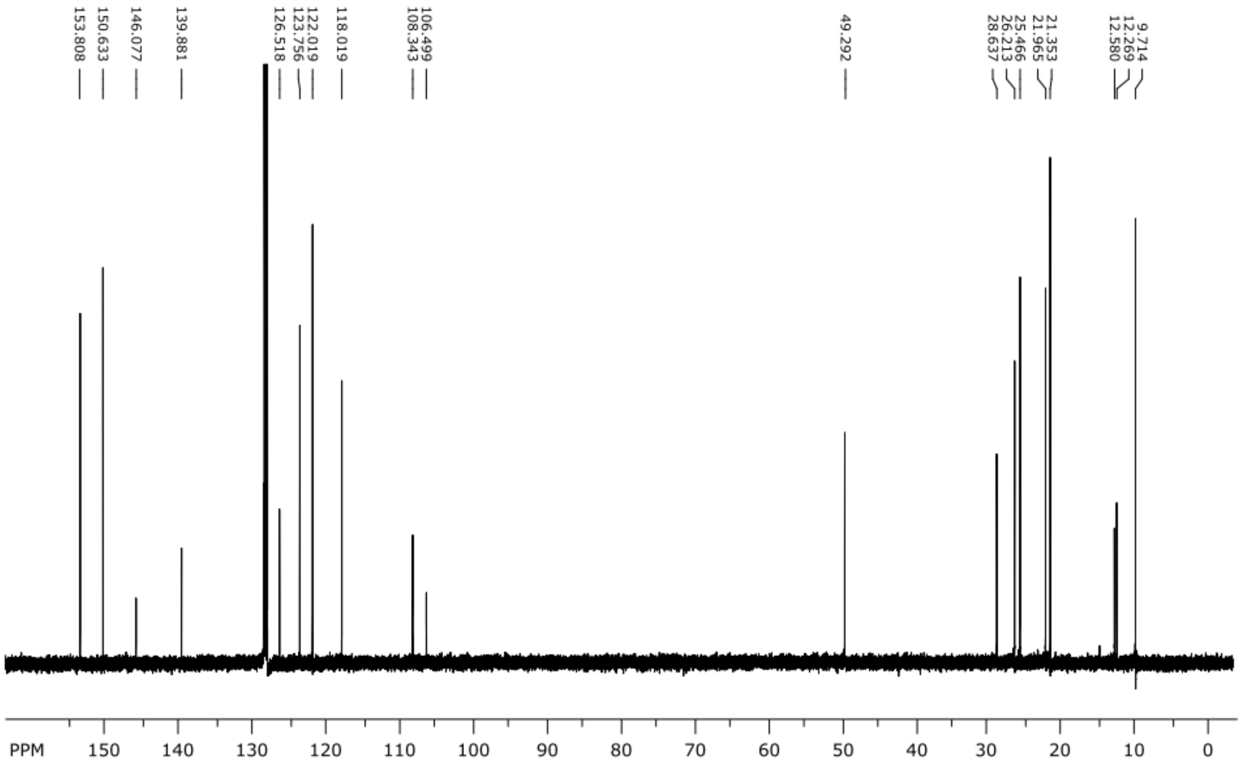

Figure S43. $^{13}C\{^1H\}$ NMR (C_6D_6, 100 MHz, 293 K) of compound **15**.

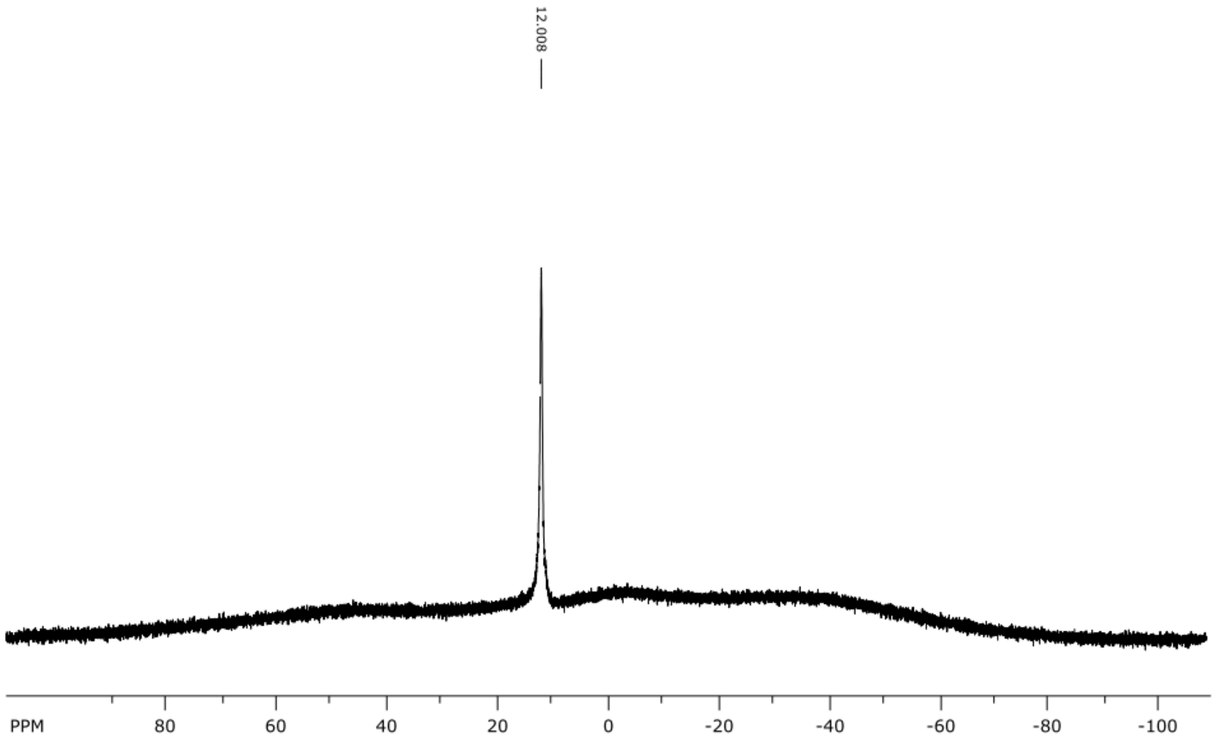

Figure S44. $^{11}B\{^1H\}$ NMR (C_6D_6, 128 MHz, 293 K) of compound **15**.

*1.11. Synthesis of Compound **16**.*

Compound **10** (250 mg, 0.55 mmol, 1 eq.) was dissolved in toluene (10 mL). Trimethylsilyl cyanide (Me_3SiCN, 120 mg, 1.21 mmol, 2.2 eq.) was added and the resulting solution was stirred at ambient temperature for 6 h. The solution was reduced *in vacuo* (to ca. 0.5 mL) and layered with *n*-pentane (20 mL). Slow solvent diffusion gave colorless crystals of compound **16**, which were also found suitable for X-ray crystallographic analysis (147 mg, 55 %).

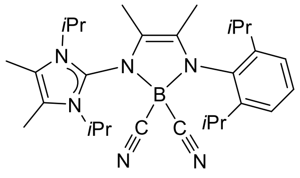

16

^{1}H NMR ($CDCl_3$, 400 MHz, 293 K): δ = 1.26 (6 H, d, $^3J_{HH}$ = 7.2 Hz, CH_3 in *i*Pr), 1.45 (6 H, d, $^3J_{HH}$ = 7.0 Hz, CH_3 in *i*Pr), 1.54 (3 H, s, CCH_3), 1.63 (6 H, d, $^3J_{HH}$ = 7.0 Hz, CH_3 in *i*Pr), 1.79 (3 H, s, CCH_3), 1.80 (6 H, d, $^3J_{HH}$ = 6.9 Hz, CH_3 in *i*Pr), 2.43 (6 H, s, CH_3, imidazoline backbone), 3.63 (2 H, sept, $^3J_{HH}$ = 6.9 Hz, C*H*Me$_2$ in Dipp), 5.37 (2 H, sept, $^3J_{HH}$ = 7.1 Hz, C*H*Me$_2$ in imidazoline), 7.24−7.35 (3 H, m, aryl-CH).

^{13}C{^{1}H} NMR ($CDCl_3$, 100 MHz, 293 K): δ = 10.1 (C*C*H$_3$, imidazoline backbone), 11.6 (C*C*H$_3$), 12.2 (C*C*H$_3$), 21.3 (*C*H$_3$ in *i*Pr), 22.4 (*C*H$_3$ in *i*Pr), 25.0 (*C*H$_3$ in *i*Pr), 25.4 (*C*H$_3$ in *i*Pr), 27.9 (*C*HMe$_2$ in Dipp), 49.5 (*C*HMe$_2$ in imidazoline), 123.1, 123.6 (aryl-CH), 123.8, 126.3, 128.0, 137.9 (aryl-CH), 148.9 (N_3*C*), 150.1, not observed B−*C*≡N.

^{11}B{^{1}H} NMR ($CDCl_3$, 128 MHz, 293 K): δ_B = −8.5 ($\omega_{1/2}$ = 54 Hz).

Elemental analysis. Calculated for $C_{29}H_{43}BN_6$: C 71.60, H 8.91, N 17.27. Found: C 71.25, H 8.85, N 17.35.

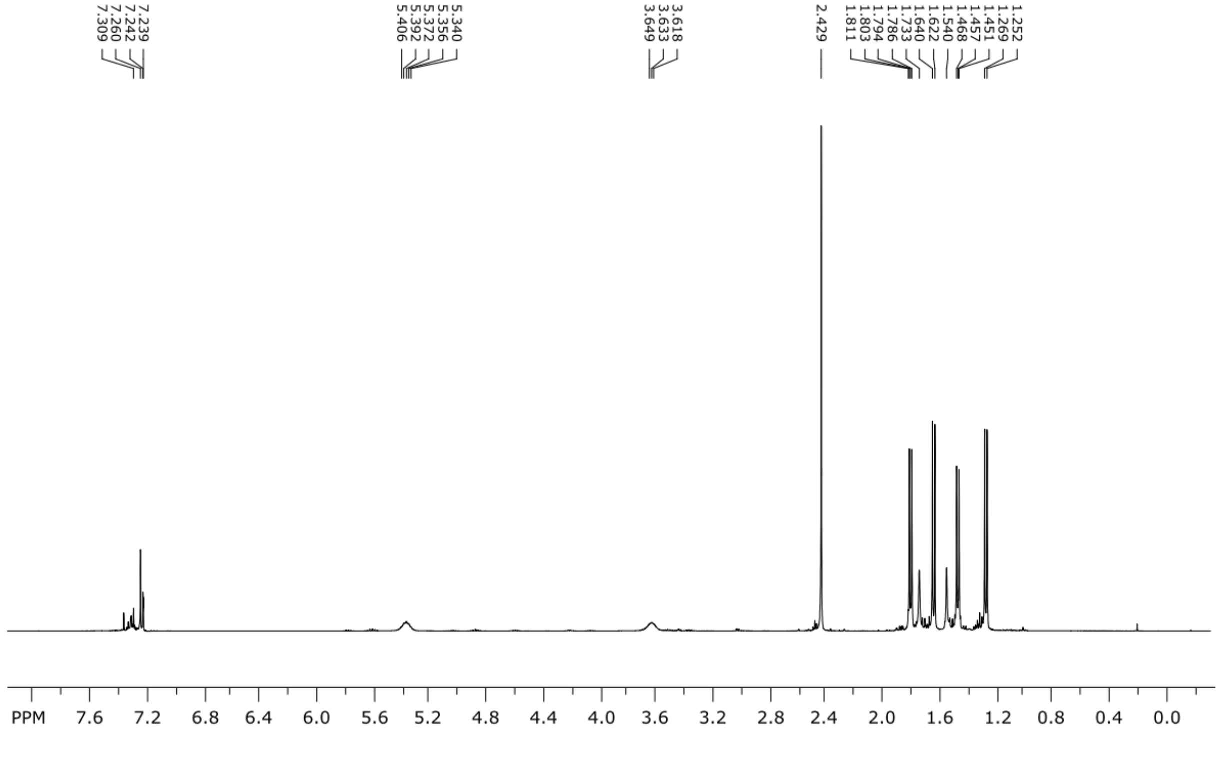

Figure S45. ^{1}H NMR ($CDCl_3$, 400 MHz, 293 K) of compound **16**.

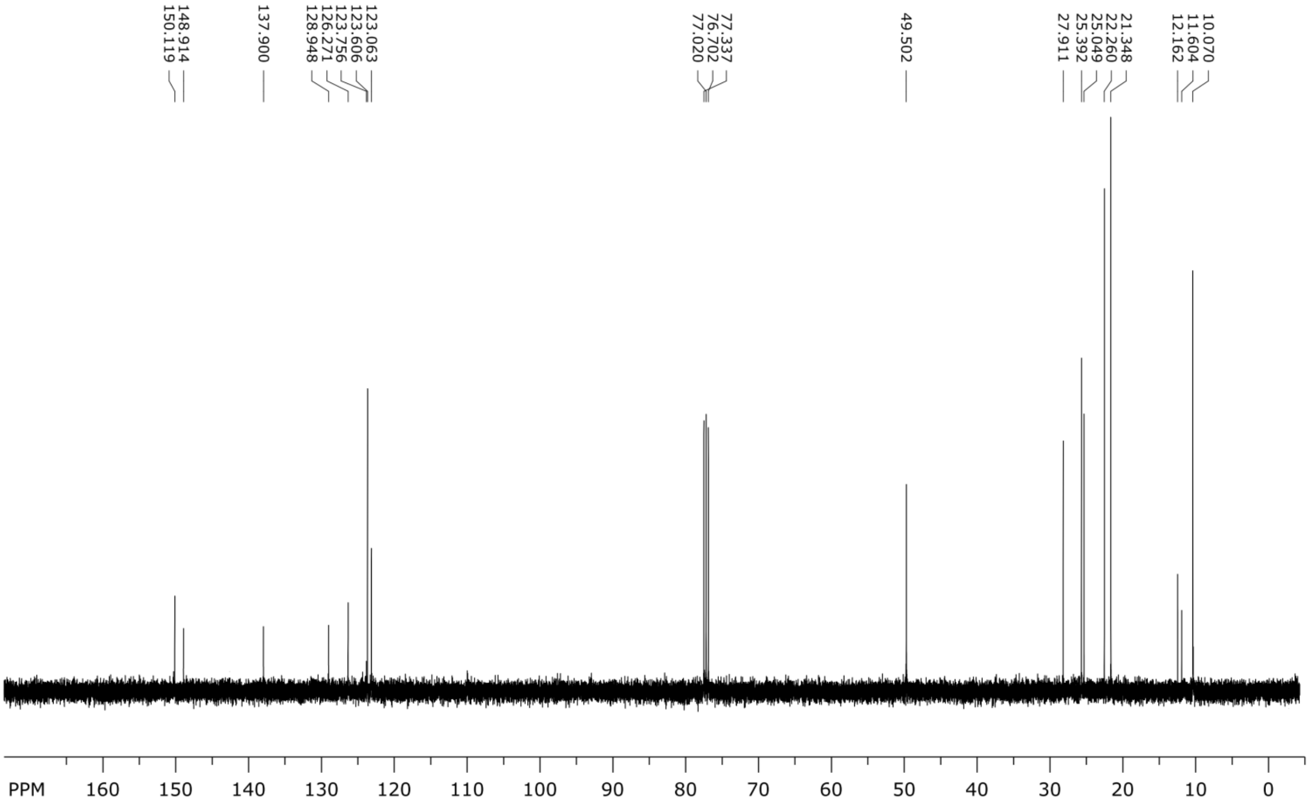

Figure S46. $^{13}C\{^{1}H\}$ NMR ($CDCl_3$, 100 MHz, 293 K) of compound **16**.

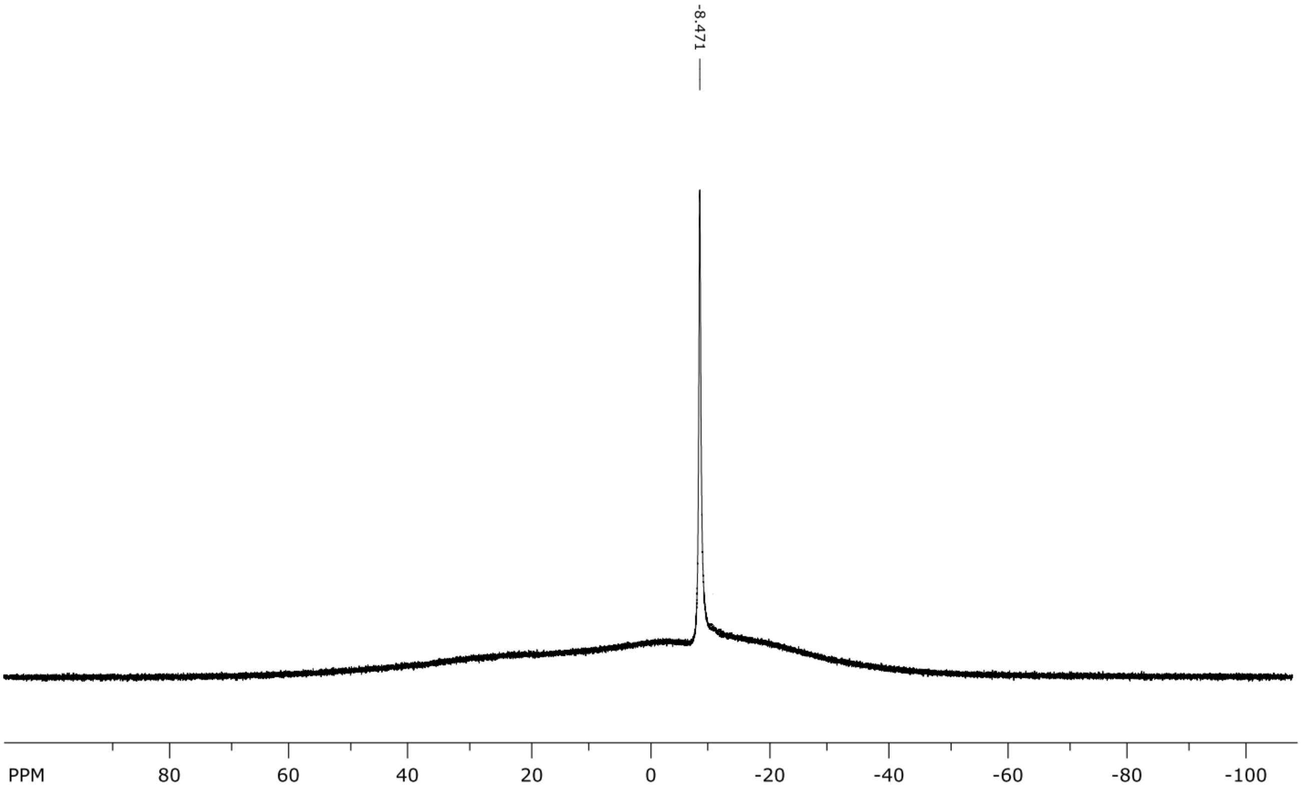

Figure S47. $^{11}B\{^{1}H\}$ NMR ($CDCl_3$, 128 MHz, 293 K) of compound **16**.

*1.12. Synthesis of Thioborane **11** from Oxoborane **10***

Compound **10** (250 mg, 0.55 mmol, 1 eq.) was dissolved in 1,2-dimethoxyethane (10 mL), and the water trapping reagent $MgSO_4$ (300 mg) was added. Hydrogen sulfide (H_2S, anhydrous) was bubbled through the suspension for 5 min. The reaction was stirred at ambient temperature for 6 h and filtered over Celite. All volatile material was removed *in vacuo* to afford a colorless solid **11** (218 mg, 85 %). The ^1H and $^{11}B\{^1H\}$ NMR spectra recorded in THF-d_8 were identical to those of compound **11**.

3. Quantum Mechanical Calculations

3.1. Detailed computational methods

All four compounds **10**-**13** (X = O, S, Se, Te) were optimized using several different combinations of DFT methods and basis sets, to assess the impact of the choice of DFT functional / basis set on the obtained results. The starting points for all calculations were the respective crystal structures. We used modern ωB97XD, [3] M06 [4] and CAM-B3LYP [5] functionals with two basis sets: a double-zeta 6-31G** basis set on all atoms and LANL2DZ ECP [6] on Te (if present) and a triple-zeta 6-311G++** basis set on all atoms and def2-TZVP ECP on Te (if present). The influence of the effective core potentials on the result for the Te system was investigated with additional calculations for this system using the same basis sets but with replacement of the LANL2DZ ECP by the WTBS basis set [7,8] obtained from the basis set exchange. [9] Since the results from all chosen combinations of DFT functional and basis sets were similar, we decided to base the discussion on the wB97XD/6-311G++** (with WTBS on Te atom if present) calculations. Selected results from all other methods are presented below. All calculations were performed using Gaussian 09 software [10]. We used ultrafine grid for DFT and standard optimization and convergence parameters as implemented in Gaussian 09. Bond indices, partial charges and orbital localization calculations using Foster-Boys approach [11] were performed using the Multiwfn Version 3.7 software [12]. Orbital visualization was performed using the VMD ver. 1.9.3 software [13].

3.2. Computational Results for Compounds ***10−13***.

The B=X computationally obtained bond distances calculated at different levels of theory are presented in Table S1. A comparison with the structural metrics obtained from crystal structures suggests excellent accuracy with the bond lengths obtained from the medium-sized basis sets. It is worth noting, however, that the calculations were performed for the isolated molecules, while the experimental bond lengths are from crystal structures, where molecules interact with their neighbors and are densely packed, shortening their bond lengths.

The chemical character of the B=X bond was assessed by Meyer and Wiberg bond indices calculations, Tables S2 and S3. Both methods quite consistently assign the double bond to all studied systems in virtually all combinations of DFT methods and basis sets.

The natural population analysis (NPA) partial charges for B and X atoms are presented in Table 4. Similarly to the previously studied oxoborane case [14] the results for the B-O system suggest a double bond which is heavily polarized towards oxygen (NPA partial charge of -1.02 for O and 1.02 for B). The description is similar for all other B-X cases, but as shown previously the polarization become weaker as the terminal heteroatom becomes heavier, since for S the NPA partial charge is equal to -0.72, for Se it is equal to -0.58 to finally reach -0.31 for Te.

Tables S6 and S7 lists Meyer and Wiberg bond indices for the previously synthesized systems bearing a B=X bond, discussed in this work. The previously synthesized compounds are termed as stated:

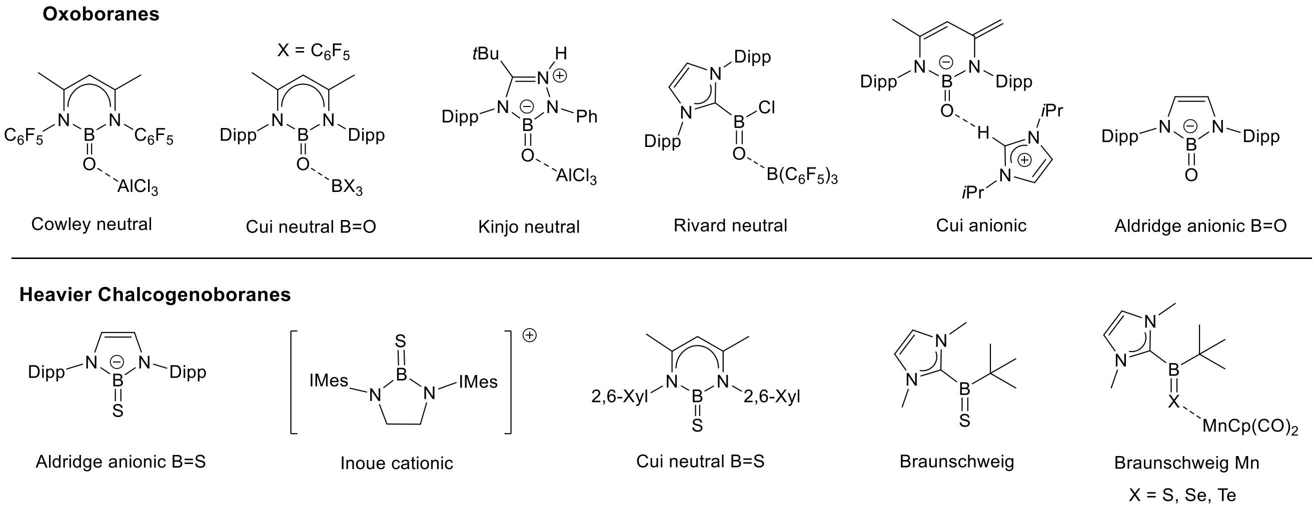

Table S1. Experimental and computed bond lengths in [Å] obtained compounds **5**-**8** with various density functionals. * The bond length was calculated as the average value resulting from three crystallographically independent molecules with the individual bond lengths as stated: B1−Te1 2.151(6) Å, B2−Te2 2.124(7) Å, B3−Te3 2.143(6) Å. m stands for the double-zeta basis set (6-31G** basis set on all atoms and LANL2DZ ECP on Te), t for the triple-zeta basis set (6-311++G** basis set on all atoms and def2-TZVP ECP on Te) and w stands for the triple zeta basis set 6-311++G** for all atoms with the WTBS basis set for Te.

Bond length [Å]	B=O (**10**)	B=S (**11**)	B=Se (**12**)	B=Te (**13**)
Exp. bond length	**1.2867(16)**	**1.754(1)**	**1.909(2)**	**2.139(7)***
ωB97X-D/d	1.279	1.749	1.880	2.128
**ωB97X-D/t**	**1.296**	**1.783**	**1.904**	2.121
ωB97X-D/w	-	-	-	**2.155**
M06/d	1.274	1.750	1.880	2.133
M06/t	1.289	1.784	1.906	2.128
M06/w	-	-	-	2.159
CAM-B3LYP/d	1.276	1.750	1.884	2.134
CAM-B3LYP/t	1.292	1.781	1.904	2.121
CAM-B3LYP/w	-	-	-	2.156

Table S2. Calculated Meyer bond indices.

Bond order	B=O (**10**)	B=S (**11**)	B=Se (**12**)	B=Te (**13**)
ωB97X-D/d	1.79	1.56	1.49	1.50
ωB97X-D/t	**1.78**	**1.68**	**1.65**	1.37
ωB97X-D/w	-	-	-	**1.61**
M06/d	1.78	1.57	1.47	1.52
M06/t	1.80	1.69	1.60	1.36
M06/w	-	-	-	1.61
CAM-B3LYP/d	1.84	1.54	1.47	1.45
CAM-B3LYP/t	1.82	1.69	1.65	1.37
CAM-B3LYP/w	-	-	-	1.57

Table S3. Calculated Wiberg bond indices

Bond order	B=O (**10**)	B=S (**11**)	B=Se (**12**)	B=Te (**13**)
ωB97X-D/d	2.13	1.77	1.69	1.52
ωB97X-D/t	**1.86**	**1.75**	**1.69**	1.53
ωB97X-D/w	-	-	-	**1.69**
M06/d	2.13	1.78	1.70	1.55
M06/t	1.88	1.76	1.68	1.55
M06/w	-	-	-	1.70
CAM-B3LYP/d	2.14	1.76	1.68	1.50
CAM-B3LYP/t	1.88	1.76	1.68	1.53
CAM-B3LYP/w	-	-	-	1.69

Table S4. Calculated partial charges located on B and X atoms.

ωB97X-D/w	B=O (**10**)	B=S (**11**)	B=Se (**12**)	B=Te (**13**)
Hirschfeld B	0.13	0.06	0.04	0.02
Hirschfeld X	-0.47	-0.48	-0.46	-0.44
Mulliken B	0.83	0.54	0.39	0.54
Mulliken X	-0.64	-0.60	-0.40	-0.53
Becke B	0.64	0.44	0.43	0.43
Becke X	-0.78	-1.06	-1.10	-1.20
NAO B	1.02	0.64	0.50	0.22
NAO X	-1.02	-0.72	-0.58	-0.31

Table S5. Calculated frequencies of B=X stretch vibrations [cm^{-1}].

$\bar{\nu}$ (B=X) [cm^{-1}]	B=O (**10**)	B=S (**11**)	B=Se (**12**)	B=Te (**13**)
ωB97X-D/d	1692	1279	1279	1278
ωB97X-D/t	**1651**	**1283**	**1280**	1276
ωB97X-D/w	-	-	-	**1283**
M06/d	1692	1257	1255	1248
M06/t	1648	1261	1258	1254
M06/w	-	-	-	1257
CAM-B3LYP/d	1693	1275	1276	1274
CAM-B3LYP/t	1640	1275	1272	1268
CAM-B3LYP/w	-	-	-	1272

Table S6. Calculated Meyer bond indices for previously synthesized systems at the ωB97X-D/t level of theory.

Bond order	B=O	B=S	B=Se	B=Te
Cowley neutral	1.35	-	-	-
Cui neutral	1.20	-	-	-
Kinjo neutral	1.27	-	-	-
Rivard neutral	1.36	-	-	-
Cui anionic	1.38	-	-	-
Aldridge anionic	1.73	1.63	-	-
Inoue cationic	-	1.85	-	-
Cui neutral	-	1.95	-	-
Braunschweig neutral	-	2.04	-	-
Braunschweig Mn	-	1.72	1.78	1.71

Table S7. Calculated Wiberg bond indices for previously synthesized systems at the ωB97X-D/t level of theory.

Bond order	B=O	B=S	B=Se	B=Te
Cowley neutral	1.48	-	-	-
Cui neutral	1.38	-	-	-
Kinjo neutral	1.44	-	-	-
Rivard neutral	1.54	-	-	-
Cui anionic	1.57	-	-	-
Aldridge anionic	1.80	1.69	-	-
Inoue cationic	-	1.87	-	-
Cui neutral	-	1.91	-	-
Braunschweig neutral	-	2.02	-	-
Braunschweig Mn	-	1.84	1.80	1.82

A thorough investigation of the molecular orbitals was performed for compounds **10**-**13** to obtain a full description of the newly synthesized systems in terms of molecular orbitals theory. Selected orbitals for compound **10** are presented in Figure S39 and include the HOMO−4, HOMO−1, HOMO and LUMO orbitals. The π-bond to support the B=O double bond character can be attributed to the HOMO−4 orbital spanning over the large part of the B,N heterocyclic ring with a partial contribution of the HOMO−1 *p*-orbital, which is mostly localized on the O atom.

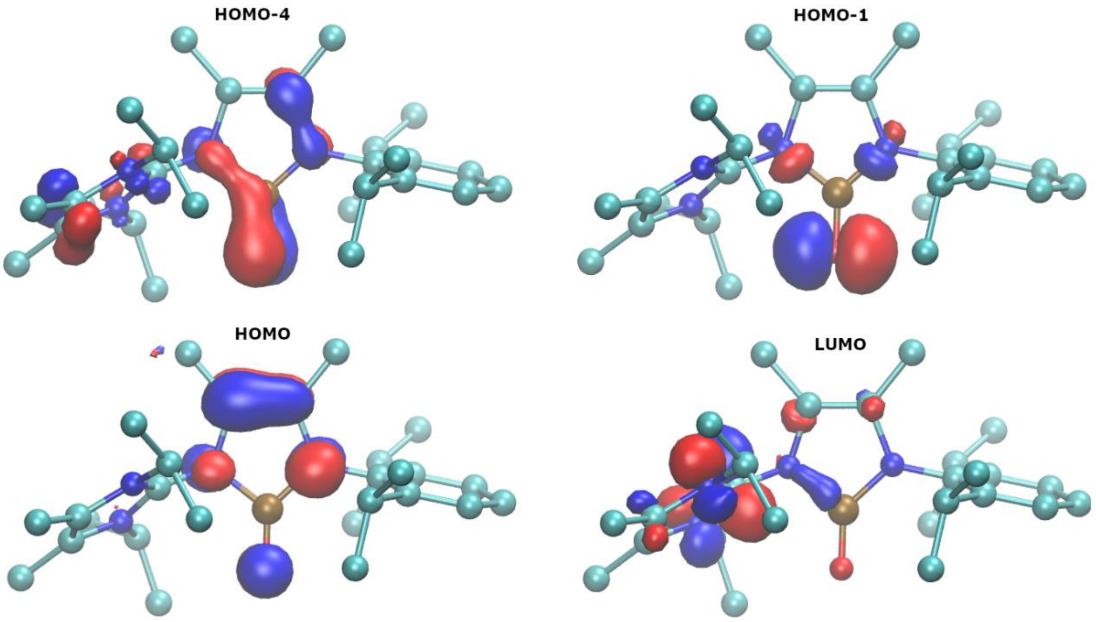

Figure S39. Selected molecular orbitals of compound **10**.

In the case of compound **11** the picture is quite similar, although the order of some crucial orbitals is slightly different, Figure S40. A π-orbital similar to that of HOMO−4 for compound **10** systems is in this case the HOMO−2 orbital, while the HOMO−1, HOMO and LUMO orbitals are very similar to those of compound **10**. The π-bond between the B and S atoms can be attributed to the HOMO−2 orbital spanning over the large part of the B,N-heterocyclic ring with a partial contribution of the HOMO−1 *p*-orbital, which is highly localized on the S atom.

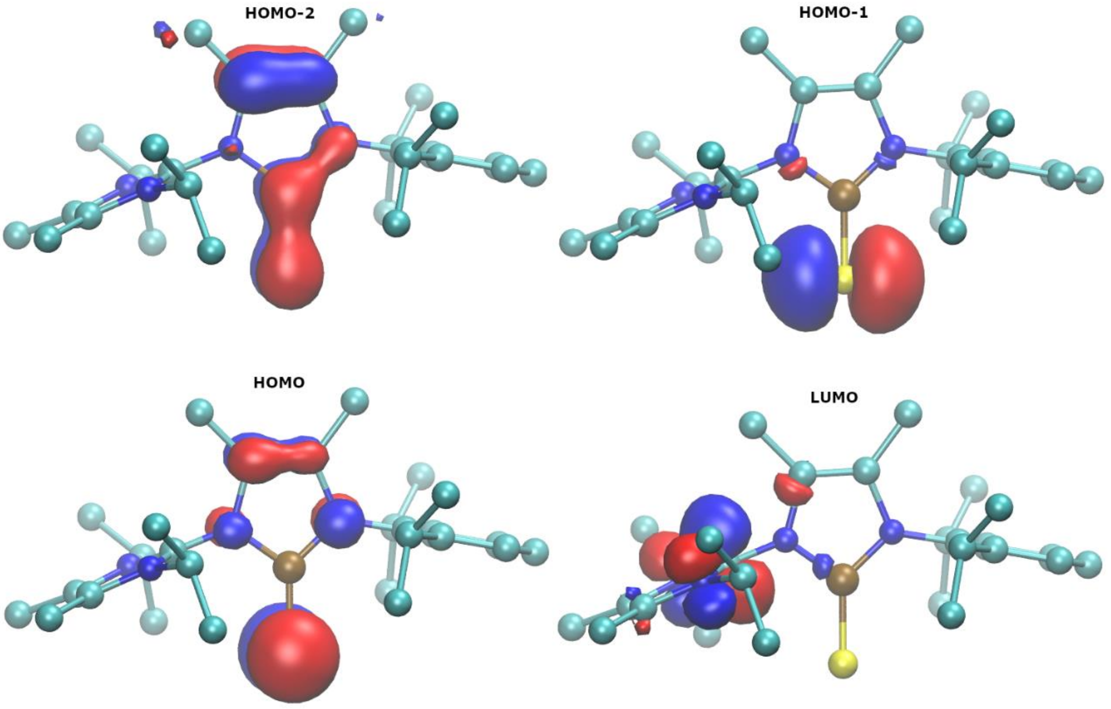

Figure S40. Selected molecular orbitals of compound **11**.

The analysis compound **12** reveals a similar pattern of orbitals close to the HOMO and LUMO as found for compound **11**. The main difference is that the HOMO−2 orbital is now antibonding with respect to the B and Se atoms, and the bonding character can be attributed to the HOMO−1 and HOMO orbitals, which both are now slightly more localized on the Se atom, and slightly less on the B atom compared to compound **10**.

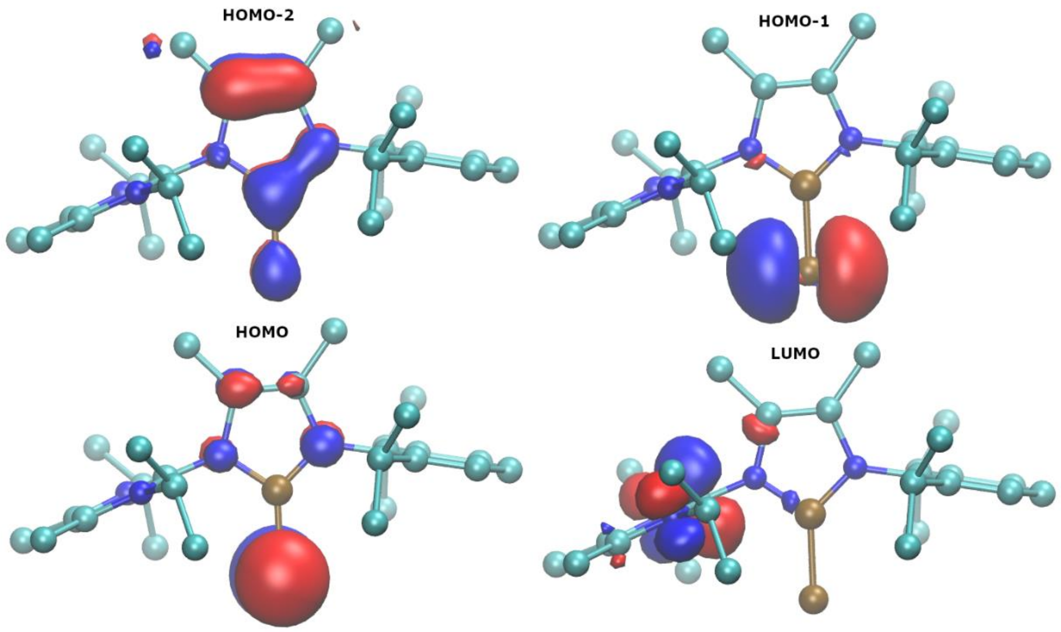

Figure S41. Selected molecular orbitals of compound **12**.

The order of the orbitals for compound **13** is similar except for the fact that HOMO−2 does not span over the Te atom, most likely due to a relatively long distance from the diazaborole ring to the Te atom. On the other hand the HOMO−1, HOMO and LUMO are again very similar and the B-Te double bond may be attributed to the HOMO−1 and HOMO orbitals, which are highly localized on the Te atom.

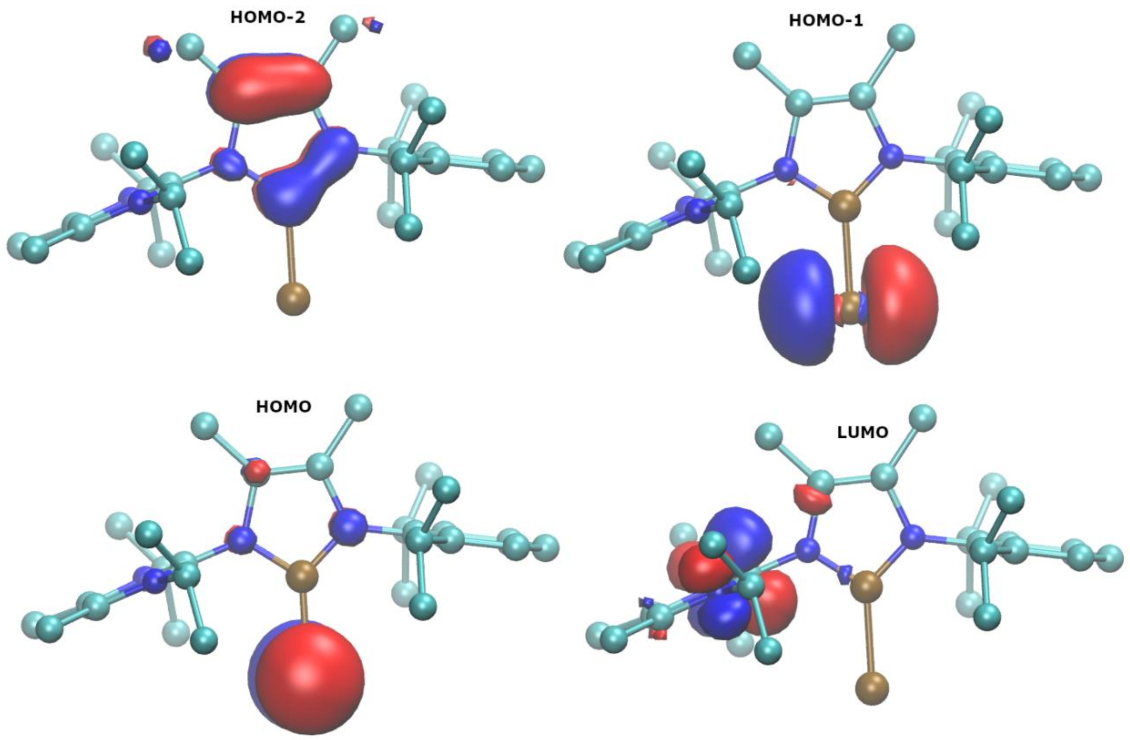

Figure S42. Selected molecular orbitals of compound **13**.

Table S8. Orbital localization of selected molecular orbitals using the Foster-Boys localization method at the ωB97XD level of theory with the 6-311++G**/WTBS basis set.

Atom	Orbital	
	**B-O HOMO−4**	**B-O HOMO−1**
B	22.4%	8.2%
O	71.9%	87.0%
	**B-S HOMO−1**	**B−S HOMO**
B	5.3%	9.5%
S	89.1%	82.1%
	**B-Se HOMO−1**	**B-Se HOMO**
B	4.8%	8.1%
Se	89.4%	83.4%
	**B-Te HOMO−1**	**B-Te HOMO**
B	3.3%	5.8%
Te	91.4%	85.7%

3.3. Cartesian coordintates for systems ***10***-***13*** *and the previously reported compounds.*

Cartesian coordinates of optimized systems at the wB97XD/6-311G++** (with WTBS on Te atom if present) level of theory.

Compound **10**

76			
O1	3.153233	16.821655	2.315123
N2	2.866975	17.006273	4.820467
N3	3.638787	19.268070	4.596792
N4	1.503991	18.996143	5.011371
N5	2.614752	14.911325	3.917108
C6	2.685959	18.346277	4.855933
C7	5.039077	18.884520	4.289204
H8	5.065113	17.824472	4.541367
C9	5.320267	19.009529	2.791749
H10	4.583837	18.413862	2.245875
H11	6.314979	18.610943	2.582644
H12	5.297668	20.049137	2.457739
C13	6.037085	19.618378	5.185212
H14	6.180312	20.655935	4.884841
H15	7.004523	19.120518	5.109176
H16	5.723626	19.592480	6.230150
C17	3.049541	20.540070	4.583994
C18	3.803162	21.804518	4.350449
H19	4.405419	22.086369	5.215869
H20	3.111192	22.620552	4.149526
H21	4.467036	21.722828	3.491497
C22	1.721805	20.372414	4.849958
C23	0.652771	21.402693	4.974230
H24	-0.177939	21.212221	4.293661
H25	1.053546	22.384995	4.731267
H26	0.252956	21.452103	5.988106
C27	0.222474	18.249119	4.954280
H28	0.490731	17.230614	5.233536
C29	-0.276909	18.216118	3.508483
H30	-0.571426	19.209229	3.162874
H31	-1.143186	17.556747	3.433873
H32	0.503915	17.832575	2.849558
C33	-0.809045	18.749716	5.962762
H34	-0.358916	18.895620	6.945586
H35	-1.594144	17.998903	6.059582
H36	-1.282060	19.679558	5.650555
C37	2.445177	16.096313	5.848176
C38	2.346797	16.546202	7.266934
H39	3.301149	16.950956	7.620838
H40	2.076811	15.716533	7.919227
H41	1.593360	17.329512	7.409668
C42	2.330431	14.866865	5.294648
C43	1.995260	13.571638	5.956245
H44	1.123673	13.110482	5.485320
H45	1.778918	13.709259	7.014770
H46	2.818131	12.857828	5.864805
C47	2.526909	13.763781	3.077978
C48	3.642105	12.915000	2.956922
C49	4.949330	13.288234	3.639404
H50	4.698652	13.859198	4.536221
C51	5.785967	12.078668	4.074834
H52	5.197589	11.382967	4.677336
H53	6.638915	12.411653	4.670578
H54	6.183466	11.532076	3.216120
C55	5.759813	14.222951	2.719194
H56	6.059054	13.690472	1.812131
H57	6.664511	14.569159	3.227157
H58	5.167381	15.089879	2.421050

C59	3.537188	11.788080	2.140131
H60	4.381543	11.120745	2.031989
C61	2.359342	11.517179	1.450464
H62	2.293245	10.640986	0.818427
C63	1.274426	12.378300	1.558720
H64	0.371864	12.171268	0.998545
C65	1.339304	13.513341	2.372009
C66	0.184120	14.500988	2.426756
H67	0.336385	15.128310	3.308579
C68	0.236941	15.420252	1.191755
H69	0.131926	14.831314	0.276145
H70	1.189758	15.953354	1.160199
H71	-0.578207	16.149397	1.220184
C72	-1.185963	13.822742	2.567620
H73	-1.963275	14.577250	2.714369
H74	-1.205298	13.139182	3.419302
H75	-1.449604	13.255275	1.671932
B76	2.946396	16.274828	3.471523

Compound **11**

76

S1	2.967476	17.041719	1.726802
N2	2.659524	17.033089	4.620312
N3	3.651898	19.227259	4.807688
N4	1.475753	19.135508	4.626148
N5	2.570142	14.935668	3.793232
C6	2.599058	18.392227	4.705630
C7	5.047808	18.722057	4.816921
H8	4.920701	17.642876	4.901731
C9	5.739020	19.000227	3.482865
H10	5.124374	18.622852	2.664522
H11	6.696342	18.476946	3.463654
H12	5.935169	20.063269	3.334266
C13	5.816600	19.218756	6.040448
H14	6.087277	20.271154	5.958902
H15	6.741544	18.648014	6.129577
H16	5.238506	19.073563	6.954316
C17	3.187641	20.544756	4.782698
C18	4.063766	21.744291	4.901304
H19	4.418197	21.888645	5.923521
H20	3.512578	22.638218	4.615016
H21	4.932734	21.676769	4.249501
C22	1.825003	20.487206	4.670864
C23	0.842869	21.607292	4.636662
H24	0.110146	21.479570	3.841705
H25	1.357851	22.548895	4.455013
H26	0.303874	21.702942	5.580938
C27	0.149742	18.514742	4.375540
H28	0.338738	17.448816	4.502070
C29	-0.287071	18.736208	2.927936
H30	-0.526397	19.781698	2.727052
H31	-1.180824	18.142212	2.731503
H32	0.500365	18.406927	2.248466
C33	-0.882483	18.944277	5.416821
H34	-0.483272	18.853256	6.428075
H35	-1.753327	18.292910	5.336473
H36	-1.222732	19.967955	5.263505
C37	2.433132	16.130783	5.710660
C38	2.327744	16.633717	7.109454
H39	3.237358	17.157724	7.422969
H40	2.168420	15.810307	7.804336
H41	1.492009	17.331348	7.233450
C42	2.394208	14.882441	5.195171
C43	2.216490	13.576065	5.892448
H44	1.322207	13.058202	5.537280
H45	2.125280	13.712759	6.969447

H46	3.064128	12.913654	5.701597
C47	2.499559	13.766084	2.977315
C48	3.682266	13.105896	2.612006
C49	5.041690	13.620513	3.057392
H50	4.865051	14.424944	3.773567
C51	5.867793	12.534869	3.765642
H52	5.319162	12.105780	4.607217
H53	6.801269	12.958400	4.144416
H54	6.126876	11.721137	3.083838
C55	5.806673	14.225463	1.867841
H56	6.017202	13.462050	1.113364
H57	6.760432	14.644066	2.201308
H58	5.218639	15.019106	1.405701
C59	3.586208	11.975347	1.796327
H60	4.487903	11.459822	1.491390
C61	2.350853	11.511828	1.362051
H62	2.293602	10.637941	0.726223
C63	1.187392	12.175306	1.736927
H64	0.230620	11.813015	1.384377
C65	1.241151	13.309685	2.548406
C66	-0.027379	14.068309	2.911781
H67	0.211750	14.724361	3.751611
C68	-0.462291	14.966343	1.739632
H69	-0.720567	14.358240	0.868380
H70	0.343867	15.644274	1.454982
H71	-1.342416	15.555482	2.015243
C72	-1.168552	13.143277	3.360498
H73	-2.014085	13.736768	3.716961
H74	-0.848966	12.481210	4.168313
H75	-1.529812	12.521344	2.538267
B76	2.749147	16.298269	3.333128

Compound **12**

76

Se1	2.971733	17.084545	1.590476
N2	2.681423	17.038900	4.594364
N3	3.649080	19.245296	4.788629
N4	1.473104	19.127888	4.618324
N5	2.591951	14.940129	3.777237
C6	2.606429	18.398617	4.681188
C7	5.051701	18.758858	4.796483
H8	4.938600	17.676056	4.847986
C9	5.756048	19.084993	3.480552
H10	5.150947	18.734230	2.643391
H11	6.713845	18.563032	3.453341
H12	5.953242	20.152388	3.370691
C13	5.798878	19.231026	6.042995
H14	6.046959	20.291298	5.997897
H15	6.735177	18.677696	6.122869
H16	5.215564	19.044283	6.945928
C17	3.167870	20.556803	4.781815
C18	4.027753	21.766602	4.914233
H19	4.381095	21.903086	5.937949
H20	3.463730	22.656334	4.640104
H21	4.896676	21.720007	4.260890
C22	1.805482	20.483105	4.676520
C23	0.809489	21.591360	4.662968
H24	0.066722	21.459124	3.878402
H25	1.310330	22.540087	4.478748
H26	0.283589	21.675638	5.615728
C27	0.149983	18.494116	4.384959
H28	0.356427	17.428675	4.484950
C29	-0.327906	18.737467	2.954232
H30	-0.598138	19.780533	2.782461
H31	-1.211381	18.125374	2.766615
H32	0.450196	18.441336	2.249061

C33	-0.861197	18.888868	5.460237
H34	-0.437905	18.777161	6.459523
H35	-1.728437	18.232201	5.384270
H36	-1.212804	19.913158	5.339608
C37	2.469275	16.140381	5.690878
C38	2.379836	16.647469	7.089343
H39	3.295561	17.166245	7.393303
H40	2.221838	15.826549	7.787450
H41	1.549745	17.350435	7.219382
C42	2.428439	14.889898	5.180632
C43	2.262046	13.585147	5.883437
H44	1.376903	13.055078	5.524279
H45	2.161543	13.726266	6.959028
H46	3.118892	12.931816	5.702461
C47	2.504384	13.769515	2.963793
C48	3.674821	13.071760	2.628750
C49	5.038969	13.550554	3.099411
H50	4.871297	14.351698	3.821389
C51	5.832223	12.440264	3.806869
H52	5.261820	12.010090	4.633288
H53	6.766763	12.841268	4.206782
H54	6.088190	11.630879	3.118841
C55	5.833854	14.149943	1.926512
H56	6.035616	13.389258	1.166963
H57	6.792696	14.542285	2.276793
H58	5.271449	14.961800	1.463523
C59	3.563076	11.940921	1.816143
H60	4.455258	11.396657	1.534234
C61	2.324261	11.513753	1.353937
H62	2.255262	10.639308	0.720079
C63	1.174141	12.214939	1.698066
H64	0.214278	11.882765	1.323886
C65	1.243510	13.350768	2.506935
C66	-0.015674	14.136907	2.842436
H67	0.247889	14.863481	3.613801
C68	-0.498902	14.927793	1.614288
H69	-0.779020	14.248651	0.804341
H70	0.290275	15.588587	1.252720
H71	-1.376006	15.530454	1.868870
C72	-1.130857	13.243612	3.408162
H73	-1.981014	13.854823	3.721402
H74	-0.782440	12.672404	4.271475
H75	-1.493499	12.534308	2.660510
B76	2.761645	16.299913	3.312582

Compound **13**

76

Te1	2.765005	17.184420	1.293162
N2	2.526083	17.056627	4.521722
N3	3.632097	19.194202	4.760119
N4	1.451402	19.220064	4.605376
N5	2.496049	14.949941	3.731024
C6	2.536427	18.419931	4.629741
C7	5.003767	18.623631	4.746220
H8	4.828279	17.547972	4.742722
C9	5.737719	18.973604	3.452828
H10	5.112918	18.725220	2.594035
H11	6.649953	18.378159	3.392738
H12	6.021524	20.025866	3.409415
C13	5.769991	18.987461	6.017299
H14	6.073083	20.034269	6.028352
H15	6.675550	18.382093	6.067869
H16	5.175178	18.784445	6.908997
C17	3.234753	20.532267	4.804470
C18	4.169323	21.680359	4.974315
H19	4.534898	21.757057	5.999940

H20	3.660690	22.612795	4.736477
H21	5.030242	21.603954	4.313598
C22	1.869721	20.548407	4.707766
C23	0.947865	21.718504	4.745571
H24	0.186773	21.660929	3.970305
H25	1.506854	22.638065	4.582250
H26	0.443815	21.806532	5.709734
C27	0.082942	18.681660	4.392420
H28	0.230261	17.602028	4.417397
C29	-0.444524	19.041506	3.004461
H30	-0.693764	20.099888	2.917978
H31	-1.351708	18.466856	2.811709
H32	0.293790	18.774763	2.247140
C33	-0.852632	19.070263	5.536362
H34	-0.400023	18.859266	6.506263
H35	-1.769436	18.485540	5.455144
H36	-1.129789	20.123644	5.499983
C37	2.402731	16.163397	5.636267
C38	2.327074	16.681604	7.031417
H39	3.221588	17.250877	7.305459
H40	2.233463	15.860986	7.741167
H41	1.464653	17.340385	7.177951
C42	2.388157	14.906681	5.140871
C43	2.285332	13.605610	5.861989
H44	1.411192	13.040954	5.530636
H45	2.205449	13.756945	6.937857
H46	3.159588	12.979940	5.669086
C47	2.485881	13.758460	2.940032
C48	3.701150	13.121258	2.643351
C49	5.030252	13.687491	3.119607
H50	4.810350	14.516973	3.794280
C51	5.853244	12.651849	3.902857
H52	5.284271	12.242034	4.740378
H53	6.761782	13.112684	4.298208
H54	6.156164	11.818530	3.264375
C55	5.830449	14.259015	1.936513
H56	6.089085	13.469358	1.225647
H57	6.759974	14.714203	2.289871
H58	5.247203	15.017588	1.413366
C59	3.668521	11.963287	1.863298
H60	4.595287	11.463729	1.611740
C61	2.464436	11.452565	1.393643
H62	2.456800	10.557929	0.784892
C63	1.271103	12.096965	1.696964
H64	0.338158	11.701129	1.316508
C65	1.259886	13.257551	2.473584
C66	-0.052164	13.969558	2.767327
H67	0.165033	14.784786	3.460105
C68	-0.627384	14.598112	1.486595
H69	-0.875763	13.826556	0.752649
H70	0.096653	15.280572	1.040108
H71	-1.540659	15.155734	1.713066
C72	-1.074334	13.040995	3.442957
H73	-1.975051	13.600052	3.708308
H74	-0.666699	12.595825	4.353285
H75	-1.373647	12.227708	2.777511
B76	2.590320	16.306839	3.253974

Cowley neutral B=O

42

C	8.544594	2.771318	1.191427
C	7.182562	3.382104	1.184765
C	6.240369	3.062859	0.212080
C	4.964404	3.619958	0.193961
C	3.963707	3.263891	-0.854330
C	3.328582	5.118845	1.204553

C	3.125635	6.373963	0.648813
C	1.928931	7.042738	0.821273
C	0.920019	6.450911	1.564180
C	1.098846	5.192093	2.115044
C	2.301877	4.537354	1.934279
C	7.772433	4.641951	3.185657
C	7.815284	3.941601	4.381739
C	8.611609	4.364698	5.428409
C	9.372524	5.512731	5.284929
C	9.343253	6.226248	4.098533
C	8.543145	5.788248	3.060904
B	5.537970	4.912647	2.228132
F	4.139127	6.947567	-0.074822
F	1.739033	8.274451	0.268520
F	-0.264289	7.103051	1.737546
F	0.095402	4.609798	2.831708
F	2.500654	3.294108	2.478450
F	7.052006	2.807549	4.512543
F	8.647766	3.659880	6.597277
F	10.158521	5.938197	6.313027
F	10.098244	7.355021	3.959226
F	8.499884	6.490543	1.882512
Al	4.586923	6.871561	4.377936
Cl	6.248377	7.156245	5.791360
Cl	3.986405	8.675105	3.280967
Cl	2.827125	5.865090	5.224632
O	5.215322	5.759568	3.159571
N	4.621416	4.507469	1.155788
N	6.854217	4.269278	2.154153
H	6.507755	2.357021	-0.555651
H	9.312404	3.545710	1.146793
H	8.674925	2.103911	0.344782
H	4.382471	2.562409	-1.569701
H	8.701487	2.202148	2.109708
H	3.637262	4.156937	-1.390211
H	3.081219	2.811126	-0.398265

Cui neutral B=O

108

B	6.886578	7.847994	10.968885
B	8.649926	8.302809	8.866345
N	5.889118	6.752266	11.038531
N	6.922175	8.673757	12.202507
O	7.563537	8.089871	9.871543
C	4.024871	5.527721	12.119227
H	3.271250	5.884808	11.414362
H	3.572163	5.442908	13.103663
H	4.317259	4.541408	11.774825
C	5.169014	6.494797	12.155167
C	5.448749	7.184992	13.326577
H	4.924440	6.919579	14.228868
C	6.307823	8.272320	13.344196
C	6.528880	9.015125	14.625609
H	7.499112	8.731562	15.036970
H	5.763459	8.755238	15.352365
H	6.537884	10.093251	14.485217
C	5.612757	5.923667	9.873584
C	6.056504	4.591274	9.877869
C	5.622179	3.766315	8.838886
H	5.948241	2.735686	8.808990
C	4.817703	4.258441	7.822279
H	4.506308	3.608765	7.016901
C	4.452027	5.599548	7.807185
H	3.869120	5.977533	6.980317
C	4.840758	6.464493	8.832011
C	7.044538	4.040958	10.905084

H	7.341501	4.852045	11.570281
C	8.323346	3.542722	10.203786
H	9.095029	3.333939	10.946367
H	8.700806	4.279441	9.500629
H	8.130793	2.621503	9.649374
C	6.470302	2.899842	11.765174
H	5.666247	3.227194	12.424570
H	7.259351	2.484813	12.395045
H	6.087005	2.092586	11.136909
C	4.450292	7.940278	8.807734
H	5.298022	8.507077	9.193292
C	3.225717	8.228189	9.695453
H	2.377970	7.608140	9.393723
H	2.930749	9.274816	9.597673
H	3.429622	8.045521	10.752023
C	4.208155	8.477534	7.391224
H	5.029145	8.215332	6.724727
H	4.141207	9.565706	7.423308
H	3.278232	8.097957	6.961337
C	7.484006	10.020974	12.182176
C	6.606483	11.066701	11.819367
C	7.095854	12.370773	11.882627
H	6.457853	13.194175	11.601521
C	8.410341	12.627594	12.258474
H	8.778419	13.644289	12.267978
C	9.248899	11.582217	12.612268
H	10.266636	11.792443	12.911732
C	8.795922	10.257691	12.613076
C	5.158084	10.811312	11.403058
H	5.121470	9.839476	10.908403
C	4.211013	10.761861	12.618826
H	4.433651	9.942257	13.299919
H	3.181387	10.634542	12.278111
H	4.269112	11.697808	13.179672
C	4.618277	11.844987	10.402748
H	4.437642	12.808989	10.883866
H	3.660450	11.498982	10.009010
H	5.297180	11.994779	9.569103
C	9.715111	9.164279	13.139271
H	9.166379	8.221903	13.135528
C	10.167428	9.458998	14.583188
H	10.691944	8.593347	14.992014
H	9.332218	9.700184	15.241975
H	10.857104	10.305144	14.608487
C	10.949118	8.976519	12.247827
H	10.669723	8.759930	11.223857
H	11.556386	8.147141	12.615977
H	11.571937	9.874257	12.237340
C	9.863367	7.230278	9.143246
C	10.029642	6.472987	10.292248
C	11.073126	5.583666	10.488542
C	12.003715	5.393387	9.489450
C	11.884576	6.118353	8.316464
C	10.835911	7.004974	8.172097
C	8.032900	7.986649	7.357592
C	7.677608	6.679721	7.036823
C	7.161614	6.279279	5.818074
C	6.965230	7.220250	4.827419
C	7.327867	8.529333	5.072413
C	7.858383	8.882160	6.304120
C	9.154247	9.861250	8.999126
C	10.452410	10.333026	9.136959
C	10.773079	11.677148	9.263344
C	9.774705	12.624549	9.205543
C	8.468166	12.211045	9.027628
C	8.191689	10.863885	8.954944
F	9.142317	6.565398	11.367037
F	11.168873	4.874033	11.665065

F	13.036379	4.505806	9.659054
F	12.810827	5.951096	7.317132
F	10.796160	7.728132	6.992989
F	7.892110	5.667698	7.956070
F	6.847120	4.962052	5.579137
F	6.432929	6.856436	3.615904
F	7.160755	9.474250	4.088774
F	8.240605	10.208223	6.402594
F	11.536495	9.469418	9.184338
F	12.075451	12.067424	9.470356
F	10.062119	13.957948	9.365614
F	7.462022	13.148420	8.958062
F	6.862111	10.516252	8.760147

Kinjo neutral B=O

64

Al	2.828470	1.720735	3.662546
B	1.974390	4.269302	5.119063
C	3.624078	5.754189	2.907765
H	3.059762	4.908821	2.541571
C	4.615671	6.331058	2.122414
H	4.815285	5.918832	1.143952
C	5.338878	7.429342	2.586187
H	6.103044	7.876692	1.966813
C	5.082313	7.939437	3.856097
H	5.651753	8.778691	4.230513
C	4.111537	7.353330	4.664953
H	3.968453	7.713651	5.676282
C	3.377169	6.268879	4.181545
C	0.796321	5.637806	6.585404
C	-0.125410	6.226714	7.637102
C	0.092484	7.749992	7.732726
H	1.114764	7.999898	8.027098
H	-0.569428	8.147984	8.500730
H	-0.158307	8.260064	6.798868
C	-1.597844	5.953580	7.266410
H	-1.844778	6.352015	6.281277
H	-2.239608	6.439217	8.002808
H	-1.815564	4.888143	7.276789
C	0.186116	5.588880	9.007891
H	-0.041378	4.525378	9.006391
H	-0.433205	6.068684	9.766900
H	1.233042	5.722769	9.284119
C	0.226743	3.188916	6.646018
C	-0.934375	2.798951	5.958418
C	-1.478496	3.578817	4.767626
H	-1.064002	4.589712	4.800384
C	-3.009684	3.713009	4.790708
H	-3.494354	2.753442	4.602538
H	-3.331817	4.397789	4.003878
H	-3.373803	4.092479	5.747039
C	-1.017044	2.935437	3.446768
H	0.067056	2.929194	3.360466
H	-1.421779	3.484492	2.594314
H	-1.353294	1.899234	3.384422
C	-1.552060	1.613642	6.358638
H	-2.437739	1.274754	5.840904
C	-1.023581	0.842066	7.384427
H	-1.505138	-0.084626	7.663495
C	0.139802	1.237946	8.029533
H	0.559852	0.609979	8.802115
C	0.797555	2.414850	7.668741
C	2.124726	2.778241	8.322294
H	2.332662	3.831278	8.115291
C	2.105245	2.608240	9.850030
H	1.258953	3.122043	10.309320

```
H        3.025272    3.011252   10.277772
H        2.052298    1.554991   10.131096
C        3.267991    1.951944    7.704179
H        3.088991    0.884172    7.842452
H        4.219951    2.206343    8.174112
H        3.360800    2.132745    6.635836
Cl       1.499448    0.137442    4.422920
Cl       4.943030    1.305204    4.086448
Cl       2.484335    2.162166    1.522418
N        2.382195    5.646134    4.978898
N        1.645793    6.395195    5.890939
H        1.710120    7.396274    5.902902
N        0.938286    4.360102    6.184261
O        2.389900    3.201046    4.495317
```

Rivard neutral B=O

```
102
Cl       7.037125    5.659092   14.637034
F        3.858655    6.300961   14.127839
F        4.006465    8.850300   15.033345
F        4.200317    9.357350   17.743482
F        4.228067    7.255687   19.523361
F        4.069056    4.710989   18.656482
F        1.366353    4.402129   16.966398
F       -1.086555    4.332236   15.810851
F       -1.354623    3.618595   13.156094
F        0.897930    2.986067   11.685211
F        3.320947    3.076316   12.773160
F        2.089392    1.546826   16.564505
F        2.868776   -0.429300   18.193825
F        5.397328   -0.441704   19.300628
F        7.145801    1.606908   18.718237
F        6.412373    3.599358   17.048155
O        4.983021    3.764769   14.647302
N        6.842888    2.098815   12.809504
N        6.889488    3.892877   11.596738
C        6.608022    3.428569   12.832959
C        7.260346    1.720314   11.541622
H        7.508801    0.702333   11.317121
C        7.277250    2.841165   10.779235
H        7.525942    2.998392    9.748811
C        6.814003    1.211393   13.953248
C        5.758980    0.298916   14.070810
C        5.801190   -0.586521   15.151274
H        5.001954   -1.302284   15.282624
C        6.851268   -0.555797   16.058880
H        6.864646   -1.246646   16.890880
C        7.878819    0.371182   15.918560
H        8.671165    0.397060   16.650441
C        7.883127    1.283694   14.863761
C        4.628024    0.227090   13.057698
H        4.642944    1.139702   12.462563
C        3.248517    0.160310   13.726607
H        2.468042    0.274144   12.972381
H        3.085489   -0.794002   14.231989
H        3.126700    0.954031   14.458756
C        4.826776   -0.971070   12.110912
H        4.032232   -0.995517   11.363065
H        5.784670   -0.924689   11.588622
H        4.798087   -1.910129   12.668425
C        9.032829    2.270684   14.692280
H        8.660222    3.153185   14.169891
C        9.598742    2.774910   16.026270
H        8.805813    3.137103   16.678393
H       10.151766    1.993932   16.552155
H       10.290904    3.597680   15.839594
```

```
C       10.141810    1.656557   13.817193
H        9.765503    1.360836   12.835663
H       10.947616    2.378391   13.668374
H       10.560659    0.771340   14.300731
C        6.862819    5.280559   11.184958
C        5.665608    5.813640   10.690887
C        5.682749    7.156518   10.306215
H        4.778243    7.611266    9.929777
C        6.842353    7.913553   10.408142
H        6.831583    8.953255   10.111552
C        8.017051    7.347680   10.892425
H        8.908290    7.953514   10.968101
C        8.054982    6.013611   11.296343
C        4.418852    4.961302   10.516234
H        4.495605    4.097316   11.174546
C        3.126290    5.691150   10.904758
H        2.294529    4.986612   10.876708
H        3.193670    6.106289   11.908965
H        2.892111    6.500463   10.209542
C        4.335558    4.437353    9.069923
H        5.224887    3.864917    8.796212
H        3.464702    3.789411    8.957676
H        4.239701    5.266935    8.365340
C        9.345847    5.383010   11.803414
H        9.085837    4.506917   12.402571
C       10.155061    6.317230   12.714355
H       10.980137    5.763985   13.167209
H       10.589256    7.148154   12.154517
H        9.536214    6.723700   13.512883
C       10.210404    4.901833   10.622497
H       11.117645    4.417654   10.989123
H        9.675852    4.189117    9.992378
H       10.503318    5.748110    9.997121
C        3.986123    5.396899   16.348666
C        3.953817    6.499245   15.502780
C        4.026142    7.810031   15.931525
C        4.124845    8.065904   17.286442
C        4.141938    7.007661   18.175110
C        4.068727    5.708377   17.699145
C        2.459328    3.773652   14.933259
C        1.289207    4.074794   15.624663
C        0.026462    4.029737   15.066581
C       -0.112980    3.671446   13.737241
C        1.014816    3.356139   13.008470
C        2.263408    3.420321   13.606130
C        4.217514    2.680002   16.734547
C        3.370133    1.637734   17.085238
C        3.737155    0.604311   17.934875
C        4.995502    0.598549   18.498300
C        5.873467    1.621456   18.194882
C        5.476928    2.612555   17.323183
B        6.053155    4.222223   14.086368
B        3.901097    3.898787   15.692085
```

Cui anionic B=O

```
107
B        4.836925    8.240474    6.623679
O        5.925091    8.813451    6.126100
N        4.546461    8.168507    8.057336
N        3.808400    7.619626    5.772351
C        3.035010    7.760678    9.923894
C        3.340387    7.679716    8.607659
C        2.453214    7.033189    7.650101
C        2.682960    6.994258    6.320914
C        1.749323    6.279394    5.382297
C        5.524913    8.650746    8.982713
```

C	5.600161	10.019953	9.275580
C	6.514970	10.446972	10.244509
H	6.577295	11.499636	10.491839
C	7.324758	9.535853	10.910381
H	8.004082	9.877085	11.681912
C	7.260151	8.181599	10.590082
H	7.895935	7.480477	11.114234
C	6.361190	7.718500	9.627707
C	4.710199	11.025424	8.564669
H	4.029574	10.460319	7.927955
C	3.854353	11.833953	9.551275
H	3.259997	11.165593	10.175715
H	3.172775	12.492615	9.007375
H	4.471773	12.457562	10.203748
C	5.545663	11.938185	7.652736
H	6.254061	12.536975	8.233733
H	4.899120	12.622412	7.096839
H	6.098546	11.327608	6.938367
C	6.276953	6.246728	9.251081
H	5.251169	6.058700	8.934662
C	7.191650	5.954499	8.047559
H	7.018725	6.674691	7.246464
H	7.005878	4.950204	7.657819
H	8.242449	6.012822	8.345320
C	6.574073	5.296006	10.416222
H	7.621604	5.343106	10.727135
H	6.370815	4.266397	10.113741
H	5.947071	5.528010	11.278757
C	3.949822	7.676648	4.347466
C	3.236047	8.656449	3.630300
C	3.344024	8.678538	2.239048
H	2.793122	9.413852	1.668089
C	4.152203	7.761195	1.572681
H	4.222161	7.786225	0.492611
C	4.873367	6.819907	2.295159
H	5.512096	6.119166	1.772034
C	4.783151	6.759521	3.689673
C	2.413931	9.695629	4.380997
H	2.062195	9.236071	5.306281
C	1.180030	10.181482	3.610621
H	1.455579	10.775011	2.734999
H	0.567771	10.816067	4.254721
H	0.565995	9.343578	3.273806
C	3.315252	10.878101	4.782583
H	4.196244	10.524245	5.320141
H	2.771372	11.578534	5.421541
H	3.649266	11.415015	3.889778
C	5.600081	5.738510	4.465562
H	5.284944	5.794261	5.508293
C	7.094472	6.099487	4.417331
H	7.678150	5.389228	5.008596
H	7.237809	7.099350	4.827932
H	7.468824	6.076426	3.389215
C	5.352678	4.302052	3.979849
H	4.289916	4.053917	4.011744
H	5.887965	3.592879	4.615685
H	5.702031	4.158098	2.954132
N	9.302817	10.157155	6.396842
N	8.537471	10.663651	4.434716
C	8.224981	10.077715	5.600186
H	7.248805	9.575844	5.867477
C	10.345585	10.801647	5.714374
C	9.853387	11.129139	4.481532
C	11.705818	11.057176	6.269481
H	12.394306	11.300749	5.461552
H	12.105609	10.183663	6.782664
H	11.711027	11.891406	6.972341
C	10.493455	11.836691	3.338313

```
H        11.506494   12.138134    3.597722
H         9.942880   12.739568    3.066368
H        10.553427   11.201066    2.452452
C         9.286574    9.593388    7.771656
H         8.231625    9.388358    7.967079
C         9.774715   10.611876    8.798054
H        10.848352   10.784181    8.733938
H         9.553989   10.230077    9.791862
H         9.248894   11.559991    8.682498
C        10.048126    8.271122    7.821141
H         9.659342    7.579135    7.074787
H         9.910149    7.820328    8.804245
H        11.118114    8.409051    7.653495
C         7.577774   10.868015    3.323476
H         8.187952   11.124390    2.456333
C         6.662748   12.044691    3.665443
H         6.064392   11.807466    4.544968
H         5.983410   12.241222    2.835505
H         7.239805   12.949430    3.866307
C         6.810200    9.582780    3.037875
H         7.490545    8.772929    2.772445
H         6.125207    9.740041    2.205487
H         6.224380    9.267723    3.904429
H         1.572921    6.550075    8.047958
H         2.102443    7.349499   10.282971
H         3.690137    8.220076   10.648109
H         0.956716    5.788549    5.943971
H         1.291271    6.965918    4.666692
H         2.282977    5.525456    4.799106
```

Aldridge anionic B=O

```
66
O         0.670547    8.538561    2.475101
N        -0.277634    6.541799    1.215905
N         1.925600    7.055688    0.840989
C         0.272376    5.628218    0.297374
H        -0.320894    4.846297   -0.148155
C         1.568238    5.936528    0.072002
H         2.271666    5.454233   -0.587013
C        -1.630993    6.489421    1.618138
C        -2.083202    5.408400    2.403067
C        -3.438224    5.337117    2.736383
H        -3.806120    4.510240    3.329960
C        -4.321203    6.334412    2.336407
H        -5.369535    6.269579    2.602890
C        -3.852077    7.430535    1.618819
H        -4.543059    8.216930    1.345939
C        -2.507706    7.532783    1.251421
C        -1.963281    8.736883    0.492493
H        -1.034456    9.012884    1.000319
C        -1.633750    8.381583   -0.968949
H        -2.544811    8.111776   -1.514409
H        -0.935128    7.547100   -1.022003
H        -1.175312    9.239474   -1.470096
C        -2.880889    9.964101    0.542067
H        -2.365581   10.817760    0.094922
H        -3.135584   10.226173    1.571015
H        -3.809491    9.807165   -0.017751
C        -1.063411    4.428537    2.970894
H        -0.317202    4.232301    2.199247
C        -1.657596    3.079155    3.392932
H        -2.320496    3.183187    4.256839
H        -0.854057    2.395428    3.678507
H        -2.225900    2.619903    2.580132
C        -0.324406    5.093893    4.149881
H         0.101715    6.052362    3.851507
```

H	0.483603	4.449164	4.508718
H	-1.019107	5.272434	4.976360
C	3.203669	7.659736	0.764426
C	4.232011	7.218954	1.618356
C	5.489676	7.819602	1.523047
H	6.292655	7.494393	2.172408
C	5.720032	8.846187	0.611952
H	6.699900	9.304748	0.549609
C	4.687523	9.298761	-0.202855
H	4.869058	10.117780	-0.887642
C	3.417575	8.718657	-0.137595
C	2.249042	9.264443	-0.943476
H	1.521245	8.456383	-1.037958
C	1.567353	10.400418	-0.155282
H	2.250916	11.249978	-0.050237
H	1.278474	10.053564	0.841139
H	0.670641	10.743297	-0.681711
C	2.635242	9.718234	-2.357528
H	1.734835	9.979494	-2.919826
H	3.161787	8.928219	-2.898691
H	3.276478	10.604487	-2.337301
C	3.913094	6.180582	2.682316
H	3.115570	5.549546	2.284748
C	3.350927	6.887798	3.932132
H	3.023237	6.150773	4.671822
H	2.501209	7.524326	3.670654
H	4.125053	7.514342	4.388003
C	5.099655	5.276939	3.041708
H	5.887015	5.831730	3.560521
H	5.536085	4.817822	2.151040
H	4.767652	4.479972	3.712205
B	0.761050	7.532026	1.646368

Aldridge anionic B=S

66

C	4.908763	11.128324	11.337128
H	4.427881	10.186723	11.133328
N	4.189926	12.227994	11.835954
S	4.699056	15.029626	12.624174
B	5.075001	13.383210	12.007093
C	2.826915	12.114324	12.220320
N	6.359422	12.828127	11.563698
C	6.199854	11.486023	11.172755
H	7.029132	10.904427	10.806954
C	1.815973	12.320940	11.265531
C	0.483589	12.194366	11.664958
H	-0.308986	12.361388	10.946256
C	0.160309	11.873906	12.979758
H	-0.877935	11.784119	13.275855
C	2.514952	11.803978	13.554666
C	1.170221	11.685333	13.915940
H	0.913730	11.455734	14.943403
C	3.610618	11.635009	14.596364
H	4.566076	11.735669	14.080715
C	3.544262	12.756132	15.645271
H	3.631127	13.725076	15.151740
H	4.367735	12.656740	16.358170
H	2.602510	12.720347	16.202859
C	3.573471	10.242521	15.245681
H	2.646290	10.087345	15.805738
H	4.409000	10.129813	15.942124
H	3.648828	9.458374	14.488856
C	2.177571	12.752027	9.852734
H	3.222970	12.484277	9.692065
C	2.078486	14.283679	9.733630
H	2.406365	14.611733	8.742474

```
H        2.705258   14.764519   10.487368
H        1.044469   14.612072    9.881628
C        1.340638   12.049360    8.773924
H        1.718435   12.310249    7.781807
H        0.290793   12.353435    8.814754
H        1.384423   10.963187    8.884304
C        7.644456   13.433253   11.630511
C        8.022936   14.372680   10.654874
C        9.304030   14.924798   10.727071
H        9.616667   15.652098    9.989817
C       10.182753   14.563911   11.743317
H       11.172312   15.003140   11.784263
C        9.784355   13.656514   12.718184
H       10.465384   13.403940   13.520681
C        8.017450   12.179754   13.806213
H        7.257041   11.517833   13.388635
C        8.509666   13.084691   12.684056
C        7.332268   13.025923   14.895958
H        8.059585   13.688700   15.375172
H        6.896450   12.377723   15.662770
H        6.539900   13.645098   14.470744
C        9.116604   11.292473   14.406958
H        9.644127   10.732047   13.631261
H        8.673504   10.578726   15.106221
H        9.852106   11.879884   14.963774
C        7.042983   14.753555    9.554851
H        6.051365   14.749430   10.009252
C        7.057693   13.709555    8.423958
H        6.324396   13.971179    7.655659
H        6.807366   12.719770    8.807568
H        8.046132   13.662857    7.954861
C        7.268694   16.163898    8.998156
H        8.199384   16.238395    8.425934
H        7.292733   16.896652    9.806119
H        6.447337   16.426818    8.327282
```

Inoue cationic B=S

```
104
S       15.462345   12.438124    5.901269
B       15.568816   10.785980    5.329226
N       16.375877    9.663504    5.854743
N       14.873816   10.142471    4.192808
N       18.475519    9.460435    7.028489
N       16.718369    9.913029    8.240955
N       14.413779   11.770135    2.457643
N       12.767929   10.468926    3.058261
C       16.376996    8.508420    4.931183
H       16.438467    7.563913    5.469998
H       17.211712    8.567234    4.229628
C       15.035863    8.672512    4.212373
H       15.058720    8.257827    3.205504
H       14.227002    8.195624    4.769813
C       17.141110    9.690266    6.972936
C       18.901289    9.548221    8.352173
H       19.929435    9.403829    8.619416
C       17.812583    9.839919    9.101295
H       17.701288    9.995383   10.155802
C       19.300984    9.225986    5.871926
C       19.616452   10.305721    5.039303
C       20.358199   10.028208    3.888751
H       20.617785   10.849295    3.232763
C       20.777921    8.736151    3.571146
C       20.446985    7.691363    4.439922
H       20.771066    6.684582    4.208103
C       19.708868    7.913286    5.600550
C       19.171046   11.709374    5.343646
```

H	19.818638	12.429121	4.844849
H	19.186733	11.922910	6.413001
H	18.150261	11.894533	5.001569
C	21.600951	8.474431	2.336237
H	21.375036	7.498488	1.905762
H	22.667189	8.489329	2.574604
H	21.424847	9.231714	1.572062
C	19.358999	6.776151	6.527179
H	19.536424	5.817025	6.043771
H	18.312497	6.808803	6.838226
H	19.963627	6.802602	7.436596
C	15.348790	10.119978	8.642827
C	14.434440	9.075293	8.481431
C	13.101821	9.320924	8.818375
H	12.380491	8.522331	8.696053
C	12.685485	10.553938	9.313934
C	13.642209	11.555184	9.498430
H	13.334953	12.516943	9.888700
C	14.982180	11.365300	9.172917
C	14.823877	7.711582	7.970530
H	14.276282	6.937002	8.507034
H	15.887871	7.506777	8.095184
H	14.578992	7.610421	6.911802
C	11.240684	10.807762	9.658872
H	10.602920	9.982413	9.340831
H	10.878344	11.720471	9.181850
H	11.110333	10.929937	10.736182
C	15.986071	12.467735	9.378586
H	16.643021	12.561408	8.515109
H	16.592475	12.297739	10.272645
H	15.477717	13.422087	9.501077
C	14.068273	10.765566	3.298820
C	13.303022	12.120043	1.691636
H	13.355331	12.904646	0.963501
C	12.284567	11.306332	2.055037
H	11.270476	11.241612	1.713295
C	15.734463	12.333875	2.324317
C	16.766705	11.522972	1.844222
C	18.048869	12.072307	1.782614
H	18.860036	11.455699	1.415567
C	18.303521	13.385995	2.168433
C	17.233268	14.172432	2.601850
H	17.414368	15.197461	2.898882
C	15.936867	13.672775	2.687878
C	16.552697	10.100845	1.392631
H	15.517290	9.904011	1.111495
H	16.830746	9.398805	2.180565
H	17.175395	9.881837	0.525590
C	19.695919	13.959308	2.110455
H	20.434992	13.191506	1.879467
H	19.970117	14.419596	3.061664
H	19.770429	14.731483	1.341865
C	14.807088	14.545863	3.163931
H	15.198310	15.421320	3.678428
H	14.174823	14.011913	3.871730
H	14.192639	14.897697	2.330457
C	12.021076	9.477072	3.787866
C	11.616085	9.766567	5.095966
C	10.960064	8.753771	5.799076
H	10.633919	8.957114	6.811093
C	10.707385	7.503121	5.234197
C	11.121436	7.265315	3.919882
H	10.926744	6.301027	3.467574
C	11.780394	8.240788	3.174387
C	11.881433	11.099547	5.739458
H	11.773383	11.924302	5.033936
H	12.894629	11.162104	6.143023
H	11.190223	11.266646	6.564313

C	9.972276	6.441380	6.010862
H	10.311818	5.441488	5.739581
H	8.899660	6.490342	5.808290
H	10.109319	6.566818	7.085131
C	12.222165	7.968789	1.758428
H	13.243772	8.309270	1.575749
H	11.581251	8.479364	1.036034
H	12.178476	6.903328	1.539373

Cui neutral B=S

50

B	3.293903	2.623313	0.916864
S	3.359785	1.776663	-0.613321
N	2.037759	3.113296	1.567497
N	4.485713	2.959253	1.758328
C	0.687990	4.211222	3.311155
H	0.181072	4.889472	2.623413
H	0.833649	4.717077	4.262454
H	0.019532	3.362139	3.460368
C	2.003698	3.762938	2.747175
C	3.185076	4.015751	3.436720
H	3.144350	4.536230	4.378985
C	4.417947	3.611422	2.935181
C	5.676411	3.898636	3.699287
H	6.203445	2.974869	3.941882
H	5.449904	4.428285	4.621374
H	6.363302	4.501856	3.104032
C	0.784446	2.869940	0.878367
C	0.089970	1.687758	1.147708
C	-1.131584	1.488643	0.502324
H	-1.684178	0.577060	0.688402
C	-1.629246	2.436470	-0.385732
H	-2.574742	2.265796	-0.882480
C	-0.906209	3.594887	-0.650305
H	-1.283755	4.319598	-1.359681
C	0.319197	3.829562	-0.024466
C	0.675353	0.646036	2.063550
H	-0.014238	-0.187219	2.194078
H	0.910315	1.047460	3.052997
H	1.603942	0.260120	1.636375
C	1.146383	5.046011	-0.344868
H	1.402151	5.621712	0.548703
H	0.615693	5.706515	-1.029703
H	2.082577	4.740499	-0.818081
C	5.790126	2.553926	1.269314
C	6.280904	1.297013	1.632159
C	7.553003	0.939868	1.182010
H	7.951562	-0.031686	1.443156
C	8.296869	1.808530	0.390330
H	9.278950	1.515663	0.044509
C	7.774293	3.045407	0.027356
H	8.344779	3.709632	-0.608505
C	6.505942	3.438166	0.458002
C	5.434812	0.345929	2.436019
H	5.986668	-0.564923	2.665579
H	4.543920	0.073559	1.865176
H	5.099450	0.783957	3.379906
C	5.897046	4.744998	0.024344
H	5.013937	4.553792	-0.589886
H	6.605285	5.326277	-0.565064
H	5.581144	5.358018	0.872715

Braunschweig neutral B=S

```
30
S        3.138952    3.519824    3.321982
B        4.755125    2.862243    3.106795
C        5.912749    3.833716    3.635605
N        6.514011    4.836427    2.948431
C        5.225889    1.468965    2.475269
N        6.399754    3.911340    4.898914
C        6.023723    0.665021    3.530487
H        6.352129   -0.290886    3.109003
H        5.407533    0.448493    4.407118
H        6.919166    1.203777    3.858649
C        6.158216    1.735736    1.268579
H        6.487428    0.788633    0.828237
H        7.055793    2.289434    1.564398
H        5.640487    2.302756    0.490454
C        4.031006    0.625969    2.005113
H        4.374332   -0.322794    1.576632
H        3.449860    1.160728    1.252299
H        3.355521    0.411880    2.834758
C        7.375555    5.543515    3.782269
H        7.941041    6.385510    3.433567
C        7.304071    4.964441    5.003273
H        7.794968    5.203249    5.926477
C        6.236245    5.172535    1.555486
H        6.940183    4.674523    0.891717
H        6.306880    6.250273    1.430371
H        5.223063    4.857477    1.318126
C        5.975798    3.061424    6.007174
H        5.979169    3.646482    6.923639
H        6.638825    2.204801    6.110728
H        4.962179    2.719041    5.813650
```

Braunschweig neutral Mn B=S

```
45
Mn       6.489238    8.604581    8.850635
O        9.285944    9.028765    9.602175
C        8.155855    8.785025    9.342692
S        5.761532    7.001646   10.555193
B        6.760183    5.579380   10.887014
C        6.868651    7.481146    7.566882
O        7.115350    6.829068    6.609021
C        6.436044    4.445109   11.959147
C        6.332156    3.078159   11.237700
H        6.128836    2.287014   11.966102
H        7.261824    2.819733   10.721371
H        5.518229    3.076604   10.508602
C        7.597184    4.360758   12.981008
H        7.395123    3.569728   13.709872
H        7.709475    5.298557   13.530490
H        8.549504    4.125994   12.495608
C        5.124990    4.707971   12.717327
H        4.938395    3.908284   13.442052
H        4.277907    4.758594   12.031464
H        5.164465    5.658101   13.252372
C        4.580987    9.312780    8.049864
H        3.839230    8.709051    7.559940
C        4.614954    9.645777    9.426599
H        3.940998    9.269277   10.175244
C        5.731501   10.482966    9.673887
H        6.006225   10.913934   10.619106
C        6.396662   10.692783    8.419104
H        7.256949   11.317643    8.257362
C        5.689495    9.973374    7.420336
H        5.920053    9.958068    6.370002
```

```
N        9.296449    5.979034   10.312641
C        8.089911    5.437679   10.029888
N        8.207600    4.870362    8.807922
C        9.488863    5.076361    8.310443
H        9.775803    4.742010    7.333252
C        9.627821    6.716211   11.529706
H       10.229694    7.579053   11.263117
H       10.155297    6.070999   12.229488
H        8.707349    7.074551   11.983050
C        7.138328    4.180589    8.089988
H        7.180791    4.463292    7.043103
H        6.181595    4.507571    8.488672
H        7.237662    3.103517    8.209403
C       10.170425    5.770507    9.252396
H       11.168671    6.160996    9.258260
```

Braunschweig neutral Mn B=Se

```
45
Se       1.066006    4.559700    2.448042
O        2.610727    4.635385    6.377499
C        2.383635    5.234914    5.380938
Mn       2.047270    6.286148    4.027820
B        2.028762    2.953959    2.259804
C        3.689857    6.258154    3.436748
O        4.818610    6.360888    3.091469
C        1.616237    1.724353    1.330961
C        0.308885    1.966373    0.558867
H        0.068655    1.096405   -0.061450
H        0.390202    2.840064   -0.090054
H       -0.524593    2.143953    1.240610
C        2.751487    1.452203    0.312594
H        2.489466    0.595932   -0.316662
H        3.696645    1.217656    0.811439
H        2.908772    2.311987   -0.343052
C        1.447652    0.459439    2.209474
H        1.184666   -0.397453    1.581459
H        0.649738    0.592035    2.944049
H        2.370831    0.208221    2.740339
C        0.262110    7.232148    4.858703
H       -0.498558    6.745552    5.441163
C        0.240913    7.438044    3.456881
H       -0.515832    7.074179    2.784417
C        1.409699    8.144481    3.074314
H        1.662298    8.464524    2.080198
C        2.164512    8.401682    4.267252
H        3.084935    8.954986    4.324187
C        1.459980    7.840548    5.364587
H        1.751756    7.895951    6.398104
C        3.361866    2.830942    3.106489
N        3.473422    2.397603    4.383455
C        4.776220    2.575935    4.833090
H        5.064081    2.337336    5.837654
N        4.593696    3.257921    2.744498
C        5.477234    3.114334    3.807511
H        6.497257    3.436994    3.741082
C        4.943628    3.825397    1.444758
H        5.609408    4.668294    1.598617
H        4.037947    4.192627    0.969157
H        5.407945    3.068406    0.815674
C        2.382285    1.861017    5.192918
H        2.467970    2.255286    6.200236
H        2.412778    0.773195    5.193736
H        1.438040    2.201601    4.776122
```

Braunschweig neutral Mn B=Te

```
45
Te       1.304180    1.988061    9.954670
O       -2.661073    0.392295    9.256698
C       -1.536470    0.387853    8.881526
Mn       0.079772    0.206388    8.254023
B        0.222078    3.810279   10.067756
C       -0.166610    1.314126    6.930756
O       -0.342316    1.962827    5.954195
C        1.780710   -0.877887    7.429627
H        2.592755   -0.451131    6.870259
C        0.541688   -1.362337    6.891791
H        0.264051   -1.365887    5.852956
C       -0.231985   -1.878939    7.964240
H       -1.197924   -2.343840    7.880766
C        0.523232   -1.718690    9.174105
H        0.223756   -2.034637   10.156454
C        1.758622   -1.115585    8.827793
H        2.537713   -0.847252    9.518875
C        0.603964    5.087309   10.941830
C        0.773822    6.307415   10.000453
H        1.011964    7.198016   10.589834
H       -0.140874    6.518006    9.438904
H        1.589116    6.149069    9.290881
C        1.895010    4.913307   11.758153
H        2.103106    5.820259   12.335012
H        2.749245    4.716070   11.108248
H        1.812789    4.075666   12.452853
C       -0.561462    5.393704   11.917412
H       -0.323050    6.282360   12.509606
H       -0.725786    4.565228   12.610175
H       -1.495677    5.592993   11.384627
C       -1.090044    3.879586    9.186418
N       -2.329638    3.466241    9.542120
C       -3.190295    3.557604    8.455178
H       -4.209541    3.231522    8.514945
N       -1.176687    4.253464    7.887723
C       -2.469419    4.049835    7.420694
H       -2.735676    4.238492    6.399765
C       -2.710754    2.954217   10.856077
H       -3.366064    2.100151   10.719818
H       -3.196851    3.734583   11.438372
H       -1.816751    2.614611   11.372563
C       -0.073951    4.755837    7.071948
H       -0.139671    4.309078    6.085317
H        0.865160    4.443724    7.521344
H       -0.110961    5.842006    7.015542
```

4. X-ray Crystallographic Analysis

4.1. General Information

Data collections were performed by mounting single crystals on glass fibers or MiTeGen mounts in perfluorinated oil. Diffractometers used for intensity measurements (at 100 K) were Oxford Diffraction Xcalibur E with Mo K_α radiation or Rigaku XtaLAB Synergy S Single Source with either Mo K_α or Cu K_α micro sources. Absorption correction was applied based on multi-scan methods. Data reduction was performed using the program CrysalisPro.[15] The structures were solved with SHELXT-14/5.[16] and refined anisotropically on F^2 using the programs SHELXL-14/7 and SHELXL-17/1 [17] employing the graphical surfaces WINGX [18] or Olex² Crystallography Software.[19]

*4.2. Compound **6**.*

CCDC entry code:	2027614	
Empirical formula:	$C_{27}H_{45}IN_4$	
Formula weight:	552.57	
Temperature:	100.00(10) K	
Wavelength:	1.54184 Å	
Crystal system:	Triclinic	
Space group:	*P*-1, *Z* = 4	
Unit cell dimensions:	*a* = 11.2124(2) Å	α = 71.246(2)°
	b = 16.0192(3) Å	β = 89.5310(10)°
	c = 16.5432(3) Å	γ = 88.9930(10)°
Volume:	2813.18(9) Å^3	
Density (calculated):	1.305 g/cm^3	
Absorption coefficient:	9.079 mm^{-1}	
F(000):	1152	
Theta range for data collection:	2.821 to 74.492°.	
Index ranges:	-14<=h<=14, -18<=k<=20, -20<=l<=20	
Reflections collected:	57855	
Independent reflections:	11401 [R(int) = 0.0563]	
Completeness to theta = 67.684°:	99.8 %	
Absorption correction:	Semi-empirical from equivalents	
Max. and min. transmission:	1.00000 and 0.38687	
Refinement method:	Full-matrix least-squares on F^2	
Data / restraints / parameters:	11401 / 2 / 609	
Goodness-of-fit on F^2:	1.018	
Final R indices [I>2sigma(I)]:	R_1 = 0.0592, wR_2 = 0.1626	
R indices (all data):	R_1 = 0.0638, wR_2 = 0.1668	
Largest diff. peak and hole:	3.200 and -1.642 e.Å^{-3}	

----

*4.3. Compound **7-BBr₄**.*

CCDC entry code:	2027606
Empirical formula:	$C_{27}H_{43}B_2Br_5N_4$
Formula weight:	844.82
Temperature:	100(2) K
Wavelength:	0.71073 Å
Crystal system:	Monoclinic
Space group:	$P2_1/c$, Z = 4
Unit cell dimensions:	a = 8.5096(2) Å α = 90°
	b = 16.1289(4) Å β = 98.289(2)°
	c = 25.3325(6) Å γ = 90°
Volume:	3440.58(14) $Å^3$
Density (calculated):	1.631 g/cm^3
Absorption coefficient:	5.863 mm^{-1}
F(000):	1672
Theta range for data collection:	2.526° to 26.370°
Completeness to theta = 25.242°:	99.9 %
Index ranges:	-10 <= h <= 10, -20 <= k <= 20, -31 <= l <= 31
Reflections collected: / indep. / obs.	11825
Independent reflections:	11825 (Rint = 0.0942)
Max. and min. transmission:	0.991 and 0.923
Refinement method:	Full-matrix least-squares on F^2
Data / restraints / parameters:	11825 / 0 / 350
Goodness-of-fit on F^2:	1.045
Final R indices [I>2sigma(I)]:	R_1 = 0.0365, wR_2 = 0.1056
R indices (all data):	R_1 = 0.0412, wR_2 = 0.1079
Largest diff. peak and hole:	1.479 and -1.686 e.$Å^{-3}$

----

*4.4. Compound **7-Br**.*

CCDC entry code:	2027609
Empirical formula:	$C_{58}H_{96}B_2Br_4N_8O$ ≡ (**7-Br**)$_2$ · Et_2O
Formula weight:	1262.68
Temperature:	100.0(3) K
Wavelength:	0.71073 Å
Crystal system:	Trigonal
Space group:	*P*32, *Z* = 3
Unit cell dimensions:	*a* = 17.67270(10) Å α = 90°
	b = 17.67270(10) Å β = 90°
	c = 17.60890(10) Å γ = 120°
Volume:	4762.87(6) Å^3
Density (calculated):	1.321 g/cm^3
Absorption coefficient:	2.578 mm^{-1}
F(000):	1974
Theta range for data collection:	2.579 to 26.371°.
Index ranges:	-22<=h<=22, -22<=k<=22, -22<=l<=22
Reflections collected:	191395
Independent reflections:	12987 [R(int) = 0.0401]
Completeness to theta = 25.242°:	99.9 %
Absorption correction:	Semi-empirical from equivalents
Max. and min. transmission:	1.00000 and 0.78069
Refinement method:	Full-matrix least-squares on F^2
Data / restraints / parameters:	12987 / 1 / 685
Goodness-of-fit on F^2:	1.069
Final R indices [I>2sigma(I)]:	R_1 = 0.0230, wR_2 = 0.0634
R indices (all data):	R_1 = 0.0236, wR_2 = 0.0637
Absolute structure parameter:	0.005(5)
Largest diff. peak and hole:	0.674 and -0.491 e.Å^{-3}

---

*4.5. Compound **8**.*

CCDC entry code:	2027607
Empirical formula:	$C_{27}H_{44}BBrN_4O$
Formula weight:	531.38
Temperature :	298(2) K
Wavelength:	0.71073 Å
Crystal system:	Triclinic
Space group:	*P*1, *Z* = 2
Unit cell dimensions:	*a* = 8.73270(10) Å α = 117.516(2)°
	b = 13.5981(2) Å β = 99.7310(10)°
	c = 13.6474(2) Å γ = 94.4720(10)°
Volume:	1393.84(4) $Å^3$
Density (calculated):	1.266 g/cm^3
Absorption coefficient:	1.500 mm^{-1}
F(000):	564
Theta range for data collection:	2.694 to 31.122°.
Index ranges:	-12<=h<=12, -18<=k<=19, -19<=l<=18
Reflections collected:	62119
Independent reflections:	13881 [R(int) = 0.0275]
Completeness to theta = 25.242°:	99.9 %
Refinement method:	Full-matrix least-squares on F^2
Data / restraints / parameters:	13881 / 3 / 569
Goodness-of-fit on F^2:	1.044
Final R indices [I>2sigma(I)]:	R_1 = 0.0355, wR_2 = 0.0894
R indices (all data):	R_1 = 0.0438, wR_2 = 0.0941
Absolute structure parameter:	0.486(13)
Largest diff. peak and hole:	0.845 and -0.470 $e.Å^{-3}$

---

*4.6. Compound **9**.*

CCDC entry code:	2027610	
Empirical formula:	$C_{54}H_{86}B_2Br_2Li_2N_8O_2$	
Formula weight:	1074.62	
Temperature:	100(2) K	
Wavelength:	1.54184 Å	
Crystal system:	Monoclinic	
Space group:	*C*2/*c*, *Z* = 4	
Unit cell dimensions:	*a* = 25.1768(3) Å	α = 90°
	b = 10.81621(10) Å	β = 90.2610(10)°
	c = 23.3489(3) Å	γ = 90°
Volume:	6358.24(12) $Å^3$	
Density (calculated):	1.123 g/cm^3	
Absorption coefficient:	1.925 mm^{-1}	
F(000):	2272	
Theta range for data collection:	3.511 to 77.585°.	
Index ranges:	-31<=h<=31, -13<=k<=13, -26<=l<=29	
Reflections collected:	128887	
Independent reflections:	6719 [R(int) = 0.0490]	
Completeness to theta = 67.684°:	100.0 %	
Refinement method:	Full-matrix least-squares on F^2	
Data / restraints / parameters:	6719 / 0 / 328	
Goodness-of-fit on F^2:	1.101	
Final R indices [I>2sigma(I)]:	R_1 = 0.0322, wR_2 = 0.0909	
R indices (all data):	R_1 = 0.0338, wR_2 = 0.0921	
Largest diff. peak and hole:	0.345 and -0.740 e.$Å^{-3}$	

---

*4.7. Compound **10**.*

CCDC entry code:	2027611
Empirical formula :	$C_{27}H_{43}BN_4O$
Formula weight:	450.46
Temperature :	100(2) K
Wavelength:	1.54184 Å
Crystal system:	Trigonal
Space group:	*R*−3, *Z* = 18
Unit cell dimensions:	*a* = 29.5459(4) Å α = 90°
	b = 29.5459(4) Å β = 90°
	c = 16.7972(2) Å γ = 120°
Volume:	12698.8(4) $Å^3$
Density (calculated):	1.060 g/cm^3
Absorption coefficient:	0.495 mm^{-1}
F(000):	4428
Theta range for data collection:	2.991 to 77.627°.
Index ranges:	-34<=h<=37, -37<=k<=37, -21<=l<=21
Reflections collected:	87610
Independent reflections:	5987 [R(int) = 0.0381]
Completeness to theta = 67.684°:	100.0 %
Refinement method:	Full-matrix least-squares on F^2
Data / restraints / parameters:	5987 / 0 / 310
Goodness-of-fit on F^2:	1.044
Final R indices [I>2sigma(I)]:	R_1 = 0.0473, wR_2 = 0.1290
R indices (all data):	R_1 = 0.0501, wR_2 = 0.1311
Largest diff. peak and hole:	0.249 and -0.291 e.$Å^{-3}$

---

*4.8. Compound **11**.*

CCDC entry code:	2027608
Empirical formula:	$C_{33}H_{49}BN_4S$ ≡ **11** · C_6H_6
Formula weight:	544.63
Temperature:	99.9(6) K
Wavelength:	1.54184 Å
Crystal system:	Monoclinic
Space group:	$P2_1/c$, Z = 4
Unit cell dimensions:	a = 10.6234(2) Å α = 90°
	b = 24.7853(6) Å β = 92.8606(18)°
	c = 12.4183(2) Å γ = 90°
Volume:	3265.72(11) $Å^3$
Density (calculated):	1.108 g/cm^3
Absorption coefficient:	1.066 mm^{-1}
F(000):	1184
Theta range for data collection:	3.567 to 77.631°.
Index ranges:	-13<=h<=13, -31<=k<=31, -15<=l<=15
Reflections collected:	13121
Independent reflections:	13121 [R(int) = 0.0590]
Completeness to theta = 67.684°:	100.0 %
Refinement method:	Full-matrix least-squares on F^2
Data / restraints / parameters:	13121 / 0 / 365
Goodness-of-fit on F^2:	1.045
Final R indices [I>2sigma(I)]:	R_1 = 0.0338, wR_2 = 0.0925
R indices (all data):	R_1 = 0.0355, wR_2 = 0.0936
Largest diff. peak and hole:	0.262 and -0.194 e.$Å^{-3}$

---

*4.9. Compound **12**.*

CCDC entry code:	2027612
Empirical formula:	$C_{45}H_{61}BN_4Se$ ≡ **12** · 3 C_6H_6
Formula weight:	747.74
Temperature:	298(2) K
Wavelength:	0.71073 Å
Crystal system:	Orthorhombic
Space group:	*Pnma*, *Z* = 4
Unit cell dimensions:	*a* = 18.1400(2) Å α = 90°
	b = 24.8754(3) Å β = 90°
	c = 9.40470(10) Å γ = 90°
Volume:	4243.77(8) $Å^3$
Density (calculated):	1.170 g/cm^3
Absorption coefficient:	0.919 mm^{-1}
F(000):	1592
Theta range for data collection:	2.573 to 31.009°.
Index ranges:	-24<=h<=22, -33<=k<=33, -11<=l<=12
Reflections collected:	92625
Independent reflections:	6204 [R(int) = 0.0297]
Completeness to theta = 25.242°:	99.9 %
Refinement method:	Full-matrix least-squares on F2
Data / restraints / parameters:	6204 / 3 / 284
Goodness-of-fit on F^2:	1.042
Final R indices [I>2sigma(I)]:	R_1 = 0.0400, wR_2 = 0.1057
R indices (all data):	R_1 = 0.0482, wR_2 = 0.1102
Largest diff. peak and hole:	0.798 and -0.549 e.$Å^{-3}$

---

*4.10. Compound **13**.*

CCDC entry code:	2027613	
Empirical formula:	$C_{45}H_{61}BN_4Te$ ≡ **13** · 3 C_6H_6	
Formula weight:	796.38	
Temperature:	100.00(10) K	
Wavelength:	0.71073 Å	
Crystal system:	Orthorhombic	
Space group:	$P2_12_12_1$, Z = 12	
Unit cell dimensions:	*a* = 18.3769(4) Å	α = 90°
	b = 24.6599(6) Å	β = 90°
	c = 28.6576(6) Å	γ = 90°
Volume:	12986.8(5) Å^3	
Density (calculated):	1.222 g/cm^3	
Absorption coefficient:	0.721 mm^{-1}	
F(000):	4992	
Theta range for data collection:	2.541 to 31.247°.	
Index ranges:	-25<=h<=23, -33<=k<=34, -38<=l<=41	
Reflections collected:	303639	
Independent reflections:	36459 [R(int) = 0.0487]	
Completeness to theta = 25.242°:	99.8 %	
Refinement method:	Full-matrix least-squares on F2	
Data / restraints / parameters:	36459 / 72 / 1391	
Goodness-of-fit on F^2:	1.035	
Final R indices [I>2sigma(I)]:	R_1 = 0.0659, wR_2 = 0.1711	
R indices (all data):	R_1 = 0.0899, wR_2 = 0.1856	
Largest diff. peak and hole:	2.765 and -0.656 e.Å^{-3}	

---

*4.11. Compound **14**.*

CCDC entry code:	2045657
Empirical formula:	C31 H53 B2 F4 N5
Formula weight:	593.40
Temperature:	100(2) K
Wavelength:	1.54184 Å
Crystal system:	Monoclinic
Space group:	$P2_1$/c, Z = 8
Unit cell dimensions:	a = 8.9678(2) Å α = 90°
	b = 24.5277(4) Å β = 91.361(2)°
	c = 33.6924(7) Å γ = 90°
Volume:	7408.9(3) $Å^3$
Density (calculated):	1.064 g/cm^3
Absorption coefficient:	0.622 mm^{-1}
F(000):	2560
Theta range for data collection:	2.228 to 77.664°.
Index ranges:	-11<=h<=11, -31<=k<=29, -41<=l<=42
Reflections collected:	150934
Independent reflections:	5534 [R(int) = 0.0894]
Completeness to theta = 67.684°:	99.9 %
Refinement method:	Full-matrix least-squares on F^2
Data / restraints / parameters:	15534 / 0 / 832
Goodness-of-fit on F^2:	1.027
Final R indices [I>2sigma(I)]:	R_1 = 0.0895, wR_2 = 0.2387
R indices (all data):	R_1 = 0.0995, wR_2 = 0.2458
Largest diff. peak and hole:	0.699 and -0.434 e.$Å^{-3}$

---

*4.12. Compound **15**.*

CCDC entry code:	2045658
Empirical formula:	$C_{34}H_{48}BCl_3N_4O_2$ ≡ **15** · $CHCl_3$
Formula weight:	661.92
Temperature:	100.00(10) K
Wavelength :	0.71073 Å
Crystal system:	Monoclinic
Space group:	$P2_1$, Z = 2
Unit cell dimensions:	a = 10.3013(3) Å α =90°
	b = 17.8595(3) Å β = 113.336(3)°
	c = 10.4602(3) Å γ = 90°
Volume:	1767.00(9) Å^3
Density (calculated):	1.244 g/cm^3
Absorption coefficient:	0.295 mm^{-1}
F(000):	704
Theta range for data collection:	2.611 to 31.638°.
Index ranges:	-14<=h<=14, -25<=k<=24, -14<=l<=14
Reflections collected:	83739
Independent reflections:	10226 [R(int) = 0.0469]
Completeness to theta = 25.242°:	99.8 %
Refinement method:	Full-matrix least-squares on F^2
Data / restraints / parameters:	10226 / 1 / 410
Goodness-of-fit on F^2:	1.061
Final R indices [I>2sigma(I)]:	R_1 = 0.0358, wR_2 = 0.0936
R indices (all data):	R_1 = 0.0384, wR_2 = 0.0951
Absolute structure parameter:	0.32(4)
Largest diff. peak and hole:	0.588 and -0.407 e.Å^{-3}

---

*4.13. Compound **16**.*

CCDC entry code:	2045659	
Empirical formula:	$C_{29}H_{43}BN_6$	
Formula weight:	486.50	
Temperature:	100(2) K	
Wavelength:	1.54184 Å	
Crystal system:	Monoclinic	
Space group:	*I*2/a, *Z* = 8	
Unit cell dimensions:	*a* = 15.98950(10) Å	α = 90°
	b = 11.87200(10) Å	β = 92.0010(10)°
	c = 30.8634(2) Å	γ = 90°
Volume:	5855.14(7) $Å^3$	
Density (calculated):	1.104 g/cm^3	
Absorption coefficient:	0.508 mm^{-1}	
F(000):	2112	
Theta range for data collection:	2.865 to 77.485°.	
Index ranges:	-19<=h<=20, -14<=k<=15, -39<=l<=37	
Reflections collected:	60598	
Independent reflections:	6157 [R(int) = 0.0287]	
Completeness to theta = 67.684°:	100.0 %	
Refinement method:	Full-matrix least-squares on F^2	
Data / restraints / parameters:	6157 / 0 / 337	
Goodness-of-fit on F^2:	1.046	
Final R indices [I>2sigma(I)]:	R_1 = 0.0437, wR_2 = 0.1108	
R indices (all data):	R_1 = 0.0463, wR_2 = 0.1137	
Largest diff. peak and hole:	0.291 and -0.265 e.$Å^{-3}$	

Author Contributions

Hadi Dolati (lead synthetic work, writing of original draft), Lars Denker (synthetic work, X-ray crystallographic work including structural solution), Dr. Bartosz Trzaskowski (computational work, formal analysis, funding acquisition, project administration), Dr. René Frank (synthetic work, formal analysis, funding acquisition, project administration).

---

[1] M. Tamm, D. Petrovic, S. Randoll, S. Beer, T. Bannenberg, P. G. Jones, J. Grunenberg, *Org. & Biomol. Chem.* **2007**, 53, 523−530.
[2] J. Młochowski, L. Syper, Entry for Li_2Se in Encyclopedia of Reagents for Organic Synthesis, https://doi.org/10.1002/047084289X.rl141; b) E. Zintl, A. Harder, B. Dauth, *Z. Elektrochem.* **1934**, *40*, 588.
[3] J-D. Chai, M. Head-Gordon, *Phys. Chem. Chem. Phys.* **2008**, 10, 6615−6620.
[4] Y. Zhao, D. G. Truhlar, *Theor. Chem. Acc.* **2008**, 120, 215−241.
[5] T. Yanai, D. P. Tew, N. C. Handy, *Chem. Phys. Lett.* **2004**, 393, 51−57.
[6] W. R. Wadt, P. J. Hay, *J. Chem. Phys.* **1985**, 82, 284.
[7] S Huzinaga, B. Miguel, *Chem. Phys. Lett.* **1990**, 175, 289−291.
[8] S. Huzinaga, M. Klobukowski, *Chem. Phys. Lett.* **1993**, 212, 260−264.
[9] B. P. Pritchard, D. Altarawy, B. Didier, T. D. Gibson, T. L. Windus, *J. Chem. Inf. Model.* **2019**, 11, 4814−4820.
[10] Gaussian 09, Revision D.01, M. J. Frisch, G. W. Trucks, H. B. Schlegel, G. E. Scuseria, M. A. Robb, J. R. Cheeseman, G. Scalmani, V. Barone, B. Mennucci, G. A. Petersson, H. Nakatsuji, M. Caricato, X. Li, H. P. Hratchian, A. F. Izmaylov, J. Bloino, G. Zheng, J. L. Sonnenberg, M. Hada, M. Ehara, K. Toyota, R. Fukuda, J. Hasegawa, M. Ishida, T. Nakajima, Y. Honda, O. Kitao, H. Nakai, T. Vreven, J. A. Montgomery, Jr., J. E. Peralta, F. Ogliaro, M. Bearpark, J. J. Heyd, E. Brothers, K. N. Kudin, V. N. Staroverov, R. Kobayashi, J. Normand, K. Raghavachari, A. Rendell, J. C. Burant, S. S. Iyengar, J. Tomasi, M. Cossi, N. Rega, J. M. Millam, M. Klene, J. E. Knox, J. B. Cross, V. Bakken, C. Adamo, J. Jaramillo, R. Gomperts, R. E. Stratmann, O. Yazyev, A. J. Austin, R. Cammi, C. Pomelli, J. W. Ochterski, R. L. Martin, K. Morokuma, V. G. Zakrzewski, G. A. Voth, P. Salvador, J. J. Dannenberg, S. Dapprich, A. D. Daniels, Ö. Farkas, J. B. Foresman, J. V. Ortiz, J. Cioslowski, D. J. Fox, Gaussian, Inc., Wallingford CT, 2009.
[11] J. M. Foster, S. F. Boys, *Rev. Mod. Phys.* **1960**, 32, 300.
[12] T. Lu, F. Chen, *J. Comput. Chem.* **2012**, 33, 580−592.
[13] W. Humphrey, A. Dalke, K. Schulten, *J. Mol. Graph.* **1996**, 14, 33−38.
[14] Y. K. Loh, K. Porteous, M. Angeles Fuentes, D. C. H. Do, J. Hicks, S. Aldridge, *J. Am. Chem. Soc.* **2019**, 141, 8073−8077
[15] Rigaku Oxford Diffraction, "CrysAlisPRO Software System version 1.171.39.46", **2018.** Rigaku Corporation, Oxford, UK. (Ed.).
[16] G. M. Sheldrick, *Acta Cryst.* **2015**, *A71*, 3–8.
[17] G. M. Sheldrick, *Acta Crystallogr., Sect. A: Found. Crystallogr.* **2008**, *64*, 112–122.
[18] L. J. Farrugia, *J. Appl. Cryst.* (2012),**45**, 849-854.
[19] https://www.olexsys.org/.
